# Protonolysis and Condensation Reactions of Alkoxido-Substituted Lindqvist {MW_5_} and Keggin {MPW_11_} Polyoxometalates: Comparative Experimental and Modeling Studies

**DOI:** 10.1021/acs.inorgchem.4c04636

**Published:** 2025-01-29

**Authors:** Daniel Lebbie, Thompson Izuagie, Magda Pascual-Borràs, Balamurugan Kandasamy, Corinne Wills, Paul G. Waddell, Benjamin R. Horrocks, R. John Errington

**Affiliations:** NUPOM Lab, Chemistry, School of Natural and Environmental Sciences, Newcastle University, Newcastle upon Tyne NE1 7RU, U.K.

## Abstract

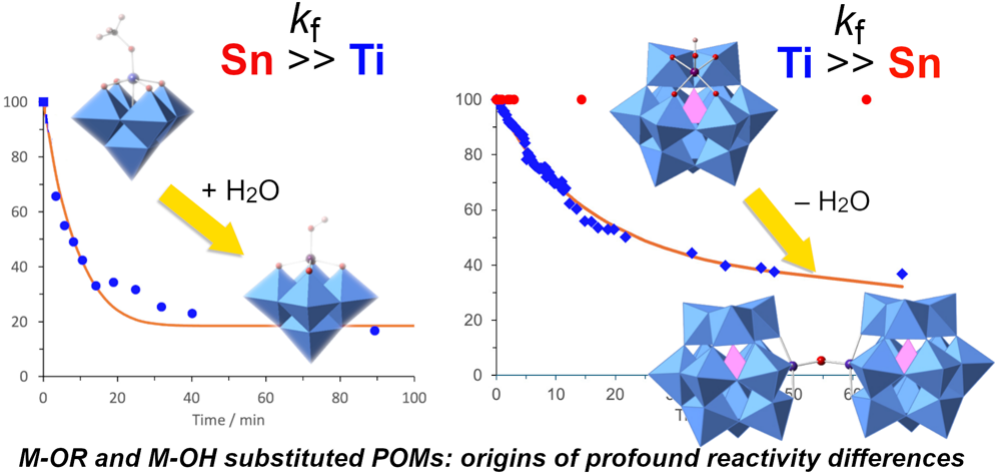

An understanding of proton transfer and migration at the surfaces of solid metal oxides and related molecular polyoxometalates (POMs) and metal alkoxides is crucial for the development of reactivity involving protonation or the absorption/binding of water. In this work, the hydrolysis of alkoxido Ti- and Sn-substituted Lindqvist [(MeO)MW_5_O_18_]^3–^ (M = Ti, **1**; M = Sn, **2**) and Keggin [(MeO)MPW_11_O_39_]^4–^ (M = Ti, **3**; M = Sn, **4**) type polyoxometalates (POMs) to hydroxido derivatives and subsequent condensation to μ-oxido species has been investigated in detail to provide insight into proton transfer reactions in these molecular metal oxide systems. Solution NMR studies revealed the dependence of reactions not only on the nature of the heteroatom (Ti or Sn) but also on the type of lacunary (W_5_ or PW_11_) POM and also on the solvent (MeCN or DMSO). Tin-substituted anions **2** and **4** were much more susceptible to protonolysis than the Ti analogues **1** and **3** while reactions of {MW_5_} anions were generally faster than those of the {MPW_11_} anions. Subsequent condensation of the resulting hydroxido derivatives [(HO)MW_5_O_18_]^3–^ (M = Ti, **5**; M = Sn, **6**) and [(HO)MPW_11_O_39_]^4–^ (M = Ti, **7**; M = Sn, **8**) was significantly more facile for **5** and **7** and, in all cases, condensation was inhibited in DMSO. Quantitative comparisons of equilibria and reaction rates were provided by analysis of NMR kinetic experiments, while DFT calculations on these and the analogous {NbW_5_} reactions provided comparative energetics and reaction profiles that are consistent with experimental observations. These results add to the fundamental understanding of proton migration in metal alkoxide hydrolysis/condensation and related reactions at metal oxide surfaces.

## Abstract 2

Alcoholysis and hydrolysis of M−OMe bonds in substituted Lindqvist and Keggin type polyoxometalates was studied and condensation of M−OH derivatives to MOM-bridged products was compared. NMR and kinetics analysis showed that Lindqvist {MW_5_} anions are more reactive than {MPW_11_} Keggin anions, protonolysis is faster for Sn than for Ti derivatives, and SnOSn linkages are less stable than TiOTi. DFT modeling provided a mechanistic understanding with energetics that are consistent with the experimental observations.

## Introduction

The diverse and versatile polyoxometalates (POMs) of V, Nb, Ta, Mo and W are attractive as well-defined molecular models for solid metal oxides, where the complex nature of oxide surfaces remains a significant challenge in the elucidation of detailed surface reaction mechanisms. Despite this, the capacity of POMs to mimic solid oxide reactivity has yet to be fully explored. An intrinsic aspect of metal oxide chemistry is the adsorption and dissociation of water to give surface M–OH groups, a crucial process in heterogeneous catalysis, geochemistry and the reactivity of metal oxide nanoparticles. The related POM chemistry is therefore of significant interest, but important aspects of POM reactivity involving proton transfer are still not fully understood. The formation and subsequent condensation of transient hydroxido species generated upon protonation of monomeric oxometalates [VO_4_]^3–^ and [MO_4_]^2–^ (M = Mo, W) is assumed to be the initial step in the primary “self-assembly” of POMs via formation of M–O–M linkages, while the protonation of preformed POM structures and subsequent condensation provides secondary assembly processes that can result in more elaborate architectures based on POM “building blocks”. Mechanistic details for the formation of even the most common POM structures are lacking, although there has been significant progress in the computational modeling of solution dynamics in POM “self-assembly”.^1−3^ In the related “sol–gel” production of oxide materials from metal alkoxides, different kinetic regimes have been proposed for the initial hydrolytic formation of primary polynuclear oxoalkoxide particles and the subsequent slower aggregation processes that lead to extended gel structures, but detailed studies of proton transfer are challenging due to the metal alkoxide multifunctionality, i.e., the availability of multiple reactive M–OR bonds at each metal center.^4−6^ Studies of POMs containing hydroxido or alkoxido substituents should therefore provide valuable insight into the reactivity of metal oxide surfaces and fundamental steps in the sol–gel process. With this in mind, we have developed nonaqueous routes to a range of alkoxido-substituted hexanuclear Lindqvist derivatives.^7−9^

**Scheme 1:**
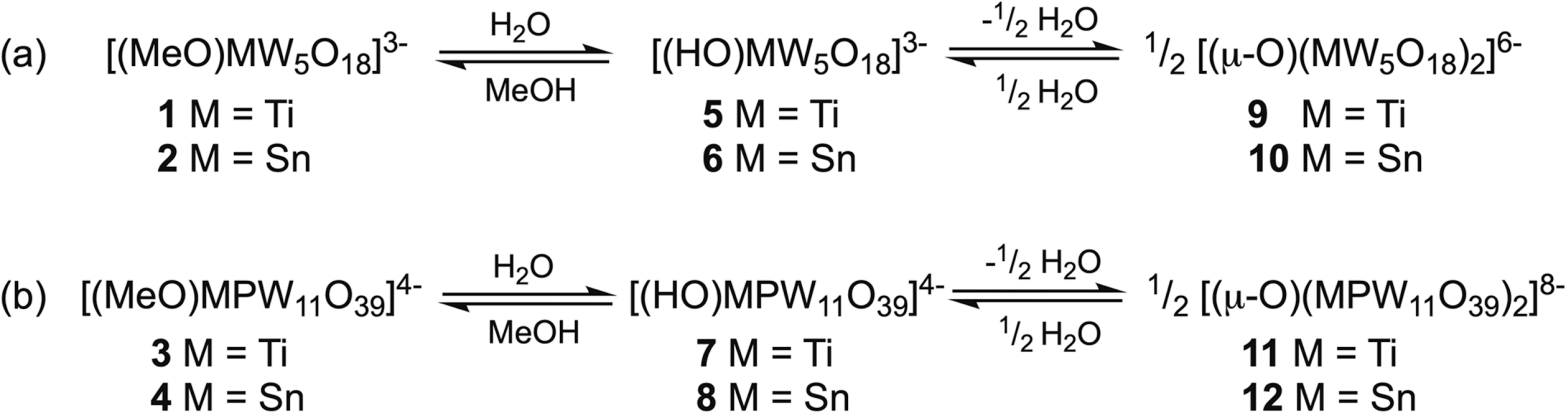
Hydrolysis and Condensation of Lindqvist-Type {(MeO)MW_5_} (a) and Keggin-type {(MeO)MPW_11_} (b) Polyoxometalates (M = Ti, Sn)

**Figure 1:**
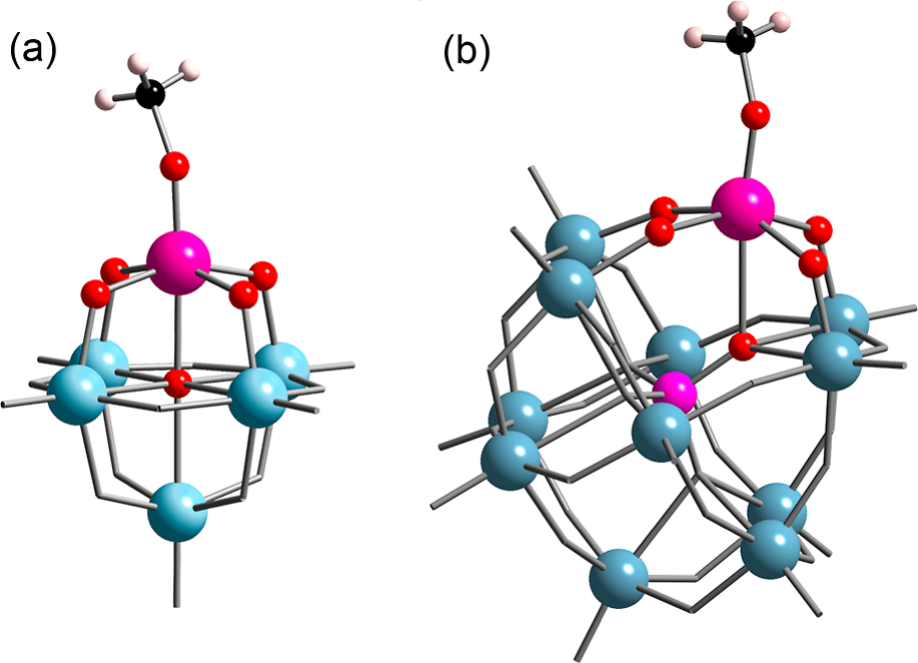
Representative structures of (a) Lindqvist-type [(MeO)M^IV^W_5_O_18_]^3–^ and (b) Keggin-type [(MeO)M^IV^PW_11_O_39_]^4–^ anions, highlighting the alkoxido-substituted heterometal sites.

In classical molybdate and tungstate structures such as the Lindqvist [M_6_O_19_]^2−^ and Keggin [EM_12_O_40_]^*n*−^ anions (where E is a central tetrahedral heteroatom) the addenda Mo or W atoms are multiply bonded to terminal oxido ligands, but substitution by heterometal atoms M′ that prefer to form M′–O σ bonds rather than σ + π multiple bonds, e.g., Nb(V), Ti(IV), Ru(IV)/(III) or Fe(III), enables the isolation of aggregates containing regiospecific M′–(μ-O)–M′ linkages. Structurally characterized examples include [(μ-O)(NbW_5_O_18_)_2_]^4–^,^7,10^ [(μ-O)(TiW_5_O_18_)_2_]^6–^,^11^ [(μ-O)(TiMo_5_O_18_)_2_]^6–^,^12^ [(μ-O)(TiPW_11_O_39_)_2_]^8–^,^13^ [(μ-O)(RuSiW_11_O_39_)_2_]^10–^,^14^ and [(μ-O)(FePW_11_O_39_)_2_]^8–^.^15^ In aqueous solutions, the putative hydroxido precursors to such aggregates may be generated at low pH by protonation of basic terminal M′=O groups (e.g., Ti=O in [TiPW_11_O_40_]^5–^),^16,17^ or at higher pH by deprotonation of H_2_O bound to Lewis acidic heterometal sites, as observed for [(H_2_O)RuSiW_11_O_39_]^5–^ or [(H_2_O)FePW_11_O_39_]^4–^.^14,18^ In nonaqueous solvents, hydroxido groups may also be generated by hydrolysis of M′–OR bonds as in the sol–gel process. Protonolysis of the M′–OR bonds in alkoxido-substituted heterometallic Lindqvist-type [(RO)M′M_5_O_18_]^*n*−^ (M = Mo or W) provides a platform for the systematic manipulation of organic–inorganic hybrid POMs, and we have elucidated some of the factors affecting their solution reactivities.^11,12,19,20^ We now report results from detailed investigations of methanol exchange and hydrolysis–condensation reactions (Scheme 1) of tetrabutylammonium (TBA) salts of the Lindqvist-type methoxido anions [(MeO)TiW_5_O_18_]^3–^**1** and [(MeO)SnW_5_O_18_]^3–^**2**, and the related Keggin-type anions [(MeO)TiPW_11_O_39_]^4–^**3** and [(MeO)SnPW_11_O_39_]^4–^**4** (Figure 1). Following a brief preliminary report,^21^ full details are presented here of systematic studies involving the terminal hydroxides (TBA)_3_[(HO)TiW_5_O_18_] (TBA)_3_**5**, (TBA)_3_[(HO)SnW_5_O_18_] (TBA)_3_**6**, (TBA)_4_[(HO)TiPW_11_O_39_] (TBA)_4_**7** and (TBA)_4_[(HO)SnPW_11_O_39_] (TBA)_4_**8** and the condensation reactions leading to the oxo-bridged (TBA)_6_[(μ-O)(TiW_5_O_18_)_2_] (TBA)_6_**9**, (TBA)_6_[(μ-O)(SnW_5_O_18_)_2_] (TBA)_6_**10**, (TBA)_8_[(μ-O)(TiPW_11_O_39_)_2_] (TBA)_8_**11**, and (TBA)_8_[(μ-O)(SnPW_11_O_39_)_2_] (TBA)_8_**12**, together with kinetic analysis and results from DFT modeling of the proton-transfer reactions involved in these fundamental transformations. The calculated energetics and proposed mechanisms are consistent with our experimental observations and provide an insight into the influence not only of the heterometal site but also of the nature of the lacunary POM “ligand” on the comparative reactivities of these substituted POMs.

## Experimental Section

### General

All reactions and manipulations were carried out under an atmosphere of dry, oxygen-free nitrogen in screw-top flasks fitted with J Young PTFE screw valves using Schlenk and drybox techniques.^22^ Diethyl ether was dried over and distilled from sodium benzophenone ketyl, acetonitrile was dried over and distilled from calcium hydride, and methanol was predried over 3A molecular sieves and distilled from magnesium methoxide. Dimethyl sulfoxide and deuterated solvents were dried over activated 3A molecular sieves and then degassed. All solvents were stored over activated 3A molecular sieves under dry nitrogen. ^17^O-enriched and nonenriched (TBA)_3_**1** and (TBA)_3_**2** were synthesized from (TBA)_2_[WO_4_] and WO(OMe)_4_ as previously described,^7,9^ and (TBA)_4_**3** and (TBA)_4_**4** were prepared by treating (TBA)_4_[ClMPW_11_O_39_] (M = Ti, Sn) with NaOMe following Knoth’s method.^23,24^ Purities were confirmed by FTIR, ^1^H, ^17^O and ^119^Sn NMR spectroscopy. The tin analogue (TBA)_4_**4** was notably more moisture-sensitive than (TBA)_4_**3**, and trace hydrolysis was only circumvented by treatment of the initial metathesis product with an excess of MeOH (1:1 MeCN/MeOH). ^17^O-enriched (TBA)_4_**3** and (TBA)_4_**4** were obtained from ^17^O-enriched (TBA)_6_[NaPW_11_O_39_] prepared by stirring with ^17^O-enriched water in CH_2_Cl_2_. 10% ^17^O-enriched water and deuterated solvents were purchased from Goss Scientific.

Infrared spectra were recorded on a Bruker Alpha spectrometer fitted with a Platinum ATR module (4000–400 cm^–1^). NMR spectra were recorded on a Bruker Avance III 300 spectrometer operating at 300.0 MHz (^1^H), 121.49 MHz (^31^P), or 111.89 MHz (^119^Sn); a Bruker Avance 400 spectrometer operating at 399.78 MHz (^1^H), 54.20 MHz (^17^O), 161.83 MHz (^31^P), or 149.04 MHz (^119^Sn) or a Bruker Avance III HD 500 spectrometer operating at 500.15 MHz (^1^H), 67.84 MHz (^17^O), 186.40 MHz (^119^Sn) or 20.84 MHz (^183^W). NMR spectra were referenced by sample replacement to SiMe_4_ (^1^H and ^13^C), H_2_O (^17^O), SnMe_4_ (^119^Sn) and aqueous 2 M Na_2_WO_4_ (^183^W). ^1^H and ^13^C NMR resonances due to ^n^Bu_4_N^+^ (TBA) cations are not listed in NMR data given below, but appear as multiplets centered at about 1.0, 1.4, 1.7, and 3.2 ppm in ^1^H NMR spectra and as singlets at about 13, 19, 24, and 58 ppm in ^13^C{^1^H} NMR spectra.

### NMR

2D ^1^H EXSY NMR experiments were recorded using a Bruker Avance III HD 500 NMR spectrometer operating at 333 K. The noesygpph Bruker pulse sequence was used and spectra were recorded with 256 increments, 4 scans per increment, with a relaxation delay of 60 s and a mixing time of 500 ms. Spectra were processed using Topspin. For the kinetic experiments, an accurately weighed amount of the POM was added to a 5 mm NMR tube in a glovebox and dissolved in a measured amount of CD_3_CN or (CD_3_)_2_SO. An ^1^H or ^31^P NMR spectrum was recorded prior to addition of a measured amount of CD_3_OD or H_2_O. The time of addition was noted and spectra were then acquired at intervals. Delay times for ^1^H NMR spectra were adjusted to allow for the previously determined *T*_1_ values for MOMe protons.

### Crystallography

Single crystal diffraction data for (TBA)_3_[(HO)TiW_5_O_18_]·MeCN were collected on an Xcalibur, Atlas, Gemini ultra diffractometer using molybdenum radiation (λ_Mo Kα_ = 0.71073 Å) with intensities corrected for absorption empirically using spherical harmonics. Data for (TBA)_9_[(μ-O)(SnW_5_O_18_)_2_]·[(HO)SnW_5_O_18_]·3MeCN were collected on an XtaLAB Synergy HyPix-Arc 100 diffractometer using copper radiation (λ_Cu Kα_ = 1.54184 Å) with intensities corrected for absorption using a multifaceted crystal model created by indexing the faces of the crystal for which data were collected.^25^ All data were collected at 150 K using an Oxford Cryosystems CryostreamPlus open-flow N_2_ cooling device. Cell refinement, data collection and data reduction were undertaken via the software CrysAlisPro.^26^ All structures were solved using XT^27^ and refined by XL^28^ using the Olex2 interface.^29^ All nonhydrogen atoms were refined anisotropically and hydrogen atoms were positioned with idealized geometry, with the exception of those bound to heteroatoms, the positions of which were located using peaks in the Fourier difference map. The displacement parameters of the hydrogen atoms were constrained using a riding model with *U*_(H)_ set to be an appropriate multiple of the *U*_eq_ value of the parent atom. One-half of the anion **10** in the structure of (TBA)_9_[(μ-O)(SnW_5_O_18_)_2_]·[(HO)SnW_5_O_18_]·3MeCN was observed to be disordered over two positions and was modeled as such. The TBA cations adjacent to this disordered moiety, which were clearly present in the Fourier difference map, were severely disordered as a result and could not be modeled in a sensible way. The associated electron density was hence treated with the Olex2 solvent mask routine.

### Computation

DFT calculations were performed on a series of compounds using the Gaussian 09 suite of programs.^30^ The minima (reactants, intermediates and products) as well as transition states were obtained using the B3LYP hybrid functional with a 6-31G** basis set.^31,32^ For heavy elements, we used the standard double-ζ basis set with LANL pseudopotentials of Hay and Wadt.^33^ Transition states were identified by the characteristic calculation of Hessian matrices with only one imaginary frequency corresponding to the reaction coordinate. The calculations include solvent effects by means of the polarizable continuum model (PCM) to simulate the effects of acetonitrile.^34^

### (TBA)_3_[(HO)TiW_5_O_18_] (TBA)_3_**5**

H_2_O (1.20 mL, 66.47 mmol) was added dropwise to (TBA)_3_**1** (0.688 g, 0.34 mmol) in DMSO (3 mL) and the solution was allowed to stir for 2 h at room temperature before the volatiles were removed in vacuo and the process repeated twice more. The resulting colorless solution was triturated with diethyl ether (60 mL × 3) to give a white solid (0.535 g, 79%).

#### (TBA)_3_[(HO)SnW_5_O_18_] (TBA)_3_**6**

H_2_O (0.86 mL, 47.64 mmol) was added to (TBA)_3_**2** (1.00 g, 0.48 mmol) in MeCN (5 mL) and the solution heated to 85–90 °C for 1 h. Volatiles were removed from the hot solution and the residue was redissolved in MeCN (5 mL). The treatment with H_2_O was repeated twice more before removal of the volatiles in vacuo to give a colorless solid which was washed with diethyl ether (10 mL) and dried in vacuo. Yield: 0.94 g, 95%.

### (TBA)_4_[(HO)TiPW_11_O_39_] (TBA)_4_**7**

(A): H_2_O (1.00 mL, 55.40 mmol) was added to a solution of (TBA)_4_**3** (0.10 g, 0.027 mmol) in MeCN (10 mL). The solution was allowed to stir for 1 h and vacuum-dried to give a white solid. The hydrolysis step was repeated 5 more times to obtain a white solid (0.096 g, 96%).

(B): H_2_O (2 mL, 110.8 mmol) was added to a solution of (TBA)_4_**3** (1.6 g, 0.429 mmol) in DMSO (15 mL). The solution was allowed to stir for 1 h and then vacuum-dried for 4 h to remove MeOH and H_2_O. The resulting colorless solution was triturated with diethyl ether (30 mL × 12) to give a white solid (1.32 g, 83%).

### (TBA)_4_[(HO)SnPW_11_O_39_] (TBA)_4_**8**

H_2_O (7 μL, 0.388 mmol) was added to a solution of (TBA)_4_**4** (0.65 g, 0.171 mmol) dissolved in MeCN (15 mL). The mixture was stirred for 30 min, vacuum-dried for 5 h, washed with diethyl ether (20 mL × 3) and vacuum-dried again before recording the weight of the crude product (0.61 g, 94%). The white solid was recrystallized from hot MeCN solution (0.47 g, 73%). Anal. Calcd for [(C_4_H_9_)_4_N]_4_HOSnPW_11_O_39_: C, 20.32; H, 3.86; N, 1.48. Found: C, 20.36; H, 4.26; N, 1.27.

### (TBA)_4_[(DO)SnPW_11_O_39_]

(TBA)_4_**4** (0.15 g, 0.04 mmol) was dissolved in MeCN (0.5 mL) to give a colorless solution. D_2_O (2 mL, 111 mmol) was added and white precipitate formed immediately. The mixture was allowed to stir for 1 h and vacuum-dried. (0.15 g, 100%). ^31^P NMR (121.49 MHz, CD_3_CN): δ (ppm), −12.60, fwhm = 2.87 Hz ^2^*J*(^119^Sn^31^P) = 34 Hz; ^119^Sn NMR (186.40 MHz, CD_3_CN): δ (ppm), −599.8, fwhm = 19.82 Hz.

### (TBA)_6_[(μ-O)(TiW_5_O_18_)_2_] (TBA)_3_**9**

H_2_O (1.1 mL, 60.94 mmol) was added to a solution of (TBA)_3_**1** (1.2 g, 0.60 mmol) in MeCN (5 mL). The solution was stirred for 8 h at 85–90 °C before removal of the volatiles and drying under vacuum to give a colorless solid (0.98 g, 83%).

### (TBA)_6_[(μ-O)(SnW_5_O_18_)_2_] (TBA)_3_**10**

(A): A solution of (TBA)_3_**6** in benzonitrile (10 mL) was heated in a sealed flask at 110 °C for 2 h with periodic removal of the volatiles. This procedure was repeated multiple times until ^119^Sn NMR showed full conversion to (TBA)_6_**10**.

(B): *N*,*N*′-dicyclohexylcarbodiimide (DCC) (1.84 g, 8.92 mmol) was added to (TBA)_3_**6** (3.69 g, 1.78 mmol) in MeCN (20 mL) and the solution was heated at 110 °C for 23 h. The crystalline solid formed upon cooling to room temperature was filtered off, the filtrate evaporated and the residue washed with diethyl ether (3 × 25 mL) and dried under vacuum. Single crystals formed in an NMR solution in MeCN were used for an X-ray crystal structure determination that revealed the presence of both anions **6** and **10**. Recrystallization from MeCN-THF gave a crystalline solid (2.38 g) that was shown by ^119^Sn NMR to contain **10** (82%) and **6** (18%). A further treatment with DCC (0.51 g, 2.47 mmol) in MeCN (10 mL) gave a solid after workup that did not contain **6**. Yield 2.20 g, 60%.

### (TBA)_8_[(μ-O)(TiPW_11_O_39_)_2_] (TBA)_8_**11**

H_2_O (2 mL, 111 mmol) was added to a solution of (TBA)_4_**3** (5.20 g, 1.40 mmol) in MeCN (40 mL) and the volatiles were removed after stirring for 3 h. The hydrolysis was repeated three more times and the white solid remaining after removal of the solvent was dissolved in MeCN (30 mL) followed by addition of activated 3A molecular sieves. After 2 days, the solution was filtered and vacuum-dried for 4 h to give a white solid (4.80 g, 99%). In a separate experiment, the TiOTi bridging oxygen was selectively enriched by hydrolysis of nonenriched (TBA)_4_**7** with ∼0.1% ^17^O-enriched H_2_O.

### (TBA)_8_[(μ-O)(SnPW_11_O_39_)_2_] (TBA)_8_**12**

*N*,*N*′-dicyclohexylcarbodiimide (DCC) (0.21 g, 0.99 mmol, 14 equiv) was added to (TBA)_4_**8** (0.54 g, 0.14 mmol) in MeCN (10 mL) in a sealed flask. The solution was heated at ∼110 °C for 48 h then allowed to cool to room temperature, whereupon colorless crystals formed. The solution was then filtered, vacuum-dried, washed with THF (15 mL × 6) and dried again to give a white solid (0.28 g, 52%).

NMR data (Tables S1–S5) and IR spectra for all compounds are provided in the Supporting Information.

## Results

Methoxido-derivatized, titanium- and tin-substituted Lindqvist-type hexametalates (TBA)_3_**1** and (TBA)_3_**2** respectively were synthesized by published nonaqueous procedures.^7,9^ The titanium- and tin-substituted Keggin-type methoxides (TBA)_4_**3** and (TBA)_4_**4** respectively were obtained from the chlorides (TBA)_4_[ClMPW_11_O_39_] (M = Ti or Sn), which were prepared by treating (TBA)_6_[NaPW_11_O_39_] with TiCl_4_ or SnCl_4_ respectively in CH_2_Cl_2_.^24^ The tin analogue (TBA)_4_**4** is considerably more moisture-sensitive than (TBA)_4_**3**, and trace hydrolysis was only circumvented by treatment of the initial product from chloride metathesis with an excess of MeOH. NMR data for Lindqvist and Keggin anions described below are collected in Tables S1–S5.

### Methanolysis

NMR *T*_1_ relaxation times for MOCH_3_ protons were measured for samples prepared under nitrogen and were found to vary from 2.79 to 10.79 s as shown in Table S6. Longer relaxation times are associated with slow correlation times for these anions in solution and this was taken into account by using suitable acquisition delay times in NMR kinetic experiments requiring integration of these peaks. The anions **1–4** undergo alkoxide exchange at the six-coordinate heterometal centers upon treatment with primary, secondary or tertiary alcohols,^11,20^ and we observed that alcoholysis occurred more readily at the tin heterometal center in (TBA)_4_**2** than at titanium in (TBA)_3_**1**. The rates of methanol–methoxido exchange for anions **1–4** were compared in a series of 2D ^1^H NMR EXSY experiments. Off-diagonal SnOMe/MeOH exchange peaks were observed for the tin-substituted (TBA)_3_**2** and (TBA)_4_**4** with exchange rates (pseudo-first order rate constants) of 0.08 and 0.03 s^–1^, respectively, but no exchange peaks were observed for titanium-substituted (TBA)_3_**1** and (TBA)_4_**3** (Figure S1), even at 60 °C or after addition of H_2_O, indicating a maximum exchange rate at Ti of 0.02 s^–1^.
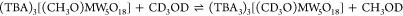
1
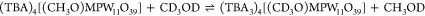
2

**Figure 2:**
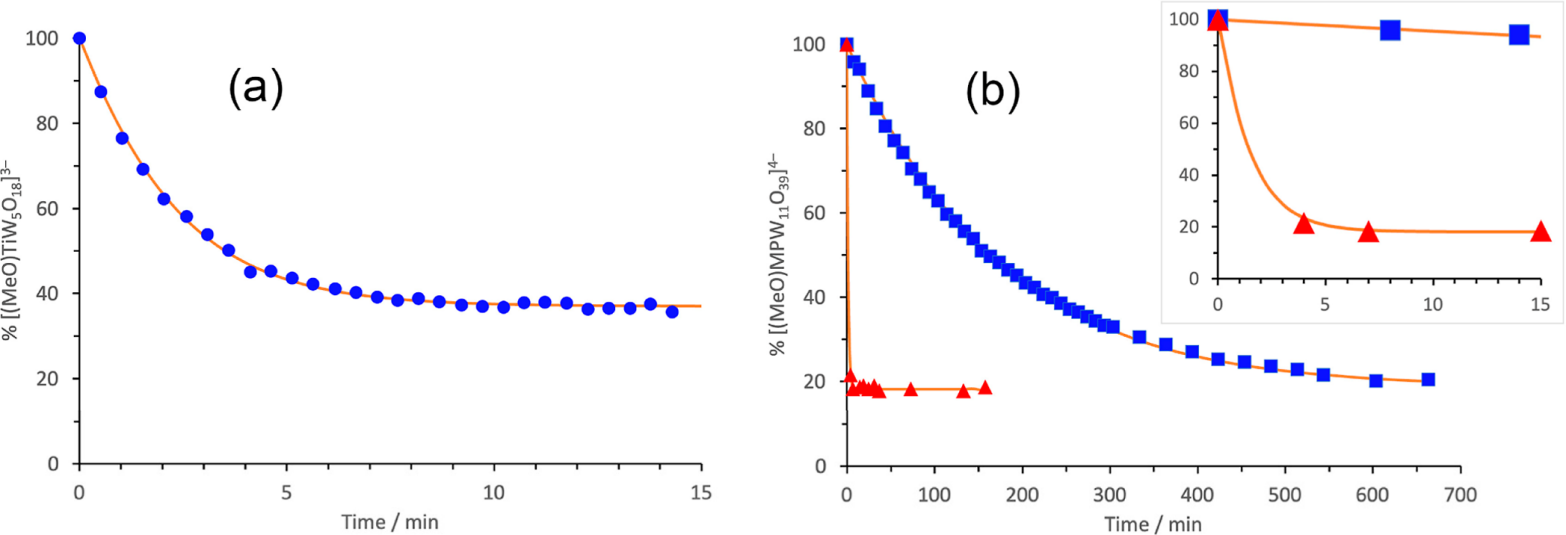
^1^H NMR kinetic plots for MOMe/CD_3_OD exchange in CD_3_CN for (a) (TBA)_3_[(MeO)TiW_5_O_18_] (TBA)_3_**1**; [TiW_5_]_0_ = 0.05 M, [CD_3_OD]_0_ = 0.20 M, 298 K and (b) (TBA)_4_[(MeO)TiPW_11_O_39_] (TBA)_4_**3** (blue ■) and (TBA)_4_[(MeO)SnPW_11_O_39_] (TBA)_4_**4** (red ▲); [MPW_11_]_0_ = 0.02 M, [CD_3_OD]_0_ = 0.19 M, 295 K. Inset is an expansion of the initial 15 min and lines show a least-squares fit of the kinetic model of eq 1 (see Supporting Information).

To gain further insight into the alcoholysis reactions, we monitored the decrease in intensity of the MOCH_3_ peak in ^1^H NMR spectra upon addition of dry CD_3_OD to the methoxido anions **1**, **3** and **4** (eqs 1 and 2). The plots in Figure 2 show that exchange for Keggin-type {TiPW_11_} **3** is remarkably slower than Lindqvist-type {TiW_5_} **1** and that exchange for the tin-substituted {SnPW_11_} anion **4** was much faster than for the Ti homologue **3**. Data for {SnW_5_} **2** were unreliable due to the rapid rate of exchange and inherent uncertainties in peak integrations and are not included.

### Hydrolysis

It became apparent during the course of this work that, while all of the methoxido POMs are somewhat moisture-sensitive (eqs 3 and 4), the tin derivatives (TBA)_3_**2** and (TBA)_4_**4** are much more readily hydrolyzed than their titanium analogues (TBA)_3_**1** and (TBA)_4_**3**, and isolation of pure tin methoxido compounds required extra care.
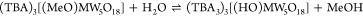
3
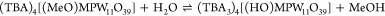
4

**Figure 3:**
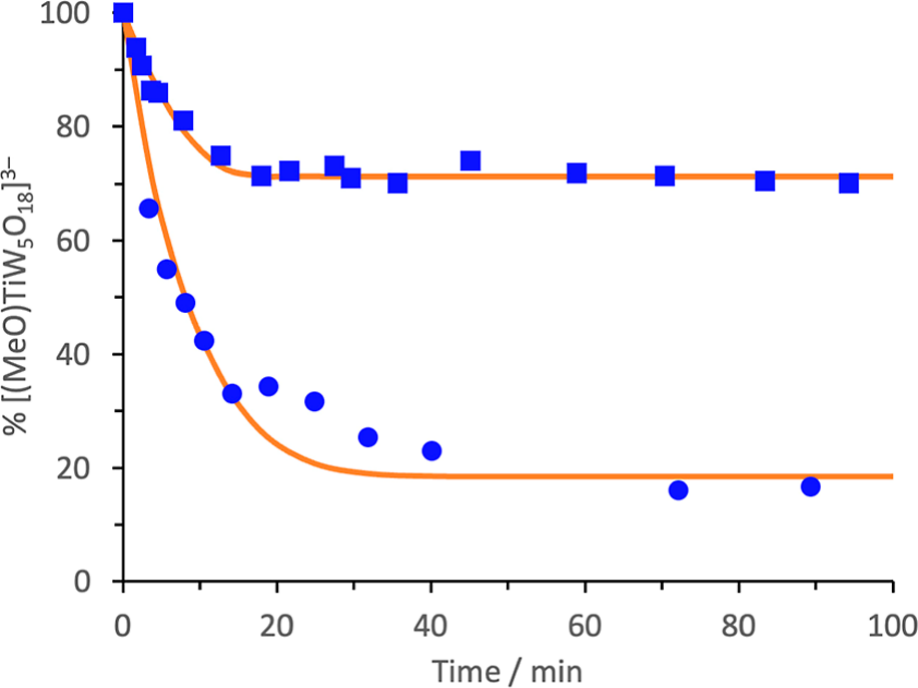
^1^H NMR kinetic plots for hydrolysis of (TBA)_3_[(MeO)TiW_5_O_18_] (TBA)_3_**1** in CD_3_CN with 2 (blue ■) and 44 (blue ●) mole-equivalents of H_2_O. Solid lines show the least-squares fit (see Supporting Information).

**Figure 4:**
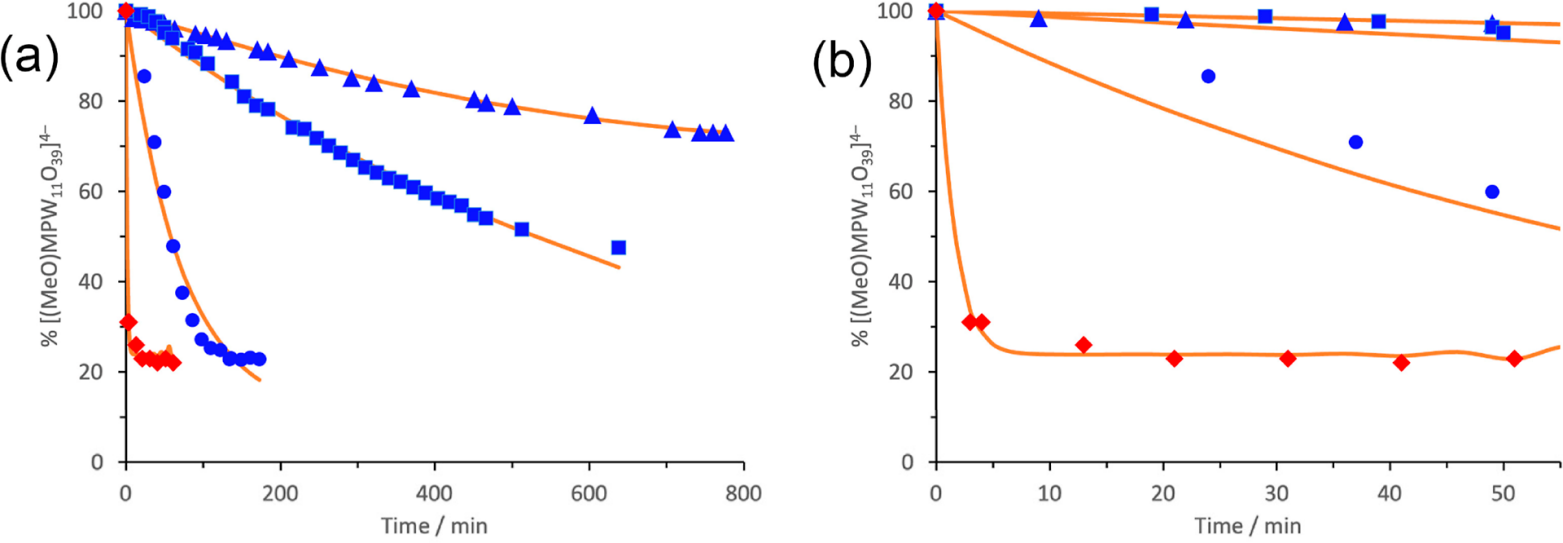
^1^H (CD_3_CN) and ^31^P (DMSO) NMR kinetic plots for hydrolysis of (TBA)_4_[(MeO)TiPW_11_O_39_] (TBA)_4_**3** in CD_3_CN with 10 (blue ▲) and 50 (blue ■) mole-equivalents of H_2_O and in DMSO with 10 mol-equivalents of H_2_O (blue ●), and for hydrolysis of (TBA)_4_[(MeO)SnPW_11_O_39_] (TBA)_4_**4** in CD_3_CN with 10 mol-equivalents of H_2_O (red ◇), showing reaction times up to 800 (a) and 55 (b) minutes. Solid lines show the least-squares fit (see Supporting Information).

Our qualitative observations were confirmed by monitoring MOMe peaks in ^1^H NMR spectra upon addition of water, which showed incomplete conversion of (TBA)_3_**1** to hydrolysis products in CD_3_CN over several hours, whereas the SnOMe peak for (TBA)_3_**2** at 3.65 ppm in CD_3_CN had almost disappeared in the short time taken to insert the sample into the probe and record the first spectrum (Figure S2). Kinetic plots from ^1^H NMR kinetic studies of (TBA)_3_**1** hydrolysis in CD_3_CN are shown in Figure 3, and analogous plots were obtained in (CD_3_)_2_SO. As with CD_3_OD exchange, data for {SnW_5_} **2** are not included because of the rapid rate of reaction and inherent limitations of peak integrations. Hydrolysis of Keggin species (TBA)_3_**3** and (TBA)_4_**4** in MeCN was monitored by ^31^P NMR spectroscopy, whereupon the peak for **3** at −14.05 ppm (−14.26 ppm in DMSO) was replaced by the peak for **7** at −14.14 ppm (−14.32 ppm in DMSO), and the peak for **4** at −12.66 ppm by that for **8** at −12.60 ppm (Figure S3). Kinetic plots from ^1^H NMR hydrolysis data shown in Figure 4 demonstrate the effects of the nature of the solvent as well as water concentration on these proton-transfer reactions. Subsequent condensation to oxido-bridged [(μ-O)(MPW_11_O_39_)_2_]^8–^ was only observed for M = Ti, where a ^31^P NMR peak for **11** was observed at −14.08 ppm. Facile SnOMe hydrolysis was also evident in ^119^Sn{^1^H} NMR spectra, where the singlet for **2** at −648 ppm and the doublet for **4** at −622 ppm were replaced by resonances at −633 and −600 ppm for **6** and **8** respectively (Figure S4).

### Lindqvist {HOMW_5_} Hydroxido Derivatives

#### (TBA)_3_[(HO)TiW_5_O_18_] (TBA)_3_**5**

**Figure 5:**
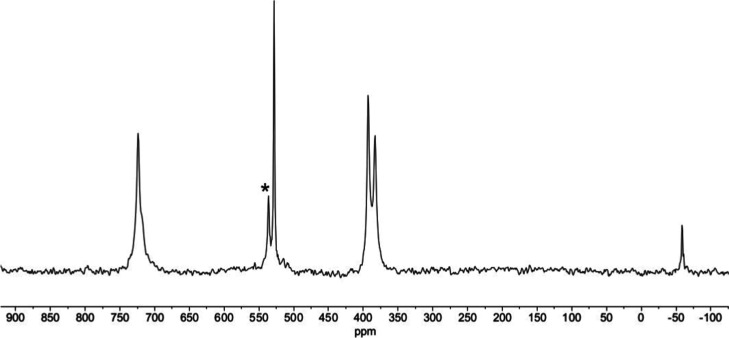
^17^O NMR spectrum of (TBA)_3_[(HO)TiW_5_O_18_]^3–^ (TBA)_3_**5**. The peak marked with an asterisk is due to (TBA)_6_[(μ-O)(TiW_5_O_18_)_2_] (TBA)_6_**9**.

Our previous attempts to isolate (TBA)_3_**5**, i.e. the hydroxido species expected from the hydrolysis of (TBA)_3_**1** as in eq 3, were hampered by incomplete hydrolysis at ambient temperatures and the propensity for monomeric **5** to undergo condensation to oxido-bridged **9** at elevated temperatures.^11^ A strategy was therefore required that promoted hydrolysis and minimized condensation. Initial efforts involved repeated hydrolysis in MeCN at ambient temperature (20 °C) with removal of the volatiles under reduced pressure after each hydrolysis step *without heating*. This gave a product which did indeed show ν(OH) at 3676 cm^–1^ in the ATR FTIR spectrum, a value close to the band observed at 3684 cm^–1^ in the IR spectrum of the titanium (IV) “atrane” complex LTiOH, where L is the trianionic ligand derived from tris(2-hydroxy-3,5-di-*tert*-butylbenzyl)amine.^35^ Although consistent with the formation of **5**, an additional IR band at 673 cm^–1^ due to ν_as_(TiOTi) of **9** indicated that some condensation had occurred, even under these mild conditions. However, we found that condensation was inhibited in DMSO, which enabled the isolation of (TBA)_3_**5** via repeated cycles of mild hydrolysis in DMSO followed by precipitation with diethyl ether. The FTIR spectrum of the product contained ν(OH) but no strong band due to ν_as_(TiOTi) and, upon deuteration, a weak band appeared at 2707 cm^–1^ for ν(OD) (Figure S5). A singlet at 9.57 ppm in the ^1^H NMR spectrum of (TBA)_3_**5** in (CD_3_)_2_SO (Figure S6) disappeared upon treatment with D_2_O and was therefore assigned to TiOH. The ^17^O NMR spectrum of (TBA)_3_**5** obtained by treating ^17^O-enriched (TBA)_3_**1** in DMSO with nonenriched H_2_O at room temperature is shown in Figure 5 and is characteristic of *C*_4v_ [XTiW_5_O_18_]^3–^ anions.^7,11^ This hydrolysis procedure inevitably reduces the amount of ^17^O in the {TiW_5_O_18_} framework through exchange with the excess of nonenriched H_2_O, but sufficient enrichment remained to obtain a spectrum. The peak at 722 ppm is due to terminal W_(eq)_=O with a shoulder at 716 ppm for W_(ax)_=O, while TiOW bridging oxygens appear at 526, WOW at 391 and 381 and central μ_6_-O at −56 ppm. The minor TiOW peak at 534 ppm is due to a small amount of condensation product (TBA)_6_**9**. The ^17^O NMR spectrum of the solid obtained after stirring non-^17^O-enriched (TBA)_3_**5** with an excess of ^17^O-enriched water in DMSO for 2 h and precipitation with diethyl ether contained only two peaks immediately after dissolution in CD_3_CN (Figure S7). The peak at 681 ppm is very close to that previously observed for TiOTi of condensation product **9** (679 ppm), suggesting that the peak at 14 ppm may be assigned to TiOH. However, this is not without ambiguity as solvent-dependent chemical shifts for DMSO are in the same range,^36^ although trace DMSO carried over in the precipitate is not expected to be ^17^O-enriched during the hydrolysis reaction. Over the course of several days the TiOTi peak intensity increases and peaks due to TiOW and WOW appear in this spectrum. This may be explained by condensation of TiOH to TiOTi with subsequent exchange of ^17^O from the resulting H_2_^17^O into the POM framework.^37^

**Figure 6:**
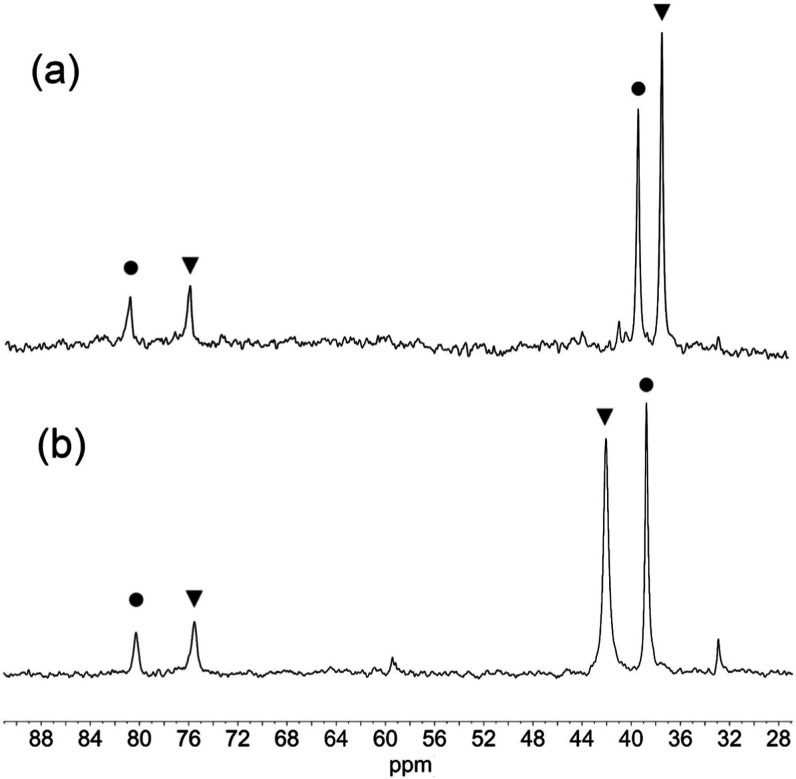
^183^W NMR spectra of equilibrium mixtures of (TBA)_3_[(HO)TiW_5_O_18_] (TBA)_3_**5** (▼) and (TBA)_6_[(μ-O)(TiW_5_O_18_)_2_] (TBA)_6_**9** (**•**) in (a) MeCN and (b) DMSO.

Although condensation of (TBA)_3_**5** is slowed sufficiently in DMSO to enable its isolation, significant amounts of (TBA)_6_**9** are formed over extended periods in this solvent, as was evident from ^183^W NMR spectra acquired over several days (Figure 6). Peaks in these spectra associated with oxido-bridged (TBA)_6_**9** were assigned on the basis of the chemical shift difference between W_eq_ and W_ax_ (41.6 ppm) as observed previously.^11^ The chemical shift of the W_eq_ peak for (TBA)_3_**5** is particularly solvent-sensitive, appearing at 37.4 ppm in MeCN and 42.0 ppm in DMSO, and the peak is also notably broadened in DMSO. Crystals of (TBA)_3_**5** were grown by vapor diffusion of Et_2_O into a mixture of MeCN and DMSO and used for a single-crystal X-ray structure determination (Table S7), which confirmed the monomeric nature of the anion. (TBA)_6_**9** formed during the diffusion period was presumably more soluble and remained in solution while (TBA)_3_**5** crystallized. Data refinement showed two independent anions in which the {TiOH} group was disordered equally over two trans positions in one anion (Figure S8) and equally over all six metal positions in the other. Although the disorder prevented a full analysis of bond lengths and angles, selected data are given for the trans-disordered anion of (TBA)_3_**5** in Table S8.

#### (TBA)_3_[(HO)SnW_5_O_18_] (TBA)_3_**6**

**Figure 7:**
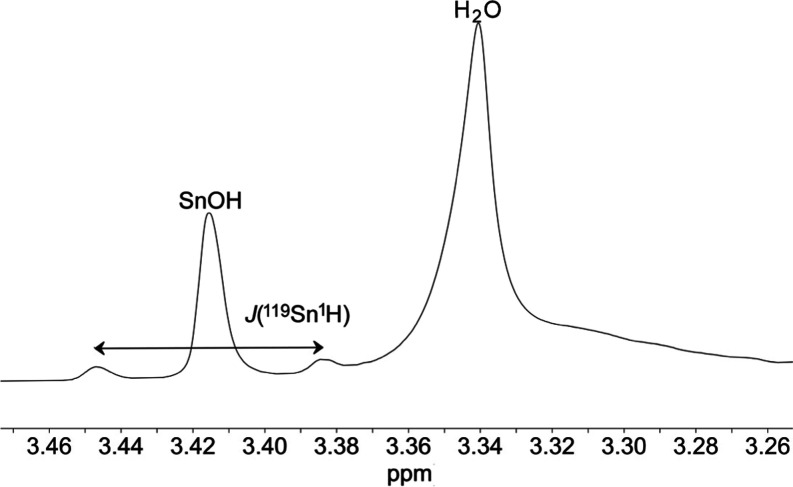
SnO*H* resonance in the ^1^H NMR spectrum of (TBA)_3_[(HO)SnW_5_O_18_] (TBA)_3_**6** in (CD_3_)_2_SO.

**Figure 8:**
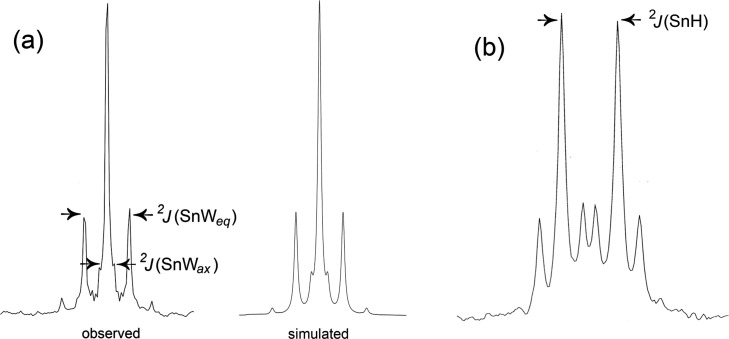
Observed and simulated ^119^Sn{^1^H} NMR resonance (a) and observed ^119^Sn NMR resonance (b) for (TBA)_3_[(HO)SnW_5_O_18_] (TBA)_3_**6**.

In contrast to the titanium system, the tin hydroxido analogue (TBA)_3_**6** was obtained readily from (TBA)_3_**2** by hydrolysis with an excess of water at room temperature and precipitation with diethyl ether. SnOMe hydrolysis is much faster than TiOMe hydrolysis, while condensation of **6** is less favorable than that of **5**. The formation of (TBA)_6_**10** was also inhibited in DMSO or PhCN at ambient temperatures. In the 700 MHz ^1^H NMR spectrum of (TBA)_3_**6** in (CD_3_)_2_SO, a broadened resonance at 3.42 ppm with ^119/117^Sn satellites and ^2^*J*(^119/117^Sn^1^H) of ca. 44 Hz disappeared after addition of D_2_O and was therefore assigned to SnOH (Figure 7). A weak band for ν(OH) in the ATR FTIR spectrum of (TBA)_3_**6** was observed at 3648 cm^–1^ in addition to the characteristic peaks for ν(W=O) at 951, ν(W–OW) at 784 and ν(Sn–OW) at 748 cm^–1^, while in the spectrum of the deuterated analogue ν(OD) was observed at 2688 cm^–1^ (Figure S9). In the ^119^Sn{^1^H} NMR spectrum of (TBA)_3_**6**, the satellite peaks for the resonance at −633 ppm showed ^2^*J*(^119^Sn^183^W_eq_) = 37 Hz and ^2^*J*(^119^Sn^183^W_ax_) = 13 Hz, while a further doublet coupling due to ^2^*J*(^119^Sn^1^H) = 48 Hz was observed in the absence of ^1^H decoupling, thereby confirming the presence of SnOH (Figure 8).

**Figure 9:**
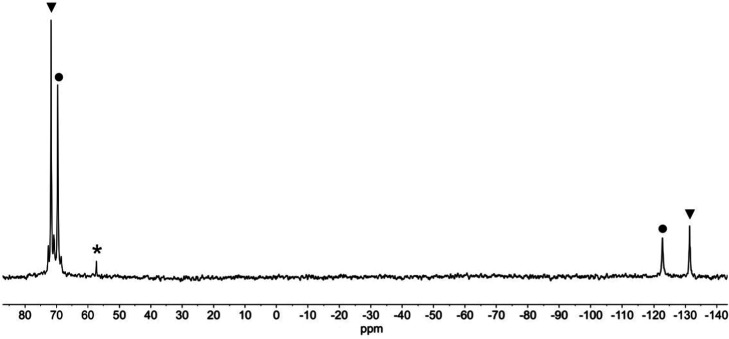
^183^W NMR spectrum of a mixture of (TBA)_3_[(HO)SnW_5_O_18_] (TBA)_3_**6** (▼) and (TBA)_6_[(μ-O)(SnW_5_O_18_)_2_] (TBA)_6_**10** (•) in CH_3_CN/CD_3_CN. The peak marked with an asterisk is due to (TBA)_2_[W_6_O_19_].

A ^183^W NMR spectrum of (TBA)_3_**6** recorded over several days (Figure 9) showed peaks for oxido-bridged **10** in addition to **6** due to condensation but, following the synthesis of (TBA)_6_**10**, the peaks at 72.7 ppm with ^2^*J*(^119^Sn^183^W_eq_) = 37 Hz and at −130.2 ppm with unresolved ^2^*J*(^119^Sn^183^W_ax_) could be unambiguously assigned to **6**. The 4:1 ratio of W_eq_ to W_ax_ peaks is consistent with *C*_4*v*_ symmetry and terminal SnOH in **6**. A sample of (TBA)_3_**6** obtained by hydrolysis of ^17^O-enriched (TBA)_3_**2** with nonenriched H_2_O gave an ^17^O NMR spectrum that was similar to that of (TBA)_3_**2**, with peaks for W_eq_=O at 722, W_ax_=O at 685, SnOW at 397, WOW at 384 and 369, and μ_6_-O at 18 ppm (Figure S10). In an attempt to identify a peak for SnOH in the ^17^O NMR spectrum, a dried sample of (TBA)_3_**6** was treated with ^17^O-enriched H_2_O, but the new peak observed at −5 ppm is in the region expected for H_2_O, suggesting that this may be due to exchange between SnOH and H_2_O. As was the case for **5**, an unambiguous determination of δ_O_ for terminal OH was therefore not possible. A single-crystal X-ray crystal structure determination confirmed the presence of monomeric anions in (TBA)_3_**6**, but disorder of the SnOH group over all six apical positions prevented any meaningful analysis of bond lengths and angles and data are not included here. Fortuitously, however, a nondisordered anion was present in cocrystalline (TBA)_9_[(μ-O)(SnW_5_O_18_)_2_]·[(HO)SnW_5_O_18_]·3MeCN obtained from a synthesis of (TBA)_6_**10** and the structure is discussed below.

### Keggin {HOMPW_11_} Hydroxido Derivatives

#### (TBA)_4_[(HO)TiPW_11_O_39_] (TBA)_4_**7**

**Figure 10:**
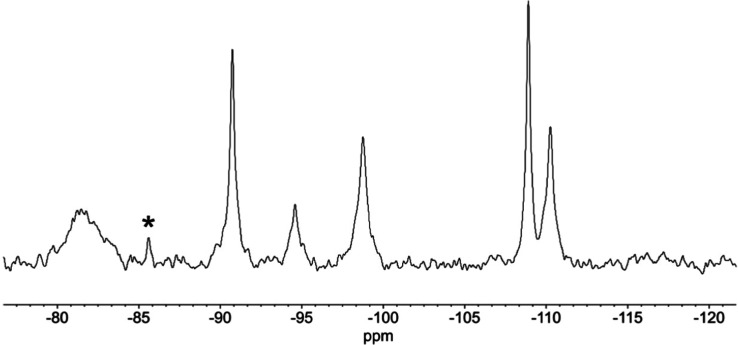
^183^W NMR spectrum of (TBA)_4_[(HO)TiPW_11_O_39_] (TBA)_4_**7** in DMSO. The peak marked with an asterisk is assigned to [PW_12_O_40_]^3–^.

Repeated mild hydrolysis of the {(MeO)TiPW_11_} Keggin derivative (TBA)_4_**3** with a 2000-fold excess of water for ca. 1 h gave (TBA)_4_**7** in 91% yield after six hydrolysis cycles, as shown by the major peak at −14.14 ppm in the ^31^P NMR spectrum (δ_P_ for **3** is −14.05 ppm). Despite the use of a large excess of water, a minor ^31^P NMR peak at −14.08 ppm indicated the presence of oxido-bridged (TBA)_8_**11**. Following our experience with the Lindqvist anion **1**, pure (TBA)_4_**7** was therefore prepared by a single treatment of (TBA)_4_**3** with a 200-fold excess of water in DMSO to suppress condensation. In the FTIR spectrum of the isolated solid, ν(OH) was present at 3633 cm^–1^ (Figure S11), in addition to bands for ν(PO) at 1070 cm^–1^, ν(W=O) at 960 cm^–1^ and ν(WOW) at 791 cm^–1^. The ^1^H NMR spectrum of (TBA)_4_**7** in DMSO-*d*_6_ (Figure S12) contains a peak for TiOH at 12.04 ppm which is absent after addition of D_2_O. A sample of (TBA)_4_**7** obtained from ^17^O-enriched (TBA)_4_**3** and nonenriched H_2_O gave ^17^O NMR peaks between 739 and 736 ppm for W=O, at 559 and 536 ppm for TiOW and between 424 and 401 ppm for WOW as shown in Figure S13 but, as in the case of the Lindqvist anions **5** and **6** we were unable to assign a peak to terminal OH in spectra of samples obtained with ^17^O-enriched H_2_O. The ^183^W NMR spectrum of (TBA)_4_**7** in DMSO (Figure 10) showed six peaks in the ratio 2:2:1:2:2:2, consistent with the *C*_*s*_ symmetry of a monosubstituted α-Keggin anion containing terminal {TiOH}^3+^. The most deshielded peak at −81.5 ppm is notably broader than the others (fwhm = 45 Hz), perhaps suggesting a dynamic process that only affects the environments of these tungstens. On the basis of previous ^183^W NMR studies of {XTiPW_11_} Keggin anions,^17,23^ this peak is assigned to the tungsten atoms within the TiW_2_ triad, which contains basic TiOW sites that might engage in intermolecular H-bonding and proton exchange involving TiOH. The DMSO solvent apparently inhibits the elimination of H_2_O that might otherwise result from such proton dynamics, whereas a ^183^W NMR spectrum of **7** in MeCN could not be obtained because of condensation to **11** during spectrum acquisition.

#### (TBA)_4_[(HO)SnPW_11_O_39_] (TBA)_4_**8**

**Figure 11:**
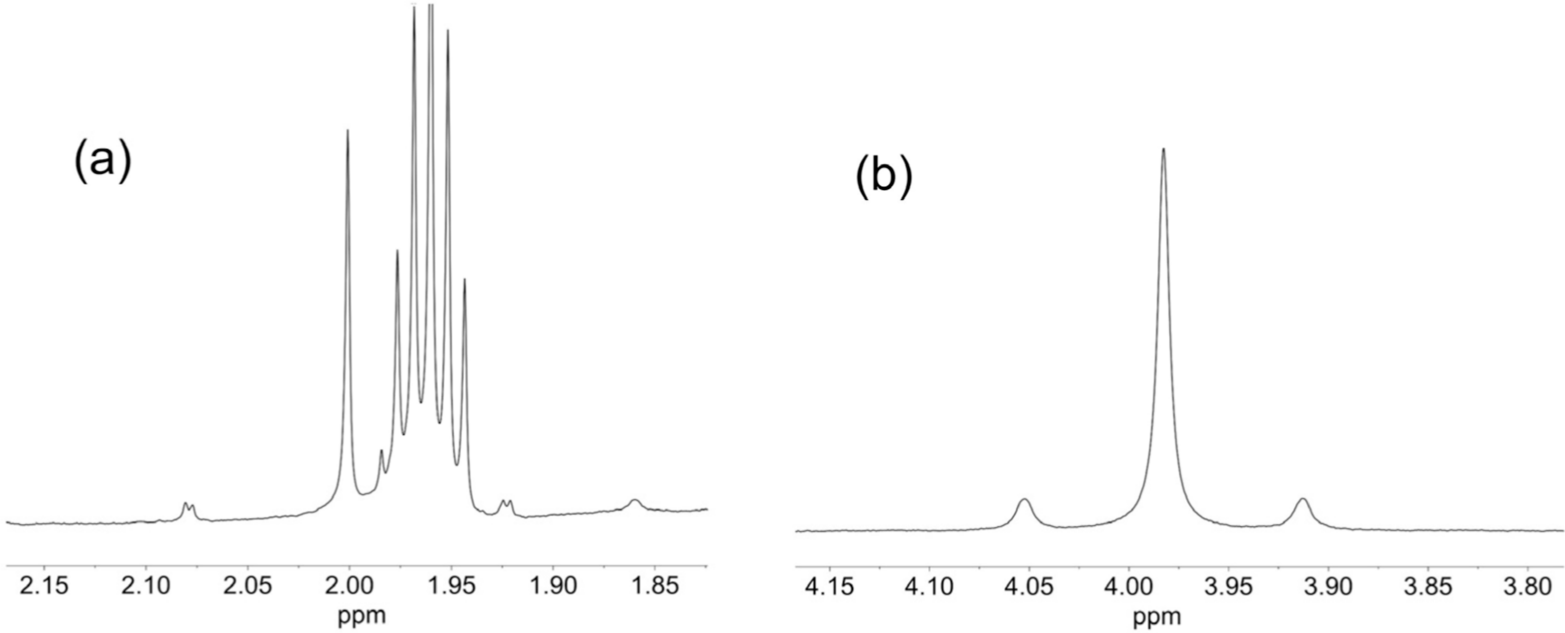
^1^H NMR spectra of (TBA)_4_[(HO)SnPW_11_O_39_] (TBA)_4_**8** in MeCN (a) and (CD_3_)_2_SO (b) showing the SnOH resonance.

The {HOSnPW_11_} Keggin derivative was readily obtained by hydrolysis of the methoxido species (TBA)_4_**4** in MeCN at room temperature. A weak band for ν(OH) was present at 3639 cm^–1^ in the FTIR spectrum of the isolated solid, and the corresponding ν(OD) appeared at 2684 cm^–1^ after deuteration (Figure S14). Interestingly, two bands for ν(PO) are present at 1077 and 1058, rather than the single band observed for (TBA)_4_**7** at 1070 cm^–1^ together with ν(W=O) at 961 and ν(SnOW/WOW) at 795 cm^–1^. In CD_3_CN the ^1^H NMR peak for SnOH at 2.00 ppm with ^2^*J*(^1^H^119^Sn) = 48 Hz and ^2^*J*(^1^H^117^Sn) = 46 Hz was close to the C*H*D_2_CN peaks, whereas in (CD_3_)_2_SO the SnOH resonance at 3.98 ppm with ^2^*J*(^1^H^119^Sn) = 42 Hz was well separated from the residual C*H*D_2_ solvent signal at 2.50 ppm (Figure 11).

**Figure 12:**
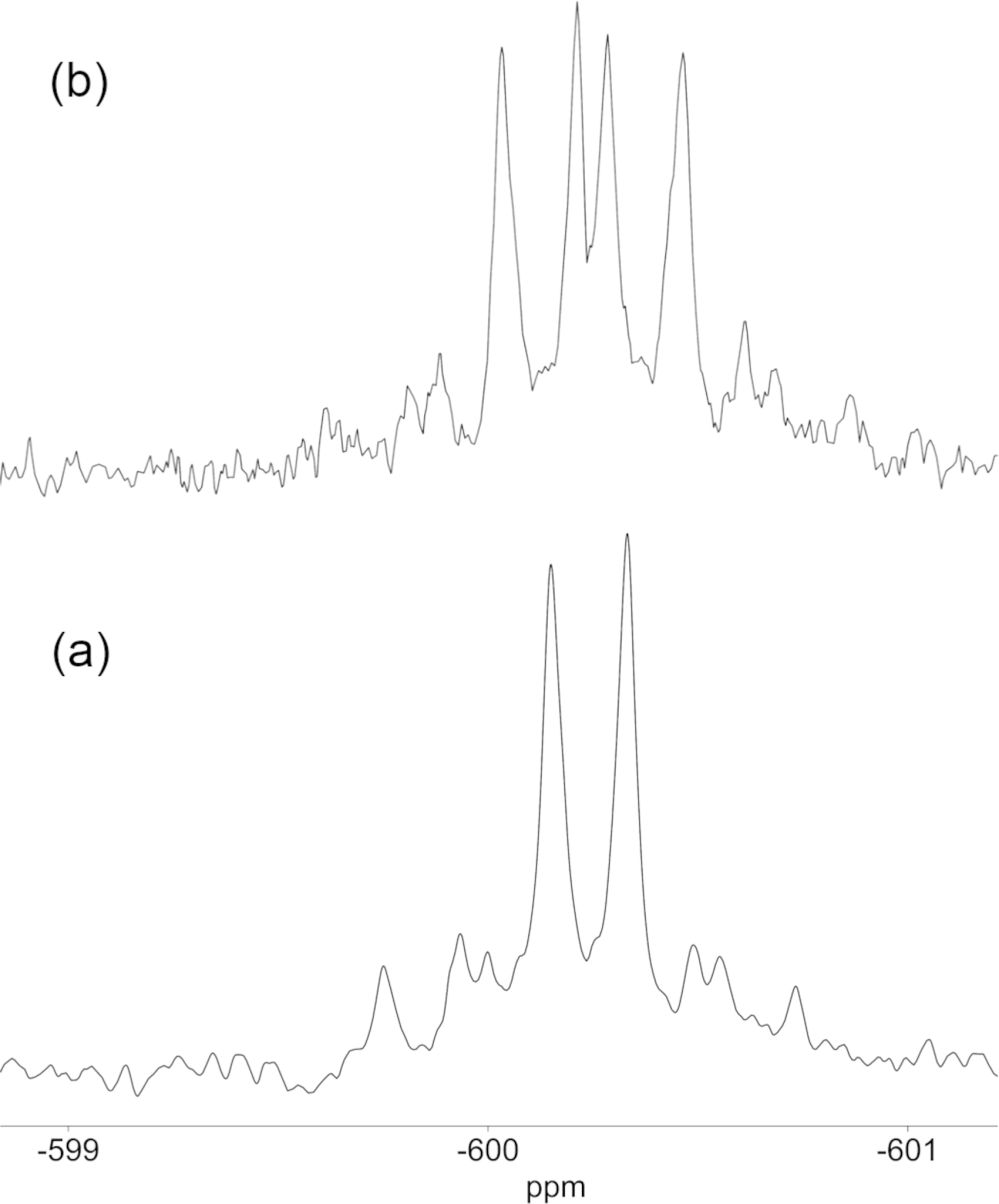
^119^Sn{^1^H} (a) and ^119^Sn (b) NMR spectra of (TBA)_4_[(HO)SnPW_11_O_39_] (TBA)_4_**8**.

**Figure 13:**
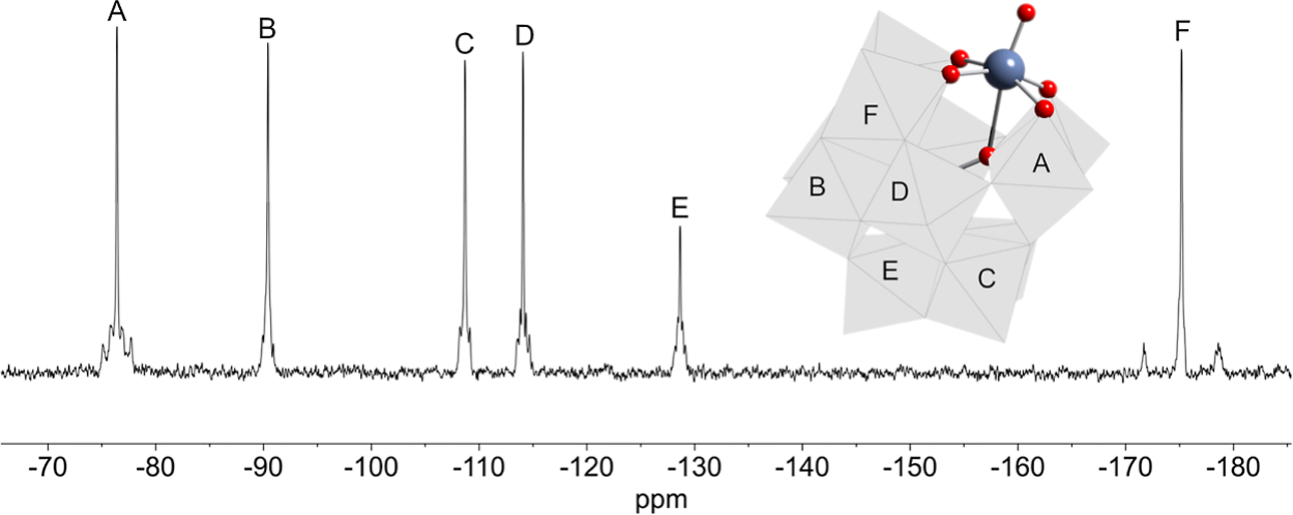
^183^W NMR spectrum of (TBA)_4_[(HO)SnPW_11_O_39_] (TBA)_4_**8**.

The ^119^Sn NMR spectrum of **8** showed ^2^*J*(^31^P^119^Sn) = 34 Hz and ^2^*J*(^1^H^119^Sn) = 47 Hz, and ^1^H decoupling further confirmed the presence of SnOH (Figure 12). Satellite peaks were also observed due to ^2^*J*(^183^W^119^Sn) of 75 Hz. The ^17^O NMR spectrum of (TBA)_6_**8** obtained from ^17^O-enriched (TBA)_6_**4** and nonenriched H_2_O is shown in Figure S15a. Peaks for W=O appear between 746 and 733 while those for SnOW and WOW are grouped in the region 427–340 ppm. Figure S15b shows the ^17^O NMR spectrum of the dried product from hydrolysis of nonenriched (TBA)_4_**4** with ^17^O-enriched H_2_O, in which the single peak at −5.7 ppm (fwhm ca. 230 Hz) is in the region expected for H_2_O, although the line width could indicate rapid exchange between SnOH and H_2_O, preventing unambiguous assignment of δ(OH). In the ^183^W NMR spectrum of (TBA)_6_**8** shown in Figure 13, ^2^*J*(^183^W^119^Sn) couplings of 54 and 144 Hz for peaks A and F enable these to be assigned together with peak E as shown on the polyhedral structural diagram. More tentative assignments of peaks B, C and D are made on the basis of the ^2^*J*(^183^W^183^W) satellite couplings of ∼20 and ∼10 Hz associated with larger and smaller WOW angles, respectively.

### Condensation of Lindqvist {(HO)MW_5_} Anions

The availability of hydroxido Lindqvist anions **5** and **6** enabled condensation reactions to be studied by NMR spectroscopy. The titanium oxido-bridged (TBA)_6_**9** was most conveniently prepared from methoxide (TBA)_3_**1** by hydrolysis at 85–90 °C as described previously,^11^ and also formed readily in solutions of the isolated hydroxide (TBA)_3_**5** in MeCN under ambient conditions. Even in DMSO, when condensation was suppressed, peaks for **9** appeared in the ^183^W NMR spectrum of (TBA)_3_**5** (Figure 6) due to the long acquisition times required, as discussed above. The ^119^Sn NMR spectrum of the hydroxide (TBA)_3_**6** in MeCN contained an extra minor resonance at −667 ppm due to oxido-bridged (TBA)_6_**10** and this peak grew in intensity over several days at ambient temperature. Assuming that these reactions are equilibria, approximate equilibrium constants *K*_5_^Ti^ = 0.14 and *K*_5_^Sn^ = 0.025 for eq 5 were derived from ^183^W and ^119^Sn NMR spectra respectively (Figures S16 and S17), although it is possible that equilibrium had not been fully attained.
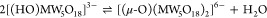
5

**Figure 14:**
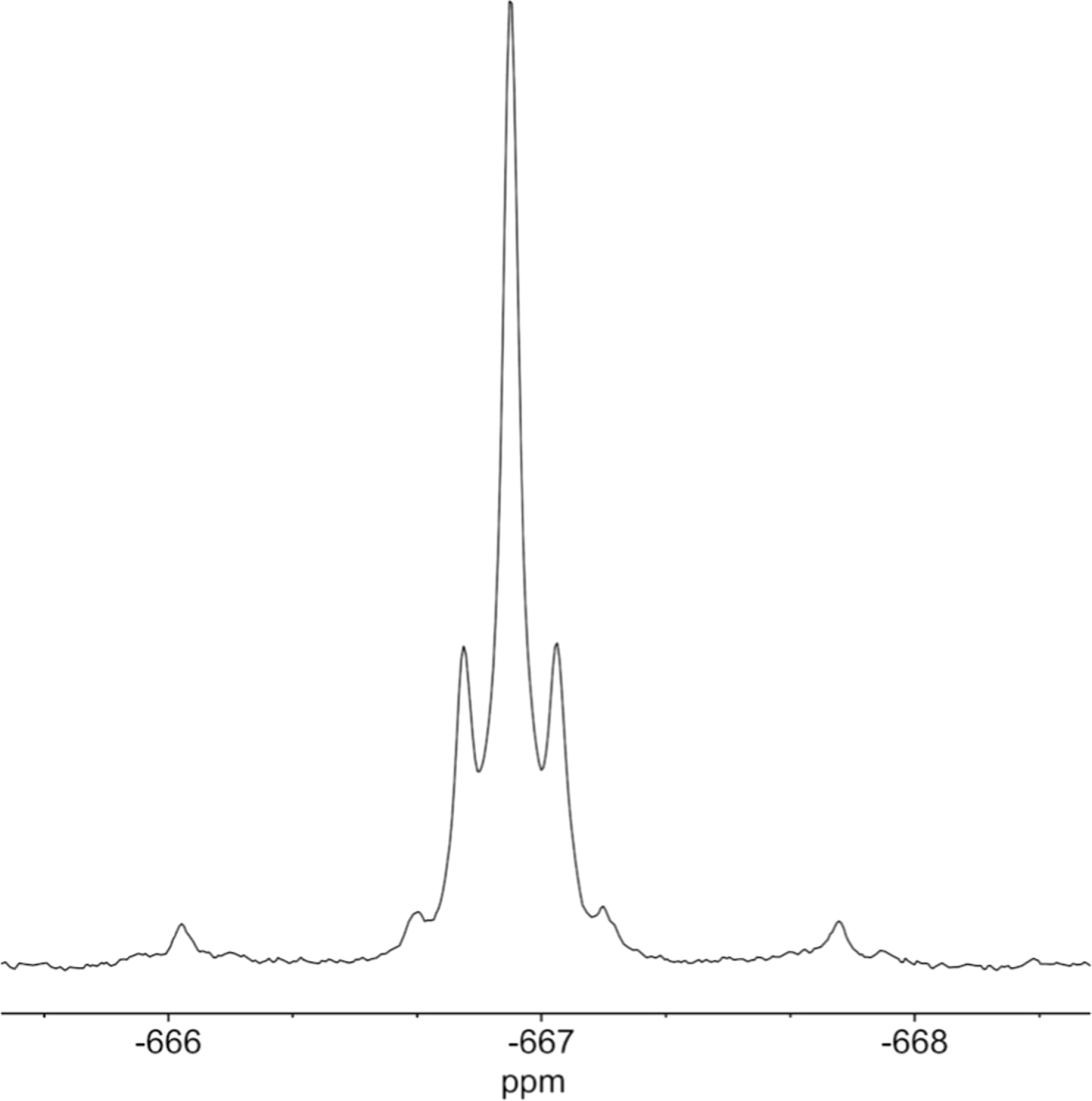
^119^Sn NMR spectrum of (TBA)_6_[(μ-O)(SnW_5_O_18_)_2_] (TBA)_6_**10**.

In contrast to the homologous titanium system, complete conversion of **6–10** proved to be rather difficult. Initial attempts involved periodic removal of volatiles while heating a solution of (TBA)_3_**6** in PhCN at 110 °C under reduced pressure. This process was monitored by ^119^Sn NMR and continued until complete conversion to (TBA)_6_**10** (Figure S18). More conveniently, condensation could be driven to completion by heating with an excess of *N*,*N*′-dicyclohexyl carbodiimide (DCC) in MeCN, although the product very easily reverted to **6** by reacting with trace amounts of H_2_O. In the ^119^Sn spectrum of **10** recorded without ^1^H decoupling (Figure 14) the presence of satellites with ^2^*J*(^119^Sn^117^Sn) = 333 Hz and the absence of ^2^*J*(^1^H^119^Sn) confirmed the formation of a SnOSn linkage. Peaks in the ^17^O NMR spectrum of **10** are significantly broader than those for **6** presumably due to a longer correlation time and greater molecular anisotropy (Figure S19). Peaks at 720 and 681 ppm are due to W=O, while peaks at 396 and 384 ppm are tentatively assigned to SnOW and overlapping WOW respectively. The central μ_6_-O appears at 18 ppm, but a peak for SnOSn could not be unambiguously identified. In the ^183^W NMR spectrum of a mixture of **6** and **10** shown in Figure 9, the peak for W_eq_ in **10** at 69.6 ppm shows satellites with ^2^*J*(^119^Sn^183^W_eq_) = 46 Hz, but ^2^*J*(^119^Sn^183^W_ax_) coupling is not resolved for the W_ax_ peak at −122.8 ppm. A band at 628 cm^–1^ in the ATR FTIR spectrum of (TBA)_6_**10** was assigned to ν_as_(SnO) of the SnOSn bridge, while bands assigned to ν(W–OW) at 796 cm^–1^ and ν(Sn–OW) at 719 cm^–1^ are higher and lower respectively than those for (TBA)_3_**6** (Figure S20). However, the assignments for ν(Sn–OW) and ν(SnOSn) are somewhat ambiguous, and it is possible that the band at 719 cm^–1^ is due to ν(SnOSn). The X-ray structure of a crystal obtained from an NMR solution of the product after treatment of (TBA)_3_**6** with DCC confirmed the presence of **10** and, rather fortuitously, a nondisordered anion of **6**. The anion structures are shown in Figure S21 and selected bond lengths and angles are given in Tables S10 and S11. Despite disorder in some of the TBA cations, important features of the two anion structures are evident. The terminal Sn–O bond of 1.938 Å in [(HO)SnW_5_O_18_]^3–^**6** is longer than the terminal W=O bonds (average 1.725 Å) and is consistent with Sn–OH, while in the SnOW bridges the W–O bonds (average 1.878 Å) are shorter than Sn–O (average 2.012 Å). In the structure of [(μ-O)(SnW_5_O_18_)_2_]^6–^**10**, although one of the {SnW_5_O_18_} oxometalate units is disordered over two orientations, this does not affect the SnOSn linkage, which is bent with a SnOSn angle of 151.4°. This is significantly smaller than NbONb of 180.0° in [(μ-O)(NbW_5_O_18_)_2_]^4–^ and TiOTi of 173.1° in [(μ-O)(TiW_5_O_18_)_2_]^6–^**9** and is indicative of the absence of Sn–O π-overlap that is possible for the Ti and Nb homologues.

### Condensation of Keggin {(HO)MPW_11_} Anions

**Figure 15:**
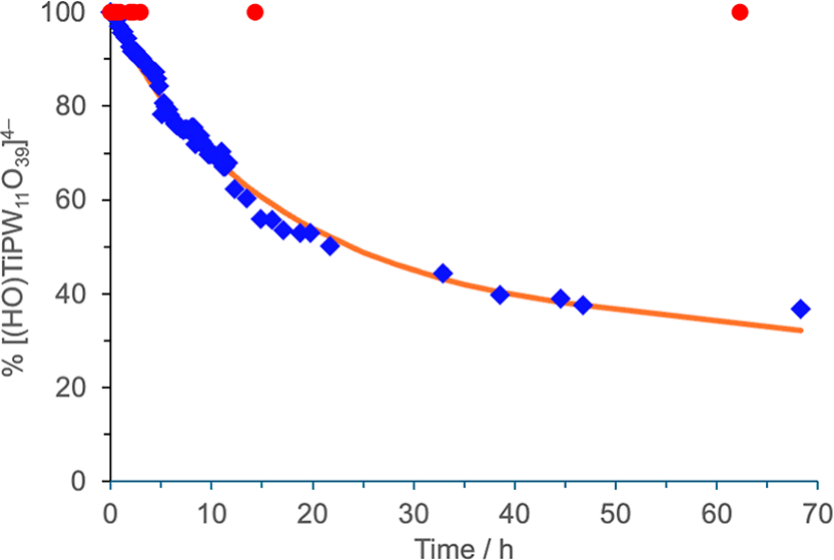
^31^P NMR kinetic plots showing the condensation of (TBA)_4_[(ΗO)TiPW_11_O_39_] (TBA)_8_**7** to (TBA)_8_[(μ-O)(TiPW_11_O_39_)_2_] (TBA)_8_**11** in MeCN (blue ◇) and the lack of reaction for (TBA)_4_[(ΗO)SnPW_11_O_39_] (TBA)_8_**8** (red ●). Line shows least-squares fit (see Supporting Information).

The ^31^P NMR kinetic plots in Figure 15 show that the Keggin species (TBA)_4_**7** undergoes condensation in MeCN solution to (TBA)_8_[(μ-O)(TiPW_11_O_39_)_2_] (TBA)_8_**11**, whereas the tin analogue (TBA)_4_**8** is unreactive under these conditions (eq 6). Interestingly, a solution of (TBA)_4_**7** in DMSO was unchanged after 3 months, suggesting that the better donor solvent inhibits the association required for formation of the transition state and proton transfer.
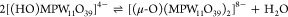
6

**Figure 16:**
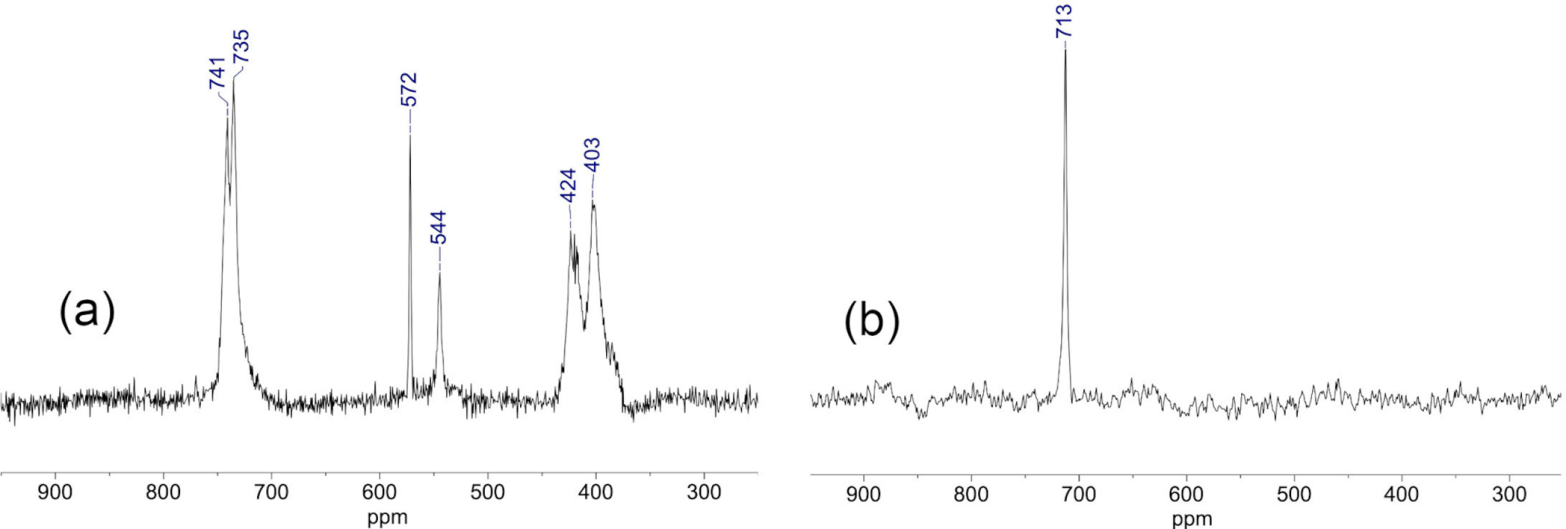
^17^O NMR spectra of (TBA)_8_[(μ-O)(TiPW_11_O_39_)_2_] (TBA)_8_**11** in MeCN with selective ^17^O-enrichment of either the {PTiW_11_O_39_} framework (a) or the bridging TiOTi site (b).

Synthesis of (TBA)_8_**11** was achieved by stirring a solution of (TBA)_4_**7** in MeCN over 3A molecular sieves for 2 days in order to drive the reaction to completion. Samples of (TBA)_8_**11** with selective ^17^O-enrichment of either the {PTiW_11_O_39_} framework or the bridging TiOTi site were prepared from ^17^O-enriched (TBA)_4_**3** and nonenriched water or from nonenriched (TBA)_4_**3** and ^17^O-enriched water respectively, enabling the ^17^O NMR peak at 713 ppm to be assigned to the TiOTi bridging oxygen (Figure 16). The FTIR spectrum of a sample of (TBA)_8_**11** with ^17^O-enrichment at TiOTi contained a characteristic band at 627 cm^–1^ associated with ν_as_(Ti^16/17/18^O) of the TiOTi bridge, as well as ν(PO) at 1066, ν(W=O) at 959 and ν(WOW) at 790 cm^–1^ (Figure S22). The ^183^W NMR spectrum of (TBA)_8_**11** (Figure S23) is similar to that reported by Kholdeeva and co-workers with only minor chemical shift differences.^17^

**Figure 17:**
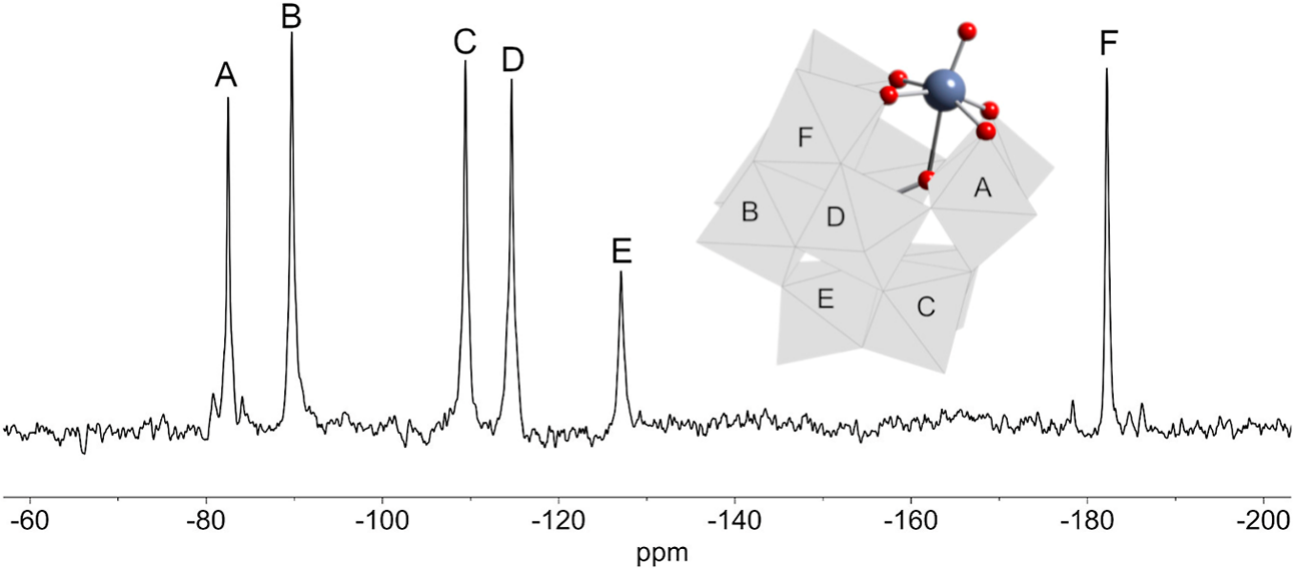
^183^W NMR spectrum of (TBA)_8_[(μ-O)(SnPW_11_O_39_)_2_] (TBA)_8_**12**.

In contrast to the {TiPW_11_} system, addition of 3A molecular sieves had no effect on a solution of (TBA)_4_**8** over a period of 2 weeks, and an attempt to drive the reaction to completion at ca. 120 °C with periodic removal of water under reduced pressure gave only 50% conversion to (TBA)_4_**12** after 12 h (Figure S24). However, complete conversion was achieved using an excess of *N*,*N*′-dicyclohexyl carbodiimide (DCC) as a dehydrating agent (Figure S25). The band at 750 cm^–1^ in the FTIR spectrum of (TBA)_8_**12** is absent in that of (TBA)_4_**8** and might be assigned to either ν(SnOW) or ν_as_(SnO) of the SnOSn bridge, as there is no other obvious band for the bridging vibration (Figure S26). Only a single ν(P–O) band is observed at 1065 cm^–1^ whereas this band is split in the FTIR spectrum of hydroxide (TBA)_4_**8**. The presence of six peaks in the ^183^W NMR spectrum of (TBA)_4_**12** in the ratio 2:2:2:2:1:2 (Figure 17) is consistent with the α-{SnPW_11_} Keggin unit with *C*_*s*_ symmetry. Satellite ^2^*J*(^183^W^119^Sn) coupling of 68 Hz for peak A at −82.5 ppm is associated with the smaller SnOW angles within the SnW_2_ triad, whereas the larger ^2^*J*(^183^W^119^Sn) of 165 Hz for peak F at −182.2 ppm is due to SnOW linkages with larger angles external to the SnW_2_ triad. Other peak assignments in Figure 17 are based on the spectrum of (TBA)_4_**8** as satellite ^2^*J*(^183^W^183^W) couplings were not resolved.

The ^17^O NMR spectrum of (TBA)_8_**12** prepared from ^17^O-enriched (TBA)_4_**8** obtained from ^17^O-enriched (TBA)_4_**4** was poorly resolved and contained broad peaks assigned to W=O at 740–732 ppm and overlapping peaks at 429–374 ppm due to SnOW and WOW (Figure S27). An attempt to determine the SnOSn ^17^O chemical shift by heating a sample of hydroxide (TBA)_4_**8** that had been selectively ^17^O-enriched at SnOH was unsuccessful. The complexity of the ^119^Sn NMR resonance of (TBA)_4_**12** meant that ^2^*J*(^119^Sn^117^Sn) could not be resolved, and we were unable to obtain a ^31^P-decoupled spectrum. Instead, simulation of the ^119^Sn NMR spectrum using ^2^*J*(^31^P^119^Sn) and ^2^*J*(^183^W^119^Sn) values from ^31^P and ^183^W NMR spectra while varying the value of ^2^*J*(^119^Sn^117^Sn) from 0 to 350 Hz gave best fits with ^2^*J*(^119^Sn^117^Sn) values of either 60 or 110 Hz (see Figure S28). The larger of these would seem to be most likely, given that ^2^*J*(^119^Sn^117^Sn) for the Lindqvist analogue **10** was observed to be 333 Hz.

### Kinetic Analysis

In an attempt to derive quantitative comparisons, data from the CD_3_OD exchange, hydrolysis and condensation time-resolved NMR experiments were analyzed according to eqs 1–4 and 6 using least-squares curve-fitting as described in the Supporting Information. The parameters obtained are summarized in Table S12 together with data obtained from analysis of 2D ^1^H EXSY NMR experiments, although it should be noted that the precision was limited by the nature of the experiments, particularly for the {SnOMe}-substituted POMs which are much more moisture-sensitive and undergo rapid exchange and hydrolysis.

### DFT Modeling

Further insight into the kinetic and thermodynamic effects resulting from metal substitution and the different POM structural types was obtained from DFT modeling of the reaction steps involved in these alcoholysis, hydrolysis and condensation processes. A feature of proton migration reactions in POM chemistry is the involvement of framework oxygens, and we were interested to explore the role of the basic MOW bridging oxygens in the reactivity of these Lindqvist and Keggin anions.

#### Alcoholysis

To investigate the origin of the activation energies and explain the faster methanol–methoxide exchange observed for the {SnW_5_} anion **2** compared with {TiW_5_} anion **1**, two protonolysis mechanisms were considered. The intermediates **Int-1** or **Int-2** arising from interaction of the MeOH hydroxyl proton with oxygen of either MOC or MOW respectively are shown in Figure S29. The results in Table S13 show that for {TiW_5_} anion **1** interaction of MeOH with TiOW is preferred, although **Int-2** is only ca. 2 kcal mol^–1^ more stable than **Int-1**. For the {SnW_5_} anion **2**, energies and distances in Table S13 indicate a slight preference for the alternative **Int-1** due to the more ionic nature of the Sn–OMe bond and the greater basicity of the SnOMe oxygen.^20^ Calculations showed that the alcoholysis reaction mechanism for {TiW_5_} anion **1** proceeds from the initial interaction at TiOW in **Int-2** via an approach between MeOH and the Ti, leading to a transition state which contains seven-coordinate TiOM and facilitates the proton transfer from the MeOH to the alkoxido ligand with an energy barrier of 16.4 kcal·mol^–1^. Analogous calculations for {SnW_5_} anion **2** gave a similar energy profile with an energy barrier of 11.7 kcal·mol^–1^. The calculated transition state structures for MeOH exchange with methoxido anions {TiW_5_} **1** and {SnW_5_} **2** are shown in Figure S30. Given the lower energies associated with **Int-2** resulting from the initial approach of MeOH at TiOW, the migration that would convert **Int-2** to **Int-1** was studied for **1**. Very small energy barriers were found with an activation energy of only 2.5 kcal·mol^–1^, suggesting that this pathway is likely to be spontaneous under reaction conditions.

#### Hydrolysis of Lindqvist Alkoxido Anions

Having verified the alcoholysis mechanism, we then characterized in detail the mechanism for protonolysis with H_2_O to establish the thermodynamic and kinetic origins of the differences in hydrolytic behaviors of Lindqvist anions **1** and **2**. Calculations were also performed on the niobium homologue [(MeO)NbW_5_O_18_]^2–^, for which we previously established qualitatively that both hydrolysis and subsequent condensation are facile.^7^

**Figure 18:**
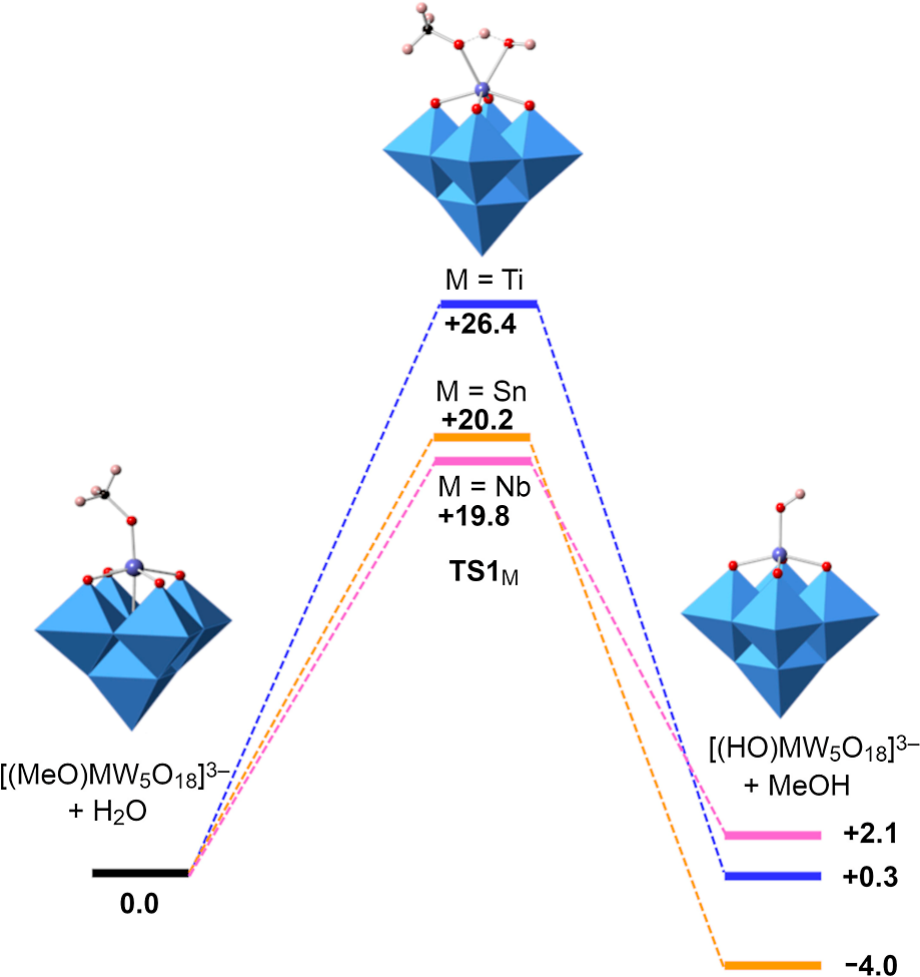
Calculated Gibbs free energy profiles (kcal·mol^–1^) for hydrolysis of methoxido Lindqvist anions [(HO)TiW_5_O_18_]^3–^**5** (blue), [(HO)SnW_5_O_18_]^3–^**6** (orange) and [(HO)NbW_5_O_18_]^2–^ (purple).

The results summarized in Figure 18 and Table S14 show that the energy barrier for {TiW_5_} **1** (26.4 kcal·mol^–1^) is higher than for {SnW_5_} **2** (20.2 kcal·mol^–1^) while the lowest barrier (19.8 kcal·mol^–1^) was found for the {NbW_5_} anion in accordance with our observations that hydrolysis of **2** and [(MeO)NbW_5_O_18_]^2–^ is faster than **1**. Moreover, the reaction energies are consistent with the easier isolation of the tin-substituted hydroxide (TBA)_3_**6** as calculations show that hydrolysis is a slightly exothermic process (−4 kcal·mol^–1^), whereas this is not the case for the {TiW_5_} hydroxide **5** or [(HO)NbW_5_O_18_]^2–^ for which hydrolysis shows almost no energy change respect to reactants. In the transition state structures shown in Figure S31, the calculated M–OR bond distances in TS1_Sn_ and TS1_Nb_ are longer than in TS1_Ti_ and therefore should be easier to cleave, which is consistent with the greater hydrolytic sensitivity of (TBA)_3_**2** and (TBA)_2_[(MeO)NbW_5_O_18_]. In addition, the O–H bond for the migrating proton in H_2_O is longer in TS1_Nb_, which is again consistent with more facile hydrolysis of [(MeO)NbW_5_O_18_]^2–^.

#### Condensation of Lindqvist Hydroxido Anions

**Figure 19:**
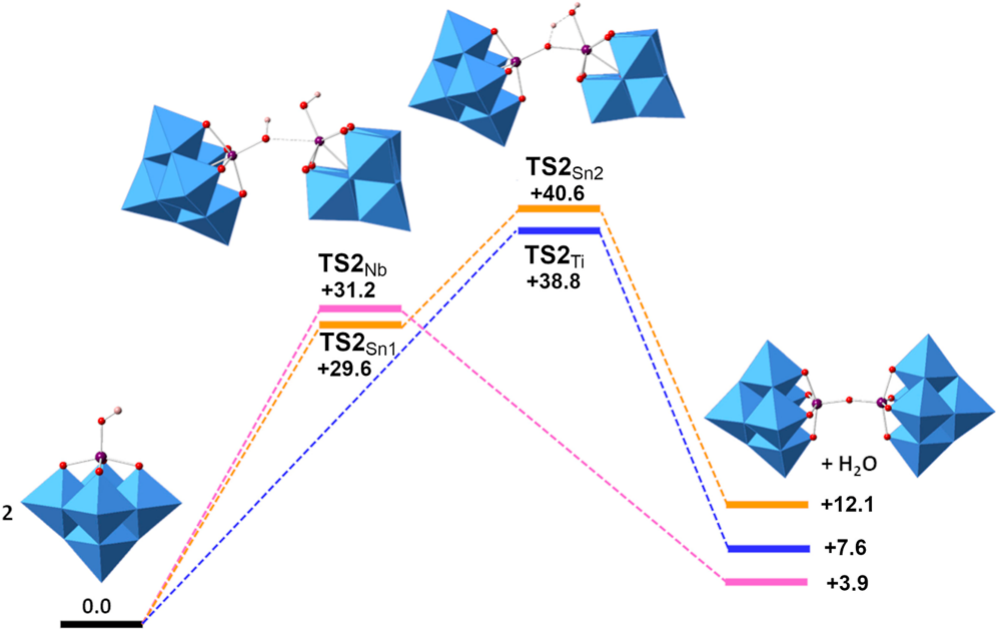
Calculated Gibbs energy profiles (kcal·mol^–1^) for condensation of hydroxido Lindqvist anions [(HO)TiW_5_O_18_]^3–^**5** (blue), [(HO)SnW_5_O_18_]^3–^**6** (orange) and [(HO)NbW_5_O_18_]^2–^ (purple).

We then turned our attention to the generation of oxido-bridged anions [(μ-O)(TiW_5_O_18_)_2_]^6–^**9**, [(μ-O)(SnW_5_O_18_)_2_]^6–^**10** and [(μ-O)(NbW_5_O_18_)_2_]^4–^ by condensation of hydroxido species [(HO)TiW_5_O_18_]^3–^**5**, [(HO)SnW_5_O_18_]^3–^**6** and [(HO)NbW_5_O_18_]^2–^ respectively. The energy profiles for the {TiW_5_}, {SnW_5_} and {NbW5} systems are shown in Figure 19 and calculated energies are summarized in Table S15. Two different energy barriers were found in the formation of **10**, corresponding to (i) SnO(H)–Sn bond formation (TS2_Sn1_) and (ii) proton transfer from one SnOH to the other (TS2_Sn2_). However, only one transition state involving both processes, i.e. MO(H)–M formation (M = Ti, Nb) and proton transfer, was found in the formation of **9** and [(μ-O)(NbW_5_O_18_)_2_]^4–^. Transition state structures for the formation of these oxido-bridged anions are shown in Figure S32. In TS2_Ti_, the TiO(H)–Ti bond (2.63 Å) is forming at the same time that the proton is transferred to give **9**. In the {SnW_5_} system, TS2_Sn1_ is the first transition state related to the formation of the SnO(H)–Sn bond (2.71 Å) and TS2_Sn2_ corresponds to the proton transfer leading to **10**. The only transition state found for the {NbW_5_} system TS2_Nb_ is similar to TS2_Sn1_. The relative Gibbs transition state energies confirm that these energy barriers are surmountable under experimental conditions, although one would expect condensation in the {SnW_5_} system to be more demanding because of the two energy barriers en route to **10**. Although we were not able to determine an intermediate between the two energy barriers, this is not necessary for comparison purposes. The condensation reaction to give SnOSn-bridged **10** is endothermic by 12.1 kcal·mol^–1^, which is consistent with the easier isolation of {SnW_5_} hydroxide (TBA)_3_**6**, whereas in the {TiW_5_} system, the condensation reaction to give TiOTi-bridged **9** is less energetically demanding (7.6 kcal·mol^–1^), which explains its accessibility upon heating in order to overcome the activation energy. In the {NbW_5_} system the barrier for condensation is lower (31.2 kcal·mol^–1^) and the process is less endothermic (3.9 kcal·mol^–1^) than for the {M^IV^W_5_} systems.

#### Hydrolysis of Keggin Alkoxido Anions

Analogous energy profiles were calculated for hydrolysis of the methoxido {MPW_11_} Keggin anions [(MeO)TiPW_11_O_39_]^4–^**3** and [(MeO)SnPW_11_O_39_]^4–^**4** (Figure S33 and Table S16). Hydrolysis of **3** is endothermic by 1.8 kcal·mol^–1^, while hydrolysis of **4** is slightly exothermic by −4.0 kcal·mol^–1^. In addition, the energy barrier for hydrolysis of **4** is 26.4 kcal·mol^–1^, whereas hydrolysis of the {TiPW_11_} analogue **3** features a much higher energy barrier (30.3 kcal·mol^–1^). These results are consistent with the greater moisture sensitivity observed for (TBA)_4_**4**. The calculated transition state geometries around the heteroatom in the formation of hydroxido {MPW_11_} anions [(HO)TiPW_11_O_39_]^4–^**7** and [(HO)SnPW_11_O_39_]^4–^**8** were found to be similar to those in the Lindqvist hydrolysis reactions (Figure S34), and similar trends were found regarding M–O bonds, i.e., Sn–O bonds are longer than Ti–O bonds.

#### Condensation of Keggin Hydroxido Anions

Similar trends were found in the formation of the oxido-bridged anions [(μ-O)(TiPW_11_O_39_)_2_]^8–^**11** and [(μ-O)(SnPW_11_O_39_)_2_]^8–^**12** to those for analogous Lindqvist anions [(μ-O)(TiW_5_O_18_)_2_]^6–^**9** and [(μ-O)(SnW_5_O_18_)_2_]^6–^**10**, and the associated energy profiles are shown in Figure S35 and summarized in Table S17. Again, two different energy barriers were found in the formation of {SnPW_11_} **12**, the first associated with formation of the SnO(H)–Sn bond and the second with proton transfer. Only one energy barrier was found for the formation of {TiPW_11_} **11**, implying the same type of concerted process as calculated for the Lindqvist {TiW_5_} system.

The comparative energetics for the Ti and Sn systems are again revealing, and are consistent with experimental observations. While the energy barriers for condensation in each case are similar and accessible under normal reaction conditions, the formation of oxido-bridged {TiPW_11_} species **11** from hydroxide **7** is less endothermic (7.6 kcal·mol^–1^) than the formation of **12** from hydroxide **8** (16.7 kcal·mol^–1^). This explains the difficulty in achieving complete condensation in the {SnPW_11_} system and the ready hydrolysis of (TBA)_8_**12** back to hydroxide (TBA)_4_**8**. In the transition state structure for the formation of **11** (Figure S36, TS2_Ti_) the Ti–O bond (2.25 Å) is forming while the proton is transferred. For the tin analogue **12**, the structure of TS2_Sn1_ shows the formation of SnO(H)–Sn (2.51 Å) while proton transfer occurs in the second transition state TS2_Sn2_.

## Discussion

The {MOR}^3+^ substituted Lindqvist and Keggin anions (M = Ti, Sn; R = Me, H) provide a rare opportunity to study isolated M–OR bonds within a metal oxide environment. The systematic investigations into protonolysis and condensation reactions described here deepens our understanding of the observed reactivity for {(RO)M′M_5_} Lindqvist systems,^7−9,11,12,19−21^ and builds on the seminal work by Maksimov, Kholdeeva and co-workers, who used slightly different conditions to study {TiPW_11_} Keggin species.^16,17,38^ It is generally accepted that rates of alcohol–alkoxido exchange and hydrolysis are determined by the electronegativity and coordination number of the metal and the steric properties of the alkyl group, but very few detailed mechanistic studies have been published, mainly due to rapid reaction rates and complex mechanistics involving aggregation to give polynuclear species when the metal center is bonded to multiple alkoxido ligands. ^1^H NMR magnetization transfer studies by Hampden-Smith provided kinetic parameters for ^t^BuOH exchange with the *tert*-butoxide groups in sterically hindered Sn(O^t^Bu)_4_ and indicated an associative mechanism with a highly ordered transition state,^39^ while Kessler has highlighted the complexities of metal alkoxide sol–gel chemistry.^40^

Our NMR studies revealed some remarkable differences in reactivity for {MOR}^3+^ substituted Lindqvist and Keggin anions and kinetic parameters shown in Table S12 provide a quantitative dimension to our general observations that rates of protonolysis vary not only with the nature of heterometal but also with the nature of the lacunary POM ligand framework. Hence, SnOMe/MeOH exchange rate constants of 0.29 M^–1^ s^–1^ for {(MeO)SnW_5_} **2** and 0.12 M^–1^ s^–1^ for {(MeO)SnPW_11_} **4** were derived from 2D ^1^H EXSY NMR experiments (although solubility differentials, hydrolytic sensitivity and integration inaccuracies due to overlap with intense adjacent peaks limited the precision), while the lack of EXSY exchange peaks for {TiW_5_} **1** and {TiPW_11_} **3** indicates that rates for these anions are significantly lower. Kinetic analysis of ^1^H NMR data for CD_3_OD exchange (see Supporting Information for details) gave rate constants for **1** (*k*_f_ = 0.02 and *k*_b_ = 0.06 M^–1^ s^–1^) that are approximately an order of magnitude slower than the EXSY MeOH exchange rate for **2**. The CD_3_OD exchange rate constants for **3** (*k*_f_ = 4 × 10^–4^ and *k*_b_ = 1 × 10^–3^ M^–1^ s^–1^) are much slower that the corresponding values for **1** and **4** (values of *k*_f_ = 3 × 10^–2^ and *k*_b_ = 8 × 10^–2^ M^–1^ s^–1^ represent lower limits because of the limited number of data points during initial stages of the rapid reaction as shown in Figure 2b). Note that a kinetic isotope effect is expected for the reactions involving O–D bonds and this should be borne in mind when comparing CD_3_OD exchange rates with the MeOH exchange rates from EXSY experiments. Kinetic data for M–OMe hydrolysis reactions show the same trends, with greater rate constants for {(MeO)TiW_5_} **1** than for {(MeO)TiPW_11_} **3**. Reliable hydrolysis data could not be obtained for {(MeO)SnW_5_} **2** because of its much greater reactivity, but {(MeO)SnPW_11_} **4** was sufficiently less reactive to allow approximate lower limits of *k*_f_ = 0.08 and *k*_b_ = 0.1 M^–1^ s^–1^ to be determined. These results clearly show that the Sn–OMe bonds in **2** and **4** are more reactive toward protonolysis than Ti–OMe in **1** and **3**, which is consistent with our previous studies of homologous [(RO)MW_5_O_18_]^3–^ anions (M = Ti, Sn), in which calculations of molecular electrostatic potentials and atomic charges on **1** and **2** indicated that the more ionic Sn–OR bonds increase the basicity of the SnOR oxygen and the electrophilicity of Sn.^20^ Another key finding is that the M–OMe bonds in {(MeO)MW_5_} Lindqvist anions are more reactive than in the corresponding {(MeO)MPW_11_} Keggin anions, which may be due to greater charge densities associated with the oxygens of the smaller anions, although geometric factors might also be involved. The kinetic data also indicate more favorable hydrolysis in DMSO, possibly due to different p*K*_a_ values for H_2_O in DMSO vs MeCN, while the apparently anomalous lower hydrolysis rates observed for **1** with a large excess of water might be ascribed to a change in solvent properties as the proportion of water is increased, although this was not observed for **3**, which has a lower overall negative charge density. These observations are interesting and are indicative of the complex factors affecting these nonaqueous protonolysis reactions, but more detailed investigations were beyond the scope of this work.

The DFT calculations are in general agreement with these results and provide additional insight into the mechanisms of protonolysis, including preassociation of proton donors with the basic MOW sites. In the case of MeOH exchange with **2**, the SnOMe site is competitive (Figure S29), while the energy differences between the two sites for **1** are small and spontaneous progression to the transition states shown in Figure S30 is expected under reaction conditions.

The isolation and characterization of the terminal MOH derivatives (M = Ti, Sn) was an important achievement which enabled subsequent condensation reactions to be studied. Of the Lindqvist anions, {(HO)SnW**5**} **6** is most easily prepared by virtue of the facile hydrolysis of **2** in MeCN, whereas isolation of {(HO)TiW_5_} **5** required the use of DMSO as solvent to promote hydrolysis of **1** and inhibit condensation by virtue of its better ligand properties than MeCN. The {(HO)MPW_11_} Keggin derivatives **7** and **8** were prepared similarly and ^31^P NMR showed that **7** underwent condensation slowly at room temperature, but **8** was much more stable (Figure 15), while ^119^Sn NMR showed that **6** was also reluctant to undergo condensation. In all cases, removal of H_2_O from condensation reaction mixtures by distillation, absorption by 3A molecular sieves or reaction with DCC enabled isolation of MOM-bridged products **9–12**. This was essential for condensation of Sn-substituted **6** and **8** because of the instability of the SnOSn bridge in **10** and **12** toward hydrolysis. Together with our previous report of the hydrolytic sensitivity of [(MeO)NbW_5_O_18_]^2–^,^7^ these results establish an order for decreasing MOM bridge stability of Nb > Ti > Sn that is consistent with the energetics obtained from the DFT calculations.

## Conclusion

We have obtained the first comparative kinetic data for protonolysis reactions of a series of {MOR}^3+^ substituted Lindqvist and Keggin anions (M = Ti, Sn). For alcohol exchange and hydrolysis reactions, we found that not only were the Sn derivatives more reactive than the Ti analogues, but also that rates are greater for the smaller Lindqvist anions with higher charge densities. An interesting feature is the solvent dependence of hydrolysis which warrants further investigation, but was beyond the scope of this work. All four homologous terminal hydroxido derivatives have been isolated which, for the Ti-substituted anions, required the hydrolysis conditions to be carefully controlled to prevent condensation to TiOTi bridged [(μ-O)(TiW_5_O_18_)_2_]^6–^**9** or [(μ-O)(TiPW_11_O_39_)_2_]^8–^**11**. The {(HO)SnW_5_} and {(HO)SnPW_11_} anions **6** and **8** respectively were reluctant to undergo condensation, but all four MOM-bridged homologues **9–12** were obtained by removing H_2_O from the condensation reaction mixture. Reaction mechanisms and energy parameters associated with protonolysis and condensation processes were obtained for {MW_5_} (M = Ti, Sn, Nb) and {MPW_11_O_39_} (M = Ti, Sn) homologues by DFT modeling and results were broadly consistent with experimental observations. This work provides important insight into proton transfer reactions in metal oxide and alkoxide systems and demonstrates the major effects of metal substitution in polyoxometalates as well as the influence of anion structure and charge density on reactivity.

## Supporting Information Available

A file containing details of kinetics analysis, NMR and FTIR spectra, X-ray crystal structures and DFT calculations (PDF)
