# Supplementary material for: Protonolysis and Condensation Reactions of Alkoxido-Substituted Lindqvist {MW5} and Keggin {MPW11} Polyoxometalates: Comparative Experimental and Modeling Studies
Source: Inorg Chem. 2025 Jan 29;64(5):2379–93. doi: 10.1021/acs.inorgchem.4c04636 (PMC11815849; doi:10.1021/acs.inorgchem.4c04636)
Supplement: Supplementary file 1 — ic4c04636_si_001.pdf [file ic4c04636_si_001.pdf]

Supporting Information

Protonolysis and condensation reactions of alkoxido-substituted Lindqvist {MW_5} and Keggin {MPW_{11}} polyoxometalates: comparative experimental and modelling studies.

Daniel Lebbie,[a] Thompson Izuagie,[a] Magda Pascual-Borràs,[a] Balamurugan Kandasamy,[a] Corinne Wills,[a] Paul. G. Waddell,[a] Benjamin R. Horrocks,[a] and R. John Errington* [a].

NUPOM Lab, Chemistry, School of Natural and Environmental Sciences, Newcastle University, Newcastle upon Tyne, NE1 7RU, UK

John.Errington@newcastle.ac.uk

Contents

		page
**Analysis of kinetic data**		**5-6**
**Table S1**	^{17}O NMR data for {MW_5} Lindqvist anions (M = Ti, Sn).	**6**
**Table S2**	^{183}W and ^{119}Sn and NMR data for {MW_5} Lindqvist anions (M = Ti, Sn).	**6**
**Table S3**	^{17}O NMR data for {MPW_{11}} Keggin anions (M = Ti, Sn).	**6**
**Table S4**	^{31}P and ^{119}Sn NMR data for {MPW_{11}} Keggin anions (M = Ti, Sn).	**7**
**Table S5**	^{183}W NMR data for {MPW_{11}} Keggin anions (M = Ti, Sn).	**7**
**Table S6**	^{1}H NMR T_1 relaxation times for $MOCH_3$ in anions **1** – **4**.	**7**
**Figure S1**	2D ^{1}H EXSY NMR spectra in CD_3CN of 0.05 M $[(MeO)TiW_5O_{18}]^{3-}$ **1** / 0.5 M MeOH (a), 0.013 M $[(MeO)TiPW_{11}O_{39}]^{4-}$ **3** / 0.04 M MeOH (b), 0.03 M $[(MeO)SnW_5O_{18}]^{3-}$ **2** / 0.25 M MeOH (c), 0.028 M $[(MeO)SnPW_{11}O_{39}]^{4-}$ **4** / 0.25 M MeOH (d).	**8**
**Figure S2**	^{1}H NMR spectra in CD_3CN of $(TBA)_3[(MeO)TiW_5O_{18}]$ $(TBA)_3$**1** 2 h after addition of 55 mole-equivalents of H_2O (left) and of $(TBA)_3[(MeO)SnW_5O_{18}]$ $(TBA)_3$**2** immediately after addition of 12 mole-equivalents of H_2O (right).	**8**
**Figure S3**	^{31}P NMR spectra showing hydrolysis of $(TBA)_4[(MeO)TiPW_{11}O_{39}]$ $(TBA)_3$**3** to $(TBA)_4[(HO)TiPW_{11}O_{39}]$ $(TBA)_3$**7** (a) and $(TBA)_4[(MeO)SnPW_{11}O_{39}]$ $(TBA)_3$**4** to $(TBA)_4[(HO)SnPW_{11}O_{39}]$ $(TBA)_3$**8** (b) in MeCN.	**9**
**Figure S4**	^{119}Sn NMR spectra for hydrolysis of $(TBA)_3[(MeO)SnW_5O_{18}]$ $(TBA)_3$**2** to $(TBA)_3[(HO)SnW_5O_{18}]$ $(TBA)_3$**6** (a) and $(TBA)_4[(MeO)SnPW_{11}O_{39}]$ $(TBA)_3$**4** to $(TBA)_4[(HO)SnPW_{11}O_{39}]$ $(TBA)_3$**8** (b) in MeCN.	**9**

Figure S5 FTIR spectrum of $(TBA)_3[(HO)TiW_5O_{18}]^{3-}$ $(TBA)_3$**5** and part of the spectrum obtained after deuteration. **10**
Figure S6 ^{1}H NMR spectrum of $(TBA)_3[(HO)TiW_5O_{18}]$ $(TBA)_3$**5** in *d*$_6$-DMSO. **10**
Figure S7 ^{17}O NMR spectrum (in CD_3CN) of product obtained by treatment of non-^{17}O-enriched $(TBA)_3[(HO)TiW_5O_{18}]$ $(TBA)_3$**5** in DMSO with ^{17}O-enriched H_2O. **10**
Figure S8 One of the disordered anions in the structure of $(TBA)_3[(HO)TiW_5O_{18}]$ $(TBA)_3$**5** **11**
Table S7 Crystallographic data for $(TBA)_3[(HO)TiW_5O_{18}]$.MeCN $(TBA)_3$**5**.MeCN. **11**
Table S8 Bond distances in the *trans*-disordered anion of $(TBA)_3[(HO)TiW_5O_{18}]$.MeCN $(TBA)_3$**5**. **12**
Figure S9 FTIR spectrum of incompletely deuteriated $(TBA)_3[(HO)SnW_5O_{18}]$ $(TBA)_3$**6**. **12**
Figure S10 ^{17}O NMR spectrum of $(TBA)_3[(HO)SnW_5O_{18}]$ $(TBA)_6$**6**. **12**
Figure S11 FTIR spectrum of $(TBA)_4[(HO)TiPW_{11}O_{39}]$ $(TBA)_4$**7** **13**
Figure S12 ^{1}H NMR spectrum of $(TBA)_4[(HO)TiPW_{11}O_{39}]$ $(TBA)_4$**7** in $(CD_3)_2SO$. **13**
Figure S13 ^{17}O NMR spectrum of $(TBA)_4[(HO)TiPW_{11}O_{39}]$ $(TBA)_4$**7** in CD_3CN. **14**
Figure S14 FTIR spectrum of $(TBA)_4[(HO)SnPW_{11}O_{39}]$ $(TBA)_4$**8** and part of the spectrum after deuteriation. **14**
Figure S15 ^{17}O NMR spectra of (a) $(TBA)_4[(HO)SnPW_{11}O_{39}]$ $(TBA)_4$**8** with enriched $\{SnPW_{11}O_{39}\}$ and (b) product from treatment of non-enriched] $(TBA)_4$**8** with ^{17}O-enriched H_2O. **15**
Figure S16 ^{183}W NMR equilibrium analysis of $(TBA)_3[(HO)TiW_5O_{18}]$ $(TBA)_3$**5** condensation to $(TBA)_6[(\mu\text{-O})(TiW_5O_{18})_2]$ $(TBA)_6$**9** , showing W_{eq} peaks in MeCN (left) and DMSO (right). **15**
Figure S17 ^{119}Sn NMR equilibrium analysis for the condensation of $(TBA)_3[(HO)SnW_5O_{18}]$ $(TBA)_3$**6** to $(TBA)_6[(\mu\text{-O})(SnW_5O_{18})_2]$ $(TBA)_6$**10**. **16**
Figure S18 ^{119}Sn NMR study of $[(HO)SnW_5O_{18}]^{3-}$ **6** condensation to $[(\mu\text{-O})(SnW_5O_{18})_2]^{6-}$ **10** in hot PhCN with periodic removal of H_2O under reduced pressure. **16**
Figure S19 ^{17}O NMR spectrum of $(TBA)_6[(\mu\text{-O})(SnW_5O_{18})_2]$ $(TBA)_6$**10. Peaks** marked with an asterisk are due to $(TBA)_2[W_6O_{19}]$ impurity **17**
Figure S20 FTIR spectrum of $(TBA)_6[(\mu\text{-O})(SnW_5O_{18})_2]$ $(TBA)_6$**10**. The band at 2117 cm^{-1} is due to *N, N'*-dicyclohexylcarbodiimide (DCC) impurity. **17**
Table S9 Crystallographic data for $(TBA)_9[(\mu\text{-O})(SnW_5O_{18})_2].[(HO)SnW_5O_{18}]$.3MeCN. **18**
Figure S21 Structures of the anions **6** (a) and disordered **10** (b) present in the co-crystalline $(TBA)_9[(\mu\text{-O})(SnW_5O_{18})_2].[(HO)SnW_5O_{18}]$.3MeCN. **19**
Table S10. Selected bond distances in the anion $[(HO)SnW_5O_{18}]^{3-}$ **6** from the structure of $(TBA)_9[(\mu\text{-O})(SnW_5O_{18})_2].[(HO)SnW_5O_{18}]$.3MeCN. **19**

Table S11. Selected bond distances and angle in the anion $[(\mu\text{-O})(SnW_5O_{18})_2]^{6-}$ **10** from the structure of $(TBA)_9[(\mu\text{-O})(SnW_5O_{18})_2].[(HO)SnW_5O_{18}].3MeCN$. **19**

Figure S22 FTIR spectrum of $(TBA)_8[(\mu\text{-O})(TiPW_{11}O_{39})_2]$ $(TBA)_8$**11** **20**

Figure S23 ^{183}W NMR spectrum of $(TBA)_8[(\mu\text{-O})(TiPW_{11}O_{39})_2]$ $(TBA)_8$**11** in MeCN. The peak marked with an asterisk is assigned to $[PW_{12}O_{40}]^{3-}$. **20**

Figure S24 ^{31}P NMR study of $[(HO)SnPW_{11}O_{39}]^{4-}$ **8** thermal condensation to $[(\mu\text{-O})(SnPW_{11}O_{39})_2]^{8-}$ **12** by removal of water after repeated heating at 120 °C for the times indicated. **21**

Figure S25 ^{31}P (left) and ^{119}Sn (right) NMR studies of $(TBA)_4[(HO)SnPW_{11}O_{39}]$ $(TBA)_4$**8** condensation to $(TBA)_8[(\mu\text{-O})(SnPW_{11}O_{39})_2]$ $(TBA)_4$**12** with DCC as dehydrating agent. **21**

Figure S26 FTIR spectrum of $(TBA)_8[(\mu\text{-O})(SnPW_{11}O_{39})_2]$ $(TBA)_8$**12** compared with that of $(TBA)_4[(HO)SnPW_{11}O_{39}]$ $(TBA)_4$**8** to highlight the differences in ν(PO) and the band assigned to ν(SnOSn) at 750 cm^{-1}. **22**

Figure S27 ^{17}O NMR spectrum of $(TBA)_8[(\mu\text{-O})(SnPW_{11}O_{39})_2]$ $(TBA)_8$**12** **22**

Figure S28 Observed (lower) and simulated (upper) ^{119}Sn NMR spectra of $(TBA)_8[(\mu\text{-O})(SnPW_{11}O_{39})_2]$ $(TBA)_8$**12** using $^2J(^{119}Sn^{117}Sn)$ values of 60 Hz (a) or 110 Hz (b) for simulation. **23**

Table S12. Kinetic parameters from analysis of NMR data. *K* denotes the equilibrium constant, k_f the forward second order rate constant and k_b the backward rate constant. **23**

Figure S29 Polyhedral representation of **Int-1** and **Int-2** in MOMe/MeOH exchange for anions $[(MeO)TiW_5O_{18}]^{3-}$ **1** and $[(MeO)SnW_5O_{18}]^{3-}$ **2**. Grey polyhedra W; blue M (Ti or Sn); red O; black C; pink H. **24**

Table S13 Relative energies with respect to reactants (kcal·mol^{-1}) and distances (Å) for **Int-1** and **Int-2** in MOMe/MeOH exchanges for anions $[(MeO)TiW_5O_{18}]^{3-}$ **1** and $[(MeO)SnW_5O_{18}]^{3-}$ **2**. **24**

Figure S30 Computed structures of the most favorable transition states for MeOH exchange with $[(MeO)TiW_5O_{18}]^{3-}$ **1** and $[(MeO)SnW_5O_{18}]^{3-}$ **2**. **24**

Table S14 Relative energies with respect to reactants (kcal.mol^{-1}) for transition states and hydrolysis products of methoxido Lindqvist anions $[(MeO)TiW_5O_{18}]^{3-}$ **1** ($TS1_{Ti}$), $[(MeO)SnW_5O_{18}]^{3-}$ **2** ($TS1_{Sn}$) and $[(MeO)NbW_5O_{18}]^{2-}$ ($TS1_{Nb}$). Relative Gibbs free energies (kcal.mol^{-1}) are in parentheses. **25**

Figure S31 Optimized transition state structures for hydrolysis of $[(MeO)TiW_5O_{18}]^{3-}$ **1** ($TS1_{Ti}$), $[(MeO)SnW_5O_{18}]^{3-}$ **2** ($TS1_{Sn}$) and $[(MeO)NbW_5O_{18}]^{2-}$ ($TS1_{Nb}$). Grey

	polyhedra W; red O; blue Ti; purple Sn; green Nb; black C; pink H. Distances in Å.	**25**
**Table S15**	Relative energies with respect to reactants (kcal.mol^{-1}) for transition states and products for condensation of hydroxido anions **5**, **6** and $[(HO)NbW_5O_{18}]^{2-}$. Relative Gibbs free energies (kcal.mol^{-1}) are in parentheses.	**25**
**Figure S32**	Optimized transition state structures for the formation of $[(\mu\text{-}O)(TiW_5O_{18})_2]^{6-}$ **9** (TS2$_{Ti}$), $[(\mu\text{-}O)(NbW_5)_2]^{4-}$ (TS2$_{Nb}$) and $[(\mu\text{-}O)(SnW_5O_{18})_2]^{6-}$ **10** (TS2$_{Sn1}$ and TS2$_{Sn2}$). Grey polyhedra: W; red: O; blue: Ti; green: Nb; purple: Sn; pink: H. Distances in Å.	**26**
**Figure S33**	Energy profiles for hydrolysis of methoxido Keggin anions **5** (blue) and **6** (red).	**26**
**Table S16**	Relative energies with respect to reactants (kcal.mol–1) of the transition states and products for hydrolysis of methoxido Keggin anions 3 and 4. Relative Gibbs free energies (kcal.mol–1) are in parenthesis.	**27**
**Figure S34**	Optimized transition state structures in the formation of $[(HO)TiPW_{11}O_{39}]^{4-}$ **7** (TS1$_{Ti}$) and $[(HO)SnPW_{11}O_{39}]^{4-}$ **8** (TS1$_{Sn}$). Color code: Grey polyhedra-W, pink polyhedra-P, red-O, purple-Ti or Sn, black-C and pink-H.	**27**
**Figure S35**	Energy profiles for condensation of hydroxides $[(HO)TiPW_{11}O_{39}]^{4-}$ **7** (blue) and $[(HO)SnPW_{11}O_{39}]^{4-}$ **8** (red). Relative Gibbs free energies (kcal·mol^{-1}) are in parentheses.	**27**
**Table S17**	Relative energies with respect to reactants (kcal.mol^{-1}) of transition states and products for condensation of hydroxido {MPW$_{11}$} anions **7** and **8**. Relative Gibbs free energies (kcal.mol^{-1}) are in parenthesis.	**28**
**Figure S36**	Optimized transition state structures for the formation of $[(\mu\text{-}O)(TiPW_{11}O_{39})_2]^{8-}$ **11** (TS2$_{Ti}$) and $[(\mu\text{-}O)(SnPW_{11}O_{39})_2]^{8-}$ **12** (TS2$_{1Sn}$ and TS2$_{2Sn}$). Color code: Grey polyhedra-W pink polyhedra-P, red-O, purple-Ti or Sn and pink-H.	**28**

Analysis of kinetic data.

^{1}H or ^{31}P NMR spectroscopy was used to observe the kinetics of methanolysis and hydrolysis of the {(MeO)MW_5} anions **1** and **2**, the {(MeO)MPW_{11}} anions **3** and **4** and the condensation of the {(HO)MPW_{11}} anions **7** and **8**. The data were plotted as percentage of POM reactant against time as determined from the intensity of the relevant MOMe (^{1}H) or central P (^{31}P) NMR peaks and analyzed according to a reversible, second order reaction scheme appropriate for bimolecular steps in both directions:

$$A + B \rightleftharpoons C + D \tag{S1}$$

The rate constants for the forward and backward process denoted as k_f and k_b respectively. In Equation S1, *A* denotes the {(MeO)MW_5} or {(MeO)MPW_{11}} reactant (M = Ti or Sn), *B* denotes either CD_3OD (methanolysis) or H_2O (hydrolysis), *C* denotes the {(RO)MW_5} or {(RO)MPW_{11}} product (R = CD_3 or H) and *D* is MeOD (methanolysis) or MeOH (hydrolysis). An analytical solution of the rate equations for arbitrary stoichiometry is cumbersome and therefore a simple explicit finite difference scheme was applied to integrate the rate laws (*e.g.*, Equation S2 and similar for B, D) using the known reaction stoichiometry.

$$\frac{d[A]}{dt} = -k_f[A][B] + k_b[C][D] \tag{S2a}$$

$$\frac{d[C]}{dt} = k_f[A][B] - k_b[C][D] \tag{S2b}$$

The values of the rate constants were obtained by the method of least squares and the equilibrium constant was estimated from the optimized values as $K = k_f/k_b$.

The condensation of *A* = $(TBA)_4[(HO)TiPW_{11}O_{39}]$ was also studied and described by the mechanism:

$$2A \rightleftharpoons C + D \tag{S3}$$

With rate laws,

$$\frac{d[A]}{dt} = -k_d[A]^2 + k_b[C][D] \tag{S4a}$$

$$\frac{d[C]}{dt} = k_d[A]^2 - k_b[C][D] \tag{S4b}$$

Finally, as a check on the kinetic analysis of the hydrolysis of $(TBA)_4[(MeO)TiPW_{11}O_{39}]$, we allowed for the possibility of a condensation step following the initial hydrolysis. Analysis of the data for condensation of $(TBA)_4[(HO)TiPW_{11}O_{39}]$ provided a rate constant for the dimerisation step:

$$2C \rightarrow products \tag{S5}$$

This requires an extra term in the rate law for *C* = $(TBA)_4[(HO)TiPW_{11}O_{39}]$ when considering the hydrolysis of *A* = $(TBA)_4[(MeO)TiPW_{11}O_{39}]$:

$$\frac{d[C]}{dt} = k_f[A][B] - k_b[C][D] - k_d[C]^2 \tag{S6}$$

However, we find that for the measured values of condensation rate, the effect of this term is negligible.

2D ^{1}H NMR EXSY data were analyzed to obtain the pseudo-first order rate constant

$$k = k_f([A] + [B]) + k_b([C] + [D])$$

Bearing in mind the stoichiometry and that $k_f/k_b = 1$ for the pure exchange reaction, the pseudo-first order rate constant from 2D ^{1}H NMR EXSY experiments can be related to the true rate constant k_f simply by dividing by the total concentration of all reactants present in the solution. This allows comparison with values of k_f obtained from the kinetic plots.

Table S1 ^{17}O NMR data for {MW_5} Lindqvist anions (M = Ti, Sn).

Anion[a]		δ_O/ppm					Solvent
		W=O	MOM	MOW	WOW	μ_6-O	
$[(MeO)TiW_5O_{18}]^{3-}$	**1**	721, 713	-	525	390, 380	–58	MeCN
$[(MeO)SnW_5O_{18}]^{3-}$	**2**	720, 684	-	395	383, 364	17	MeCN
$[(HO)TiW_5O_{18}]^{3-}$	**5**	722, 716	-	526	391, 381	–56	dmso
$[(HO)SnW_5O_{18}]^{3-}$	**6**	722, 685	-	397	384, 367	18	MeCN
$[(\mu\text{-}O)(TiW_5O_{18})_2]^{6-}$	**9**	721, 714	679	534	389, 381	–63	MeCN
$[(\mu\text{-}O)(SnW_5O_{18})_2]^{6-}$	**10**	720, 681	na	396	384[b]	18	MeCN

[a] As $^nBu_4N^+$ salts. [b] Overlapping broad peaks; na = not assigned.

Table S2 ^{183}W and ^{119}Sn NMR data for {MW_5} Lindqvist anions (M = Ti, Sn).

Anion[a]		δ_W/ppm		δ_{Sn}/ppm	$^2J(^{119}Sn^{183}W)$/Hz	Solvent
		W_{eq}	W_{ax}	(linewidth/Hz)		
$[(MeO)TiW_5O_{18}]^{3-}$	**1**	32.3	64.5	-	-	MeCN
$[(MeO)SnW_5O_{18}]^{3-}$	**2**	76.9	–128.1	–648 (8)	38 (eq), 12 (ax)	MeCN
$[(HO)TiW_5O_{18}]^{3-}$	**5**	37.4	76.0	-	-	dmso
$[(HO)SnW_5O_{18}]^{3-}$	**6**	72.2	–130.8	–634 (7)	37 (eq), 13 (ax)	MeCN
$[(\mu\text{-}O)(TiW_5O_{18})_2]^{6-}$	**9**	32.1<br>39.4	73.7<br>80.8	-<br>-	-	MeCN<br>dmso
$[(\mu\text{-}O)(SnW_5O_{18})_2]^{6-}$	**10**	70.7	–120.7	–667 (12)	45 (eq), nr (ax)	MeCN

[a] As $^nBu_4N^+$ salts. nr = not resolved

Table S3 ^{17}O NMR data for {MPW_{11}} Keggin anions (M = Ti, Sn).

Anion[a]		δ_O/ppm				Solvent
		W=O	MOM	MOW	WOW	
$[(MeO)TiPW_{11}O_{39}]^{4-}$	**3**	740 - 738	-	559, 534	423 - 382	MeCN
$[(MeO)SnPW_{11}O_{39}]^{4-}$	**4**	745 - 731	-	427 - 333		MeCN
$[(HO)TiPW_{11}O_{39}]^{4-}$	**7**	739 - 736	-	559, 536	424 - 384	MeCN
$[(HO)SnPW_{11}O_{39}]^{4-}$	**8**	746 - 733	-	427 - 334		MeCN
$[(\mu\text{-}O)(TiPW_{11}O_{39})_2]^{8-}$	**11**	742 - 738	713	572, 544	423 - 404	MeCN
$[(\mu\text{-}O)(SnPW_{11}O_{39})_2]^{8-}$	**12**	740 - 738	*b*	429 - 374		MeCN

[a] As $^nBu_4N^+$ salts. [b] not assigned.

Table S4 ^{31}P and ^{119}Sn and ^{1}H NMR data for {MPW_{11}} Keggin anions (M = Ti, Sn).

Anion[a]		δ_P/ppm	δ_{Sn}/ppm	$^2J(^{119}Sn^{31}P)$/Hz	$^2J(^{119}Sn^{183}W)$/Hz	δ_H/ppm	$^2J(Sn^1H)$/Hz[b]	Solvent
$[(MeO)TiPW_{11}O_{39}]^{4-}$	**3**	–14.05<br>–14.25	–<br>–	–<br>–	–<br>–	4.31	–<br>–	MeCN<br>dmso
$[(MeO)SnPW_{11}O_{39}]^{4-}$	**4**	–12.66	–622.1	37	58, 149	3.68	81, 78	MeCN
$[(HO)TiPW_{11}O_{39}]^{4-}$	**7**	–14.14<br>–14.35	–<br>–	–<br>–	–<br>–	 12.04	–<br>–	MeCN<br>dmso
$[(HO)SnPW_{11}O_{39}]^{4-}$	**8**	–12.60	–600.2	34	56, 149	1.98<br>3.98	48<br>42	MeCN<br>dmso
$[(\mu\text{-}O)(TiPW_{11}O_{39})_2]^{8-}$	**11**	–14.07<br>–14.28	–<br>–	–<br>–	–<br>–	–<br>–	–<br>–	MeCN<br>dmso
$[(\mu\text{-}O)(SnPW_{11}O_{39})_2]^{8-}$	**12**	–12.64	–620.4	28	67, 164	–	–	MeCN

[a] As $^nBu_4N^+$ salts. [b] $^2J(^{119}Sn^1H)$, $^2J(^{117}Sn^1H)$

Table S5 ^{183}W NMR data for {MPW_{11}} Keggin anions (M = Ti, Sn).

Anion[a]		δ_W/ppm ($^2J^{119}Sn^{183}W$)[b]						Solvent
$[(MeO)TiPW_{11}O_{39}]^{4-}$	**3**[c]	–86.6	–94.2	***–97.0***	–101.6	–109.6	–113.3	MeCN
$[(MeO)SnPW_{11}O_{39}]^{4-}$	**4**	–70.8 (58)	–91.5	–109.3	–115.1	***–130.9***	–169.7 (150)	MeCN/MeOH
$[(HO)TiPW_{11}O_{39}]^{4-}$	**7**	–81.5 (br)	–90.7	***–94.6***	–98.8	–108.9	–110.2	dmso
$[(HO)SnPW_{11}O_{39}]^{4-}$	**8**	–76.4 (54)	–90.4	–108.7	–114.1	***–128.7***	–175.2 (145)	MeCN
$[(\mu\text{-}O)(TiPW_{11}O_{39})_2]^{8-}$	**11**	–89.9	–91.4	***–92.2***	–97.1	–108.6	–109.2	MeCN
$[(\mu\text{-}O)(SnPW_{11}O_{39})_2]^{8-}$	**12**	–82.5 (68)	–89.7	–109.4	–111.7	***–127.1***	–182.2 (164)	MeCN

[a] As $^nBu_4N^+$ salts. [b] Unique site indicated in bold italics. [c] Data from: Kholdeeva, O. A.; Trubitsina, T. A.; Maksimov, G. M.; Golovin, A.; Maksimovskaya, R. I. *Inorg. Chem.* **2005**, *44*, 1635-1642.

Table S6 ^1H NMR T_1 relaxation times for $MOCH_3$ protons in anions **1 – 4**.

Anion	T_1/sec	Solvent
**1**	4.70	CD_3CN
**1**	2.79	$(CD_3)_2SO$
**2**	4.47	CD_3CN
**3**	2.92	CD_3CN
**4**	10.79	CD_3CN

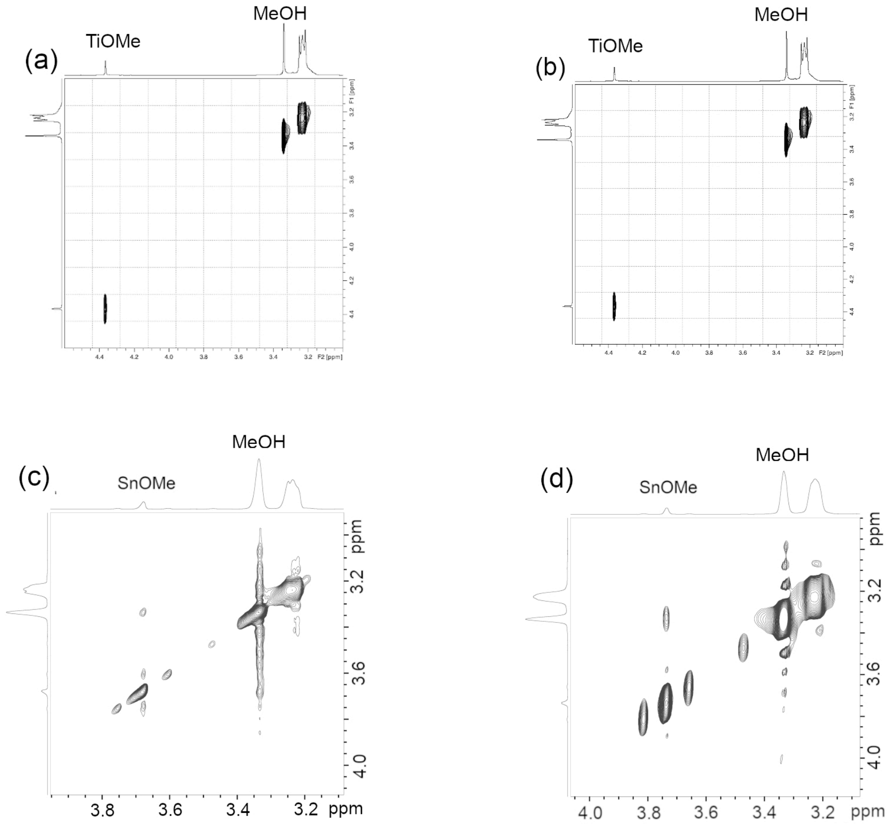

Figure S1 2D ^{1}H EXSY NMR spectra in CD_3CN of 0.05 M $[(MeO)TiW_5O_{18}]^{3-}$ **1** / 0.5 M MeOH (a), 0.013 M $[(MeO)TiPW_{11}O_{39}]^{4-}$ **3** / 0.04 M MeOH (b), 0.03 M $[(MeO)SnW_5O_{18}]^{3-}$ **2** / 0.25 M MeOH (c), 0.028 M $[(MeO)SnPW_{11}O_{39}]^{4-}$ **4** / 0.25 M MeOH (d).

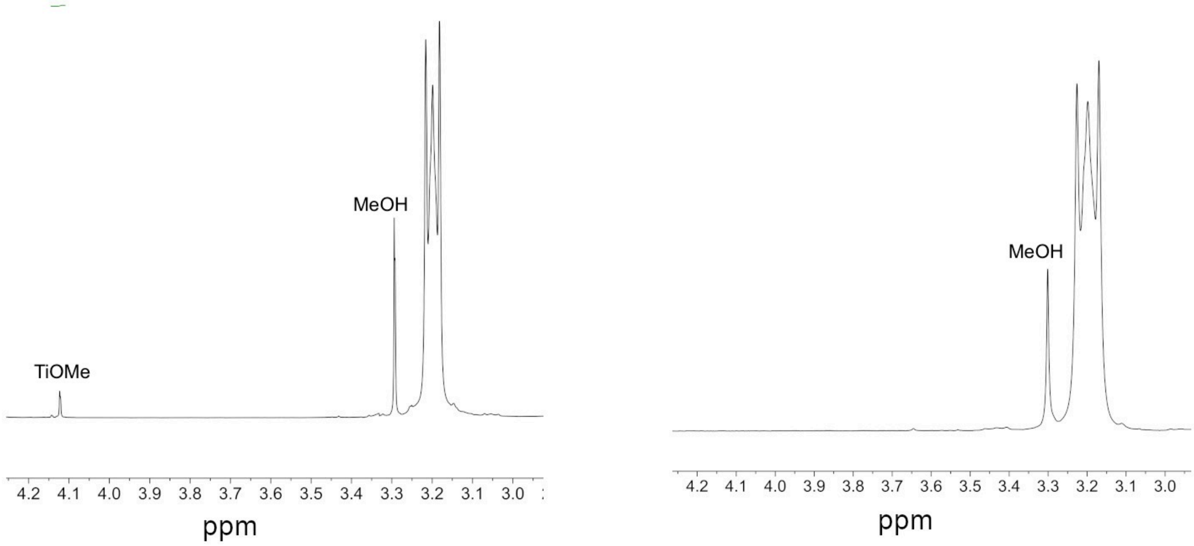

Figure S2 ^{1}H NMR spectra in CD_3CN of $(TBA)_3[(MeO)TiW_5O_{18}]$ $(TBA)_3$**1** 2 h after addition of 55 mole-equivalents of H_2O (left) and of $(TBA)_3[(MeO)SnW_5O_{18}]$ $(TBA)_3$**2** immediately after addition of 12 mole-equivalents of H_2O (right).

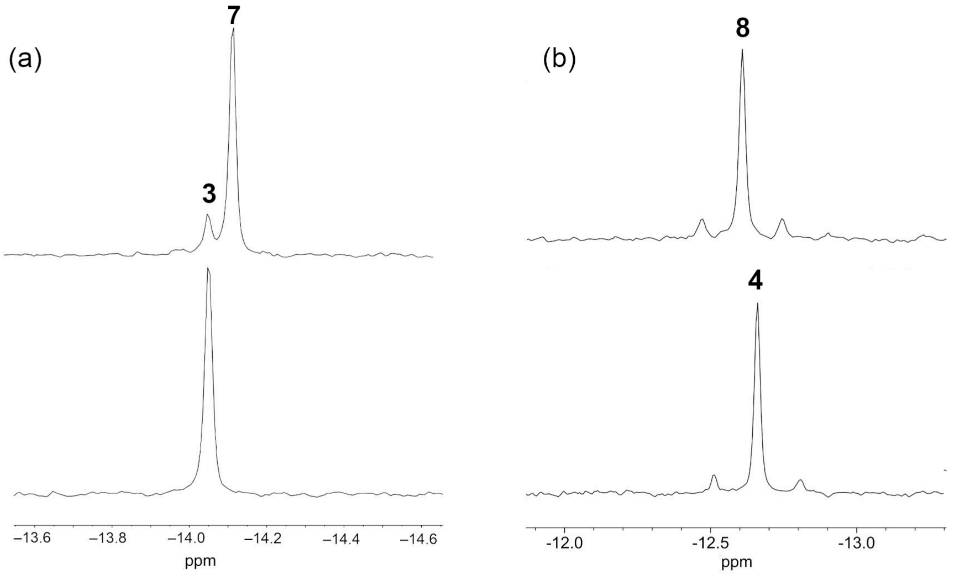

Figure S3 ^{31}P NMR spectra for hydrolysis of $(TBA)_4[(MeO)TiPW_{11}O_{39}]$ $(TBA)_3$**3** to $(TBA)_4[(HO)TiPW_{11}O_{39}]$ $(TBA)_3$**7** (a) and $(TBA)_4[(MeO)SnPW_{11}O_{39}]$ $(TBA)_3$**4** to $(TBA)_4[(HO)SnPW_{11}O_{39}]$ $(TBA)_3$**8** (b) in MeCN.

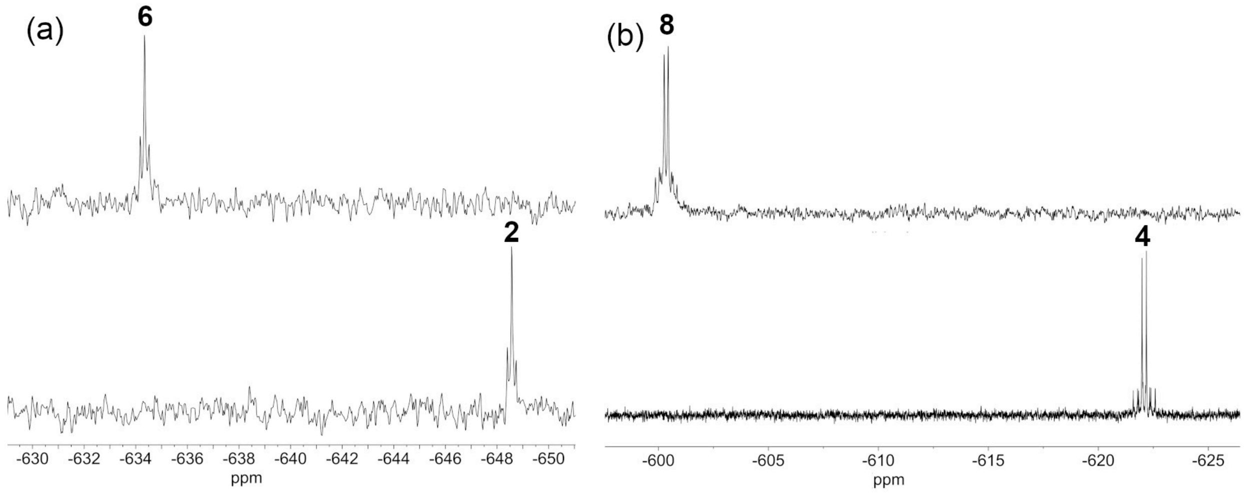

Figure S4 ^{119}Sn NMR spectra for hydrolysis of $(TBA)_3[(MeO)SnW_5O_{18}]$ $(TBA)_3$**2** to $(TBA)_3[(HO)SnW_5O_{18}]$ $(TBA)_3$**6** (a) and $(TBA)_4[(MeO)SnPW_{11}O_{39}]$ $(TBA)_3$**4** to $(TBA)_4[(HO)SnPW_{11}O_{39}]$ $(TBA)_3$**8** (b) in MeCN.

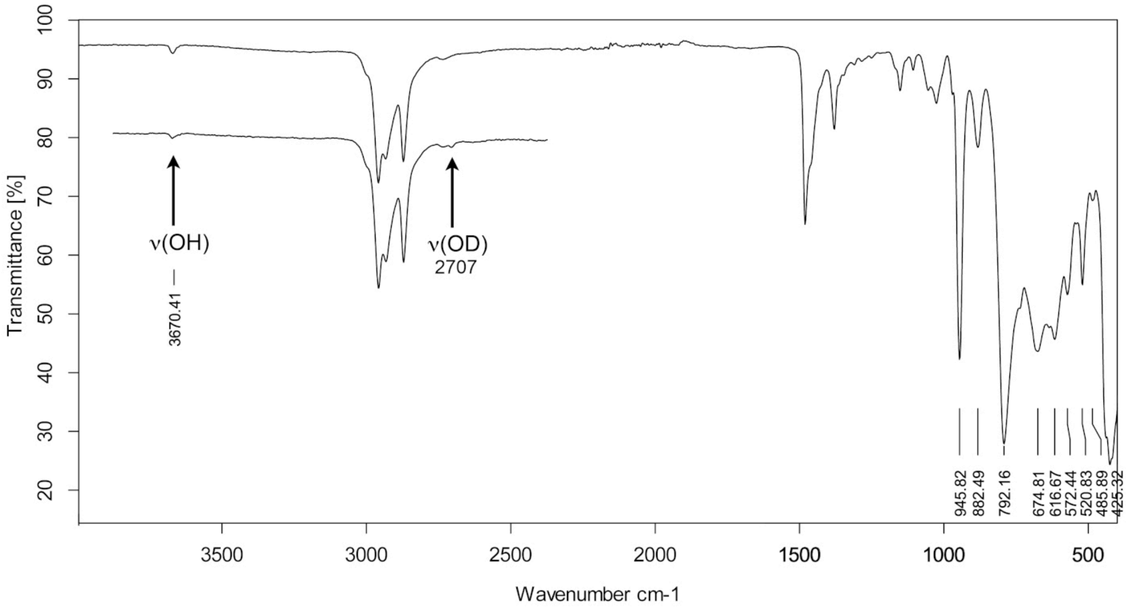

Figure S5 FTIR spectrum of $(TBA)_3[(HO)TiW_5O_{18}]^{3-}$ $(TBA)_3$**5** and part of the spectrum obtained after deuteration.

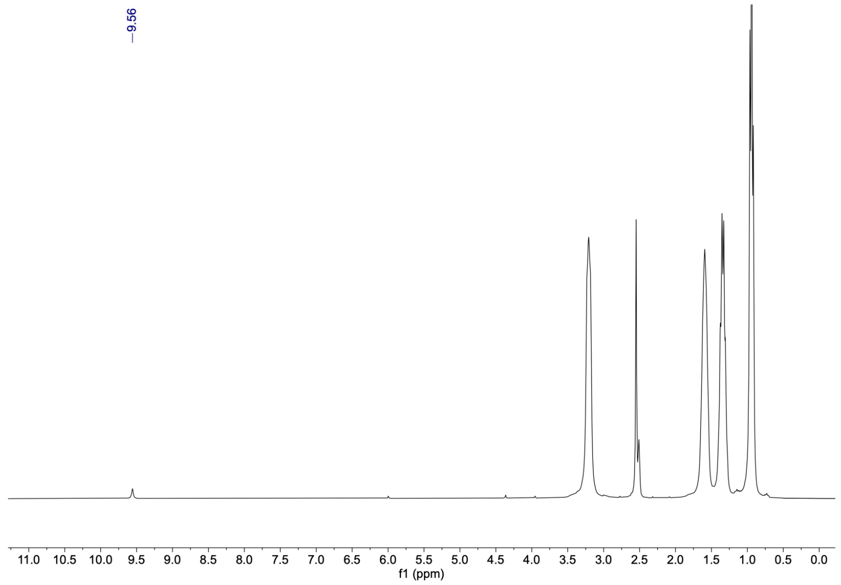

Figure S6 ^{1}H NMR spectrum of $(TBA)_3[(HO)TiW_5O_{18}]$ $(TBA)_3$**5** in d_6-DMSO.

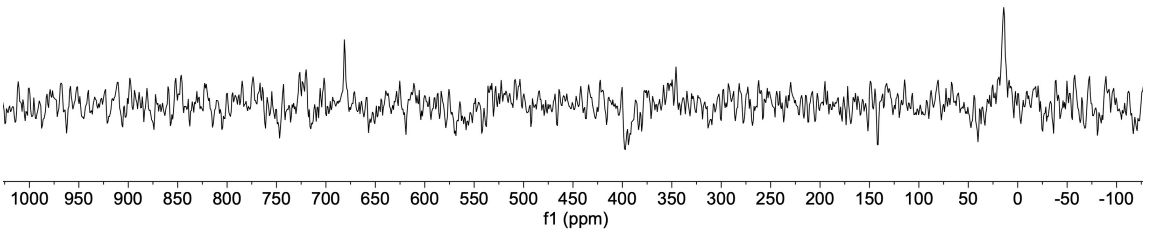

Figure S7 ^{17}O NMR spectrum (in CD_3CN) of the product obtained by treatment of non-^{17}O-enriched $(TBA)_3[(HO)TiW_5O_{18}]$ $(TBA)_3$**5** in DMSO with ^{17}O-enriched H_2O.

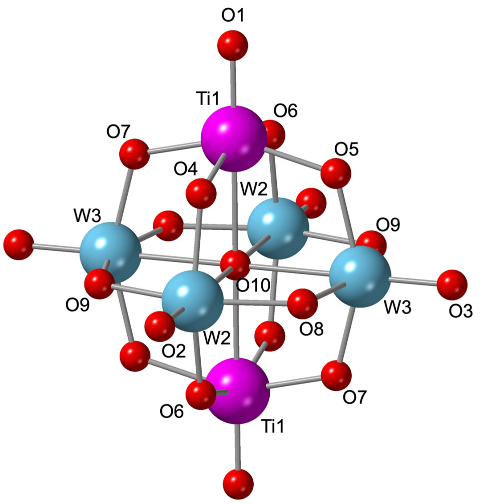

Figure S8 One of the disordered anions in the structure of $(TBA)_3[(HO)TiW_5O_{18}]$ $(TBA)_3$**5** with *trans*-disordered Ti/W sites shown in purple.

Table S7 Crystallographic data for $(TBA)_3[(HO)TiW_5O_{18}]$.MeCN $(TBA)_3$**5**.MeCN

Identification code	rje170001
Empirical formula	$C_{50}H_{111}N_4O_{19}TiW_5$
Formula weight	2039.57
Temperature/K	150.0(2)
Crystal system	monoclinic
Space group	I2/a
a/Å	31.1936(9)
b/Å	18.4834(2)
c/Å	27.5332(10)
α/°	90
β/°	120.793(4)
γ/°	90
Volume/Å^3	13636.7(8)
Z	8
ϱ_{calc}g/cm^3	1.987
μ/mm^{-1}	8.577
F(000)	7864.0
Crystal size/mm^3	0.24 × 0.1 × 0.06
Radiation	MoKα (λ = 0.71073)
2Θ range for data collection/°	6.544 to 56.584
Index ranges	$-39 \leq h \leq 37$, $-24 \leq k \leq 22$, $-35 \leq l \leq 31$
Reflections collected	54968
Independent reflections	15035 [R_{int} = 0.0379, R_{sigma} = 0.0422]
Data/restraints/parameters	15035/793/757
Goodness-of-fit on F^2	1.045
Final R indexes [I>=2σ (I)]	R_1 = 0.0343, wR_2 = 0.0678
Final R indexes [all data]	R_1 = 0.0597, wR_2 = 0.0781
Largest diff. peak/hole / e Å^{-3}	2.61/-2.77

Table S8 Bond distances in the *trans*-disordered anion of $(TBA)_3[(HO)TiW_5O_{18}]$.MeCN $(TBA)_3$**5**

	Bond distance /Å		Bond distance /Å		Bond distance /Å
Ti/W1 – O1	1.720(4)	W2 – O2	1.710(4)	W3 – O3	1.714(4)
Ti/W1 – O4	1.935(4)	W2 – O4	1.911(4)	W3 – O5	1.911(4)
Ti/W1 – O5	1.946(4)	W2 – O6	1.896(4)	W3 – O7	1.915(4)
Ti/W1 – O6	1.944(4)	W2 – O8	1.944(4)	W3 – O8	1.925(4)
Ti/W1 – O7	1.920(4)	W2 – O9	1.932(4)	W3 – O9	1.938(4)
Ti/W1 – O10	2.3245(3)	W2 – O10	2.3160(3)	W3 – O10	2.3247(4)

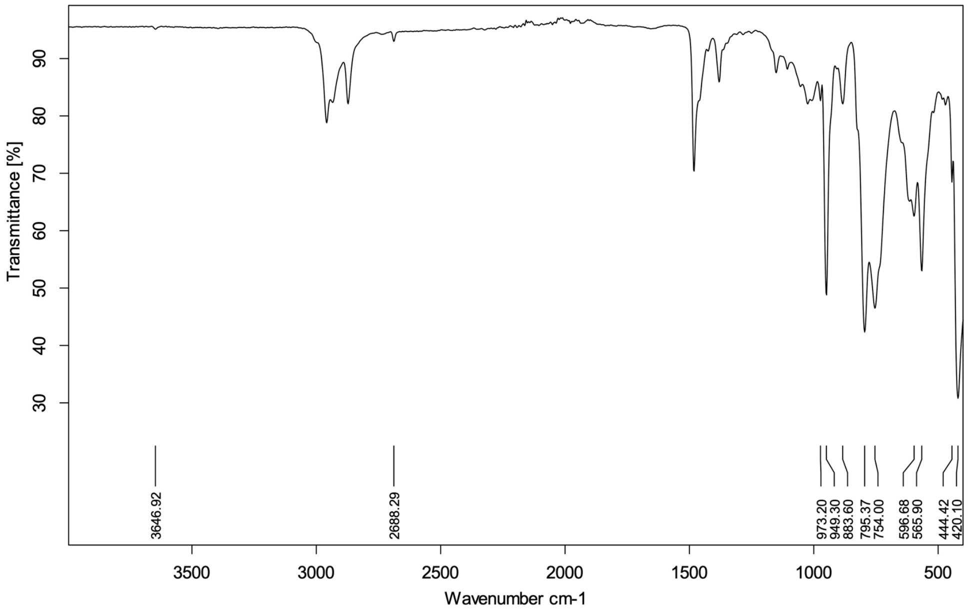

Figure S9 FTIR spectrum of incompletely deuteriated $(TBA)_3[(HO)SnW_5O_{18}]$ $(TBA)_3$**6**.

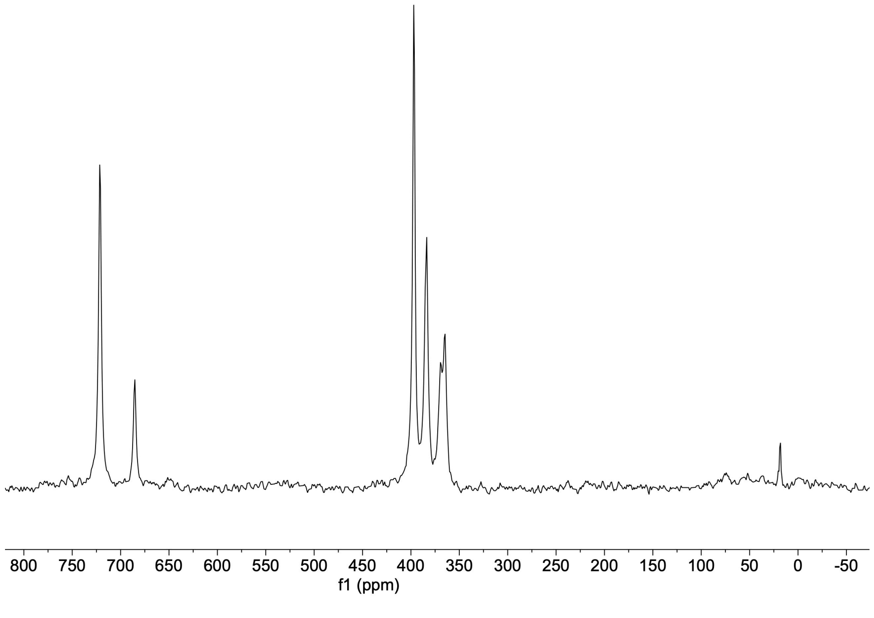

Figure S10 ^{17}O NMR spectrum of $(TBA)_3[(HO)SnW_5O_{18}]$ $(TBA)_6$**6** in MeCN.

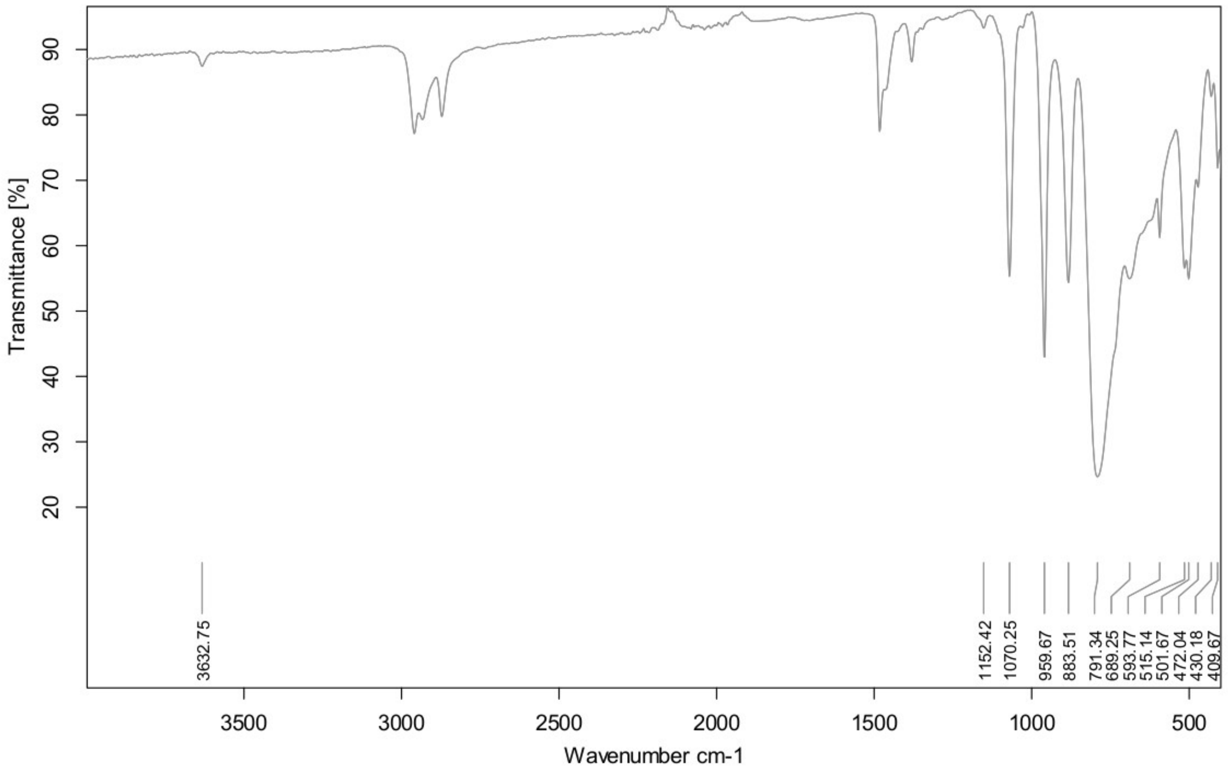

Figure S11 FTIR spectrum of $(TBA)_4[(HO)TiPW_{11}O_{39}]$ $(TBA)_4$**7**

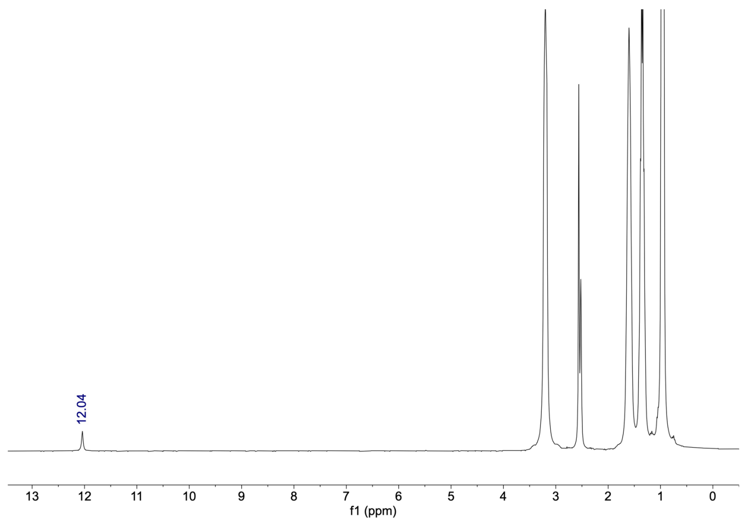

Figure S12 ^{1}H NMR spectrum of $(TBA)_4[(HO)TiPW_{11}O_{39}]$ $(TBA)_4$**7** in $(CD_3)_2SO$.

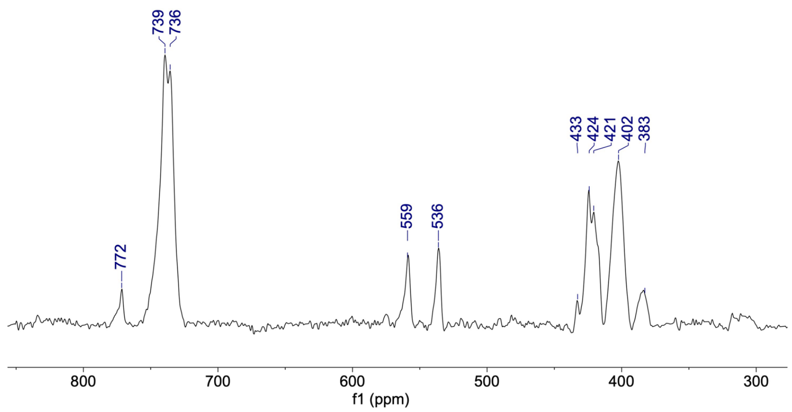

Figure S13 ^{17}O NMR spectrum of $(TBA)_4[(HO)TiPW_{11}O_{39}]$ $(TBA)_4$**7** in CD_3CN.

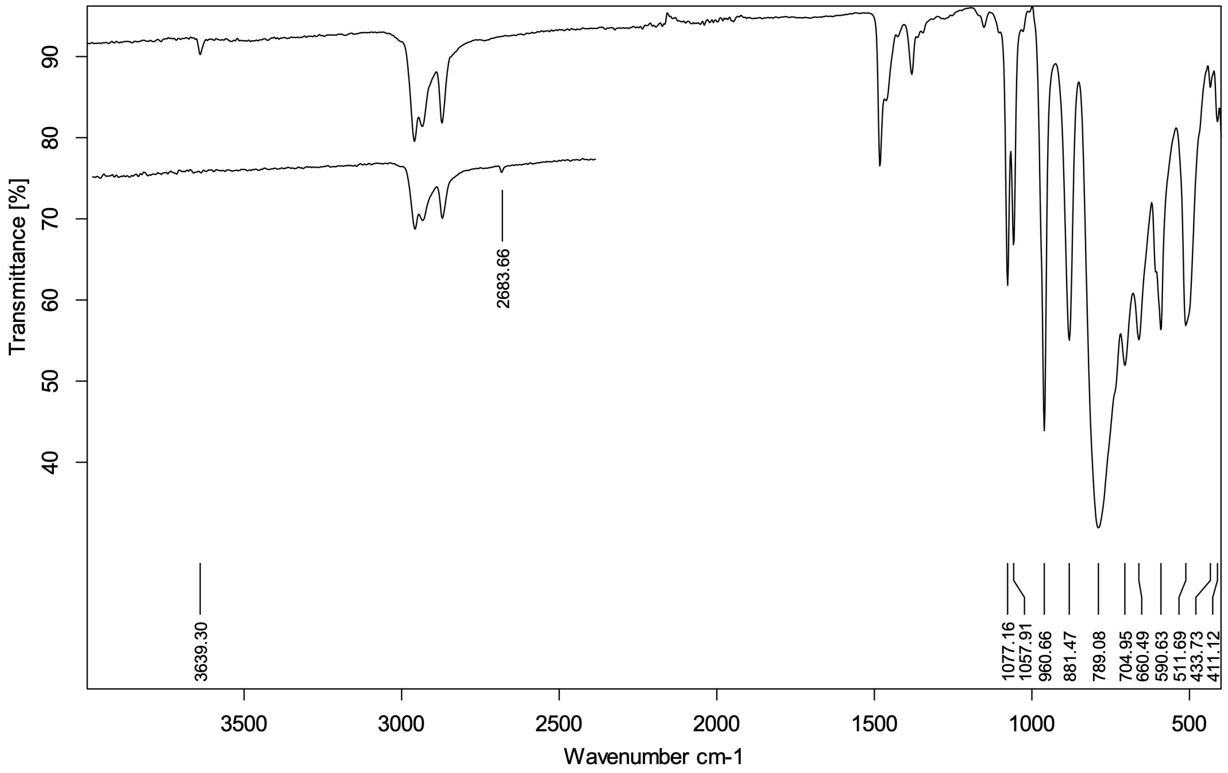

Figure S14 FTIR spectrum of $(TBA)_4[(HO)SnPW_{11}O_{39}]$ $(TBA)_4$**8** and part of the spectrum after deuteriation.

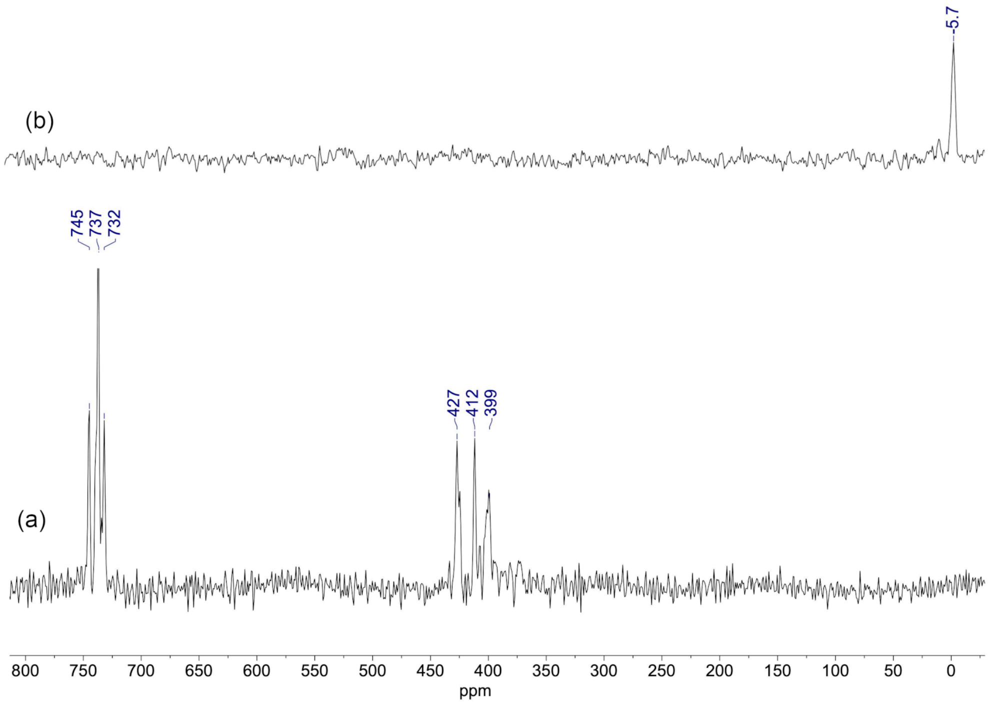

Figure S15 ^{17}O NMR spectra of (a) $(TBA)_4[(HO)SnPW_{11}O_{39}]$ $(TBA)_4$**8** with ^{17}O-enriched $\{SnPW_{11}O_{39}\}$ and (b) product from treatment of non-enriched] $(TBA)_4$**8** with ^{17}O-enriched H_2O.

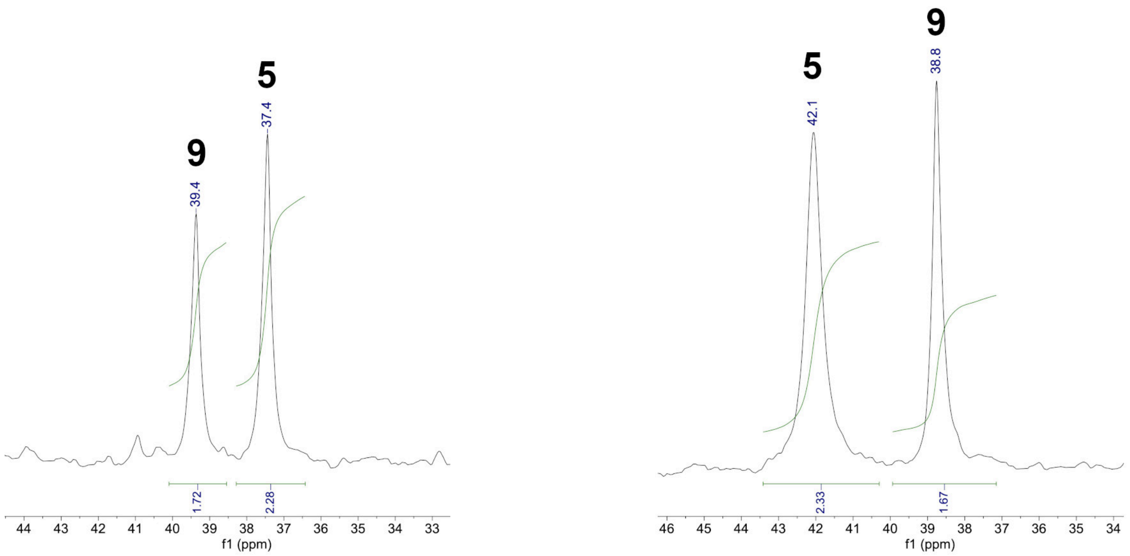

Figure S16 ^{183}W NMR equilibrium analysis for the condensation of $(TBA)_3[(HO)TiW_5O_{18}]$ $(TBA)_3$**5** to $(TBA)_6[(\mu\text{-}O)(TiW_5O_{18})_2]$ $(TBA)_6$**9** , showing W_{eq} peaks in MeCN (left) and DMSO (right).

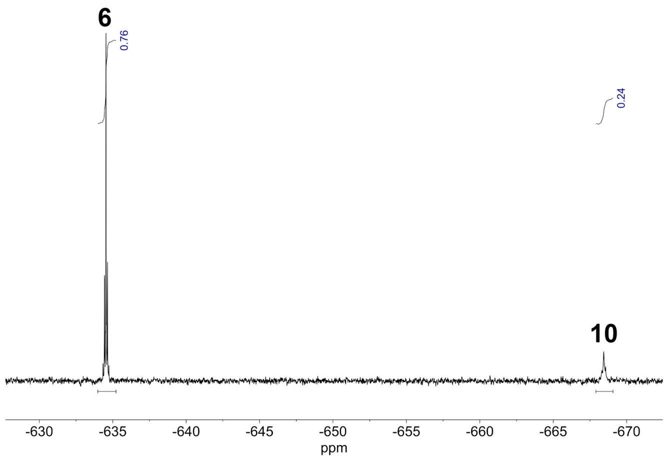

Figure S17 ^{119}Sn NMR equilibrium analysis for the condensation of $(TBA)_3[(HO)SnW_5O_{18}]$ $(TBA)_3$**6** to $(TBA)_6[(\mu\text{-}O)(SnW_5O_{18})_2]$ $(TBA)_6$**10**.

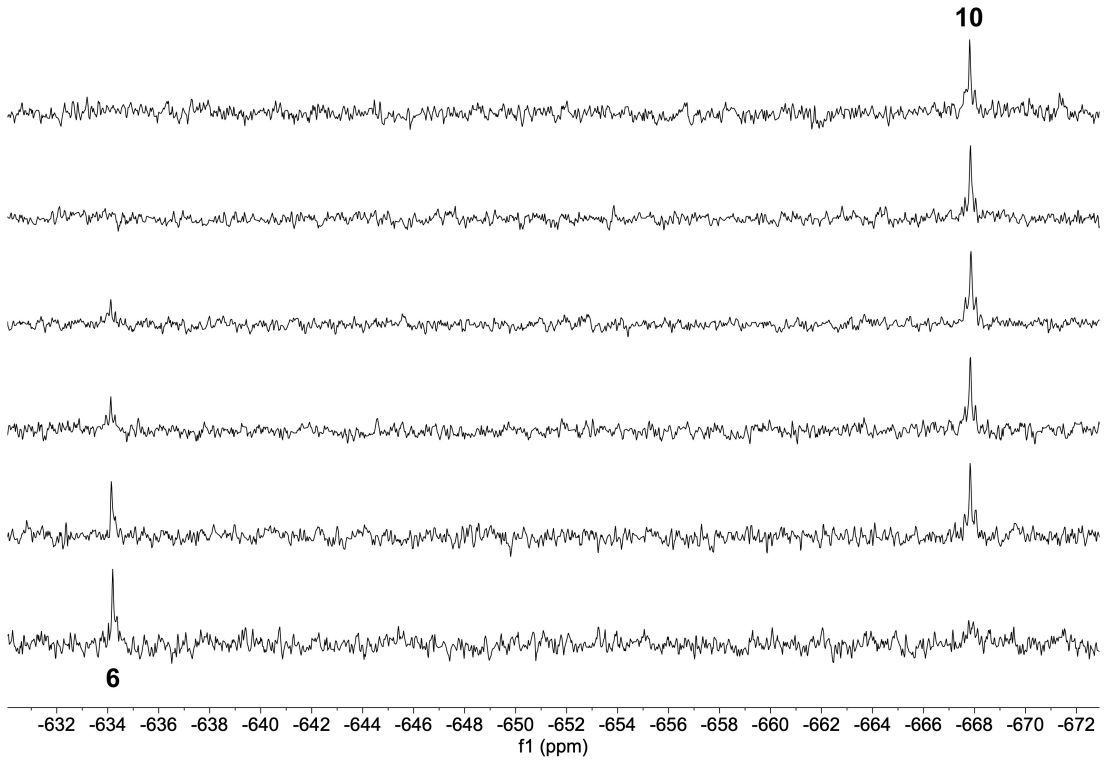

Figure S18 ^{119}Sn NMR study of $[(HO)SnW_5O_{18}]^{3-}$ **6** conversion to $[(\mu\text{-}O)(SnW_5O_{18})_2]^{6-}$ **10** in hot PhCN with periodic removal of H_2O under reduced pressure.

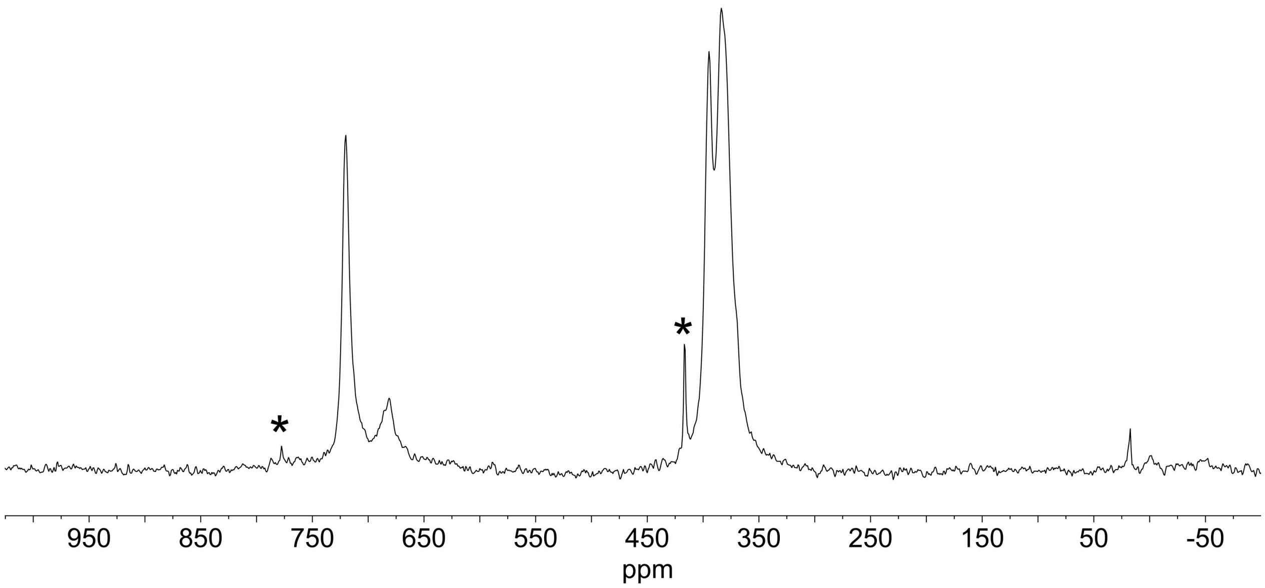

Figure S19 ^{17}O NMR spectrum of $(TBA)_6[(\mu\text{-}O)(SnW_5O_{18})_2]$ $(TBA)_6$**10**. Peaks marked with an asterisk are due to $(TBA)_2[W_6O_{19}]$ impurity.

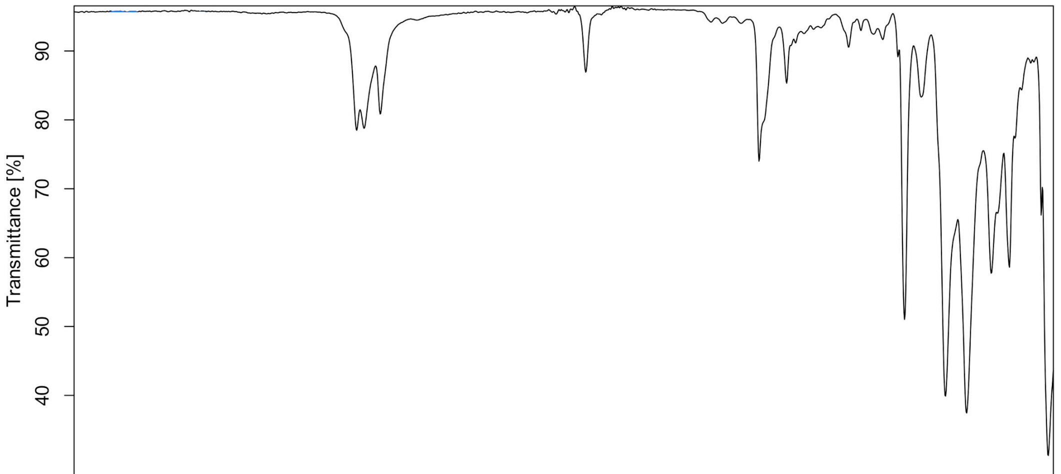

Figure S20 FTIR spectrum of $(TBA)_6[(\mu\text{-}O)(SnW_5O_{18})_2]$ $(TBA)_6$**10**. The band at 2117cm^{-1} is due to *N, N'*-dicyclohexylcarbodiimide (DCC) impurity.

Table S9 Crystallographic data for $(TBA)_9[(\mu\text{-}O)(SnW_5O_{18})_2].[(HO)SnW_5O_{18}].3MeCN$.

Identification code	rje230098_fa
Empirical formula	$C_{86}H_{189}N_8O_{56}Sn_3W_{15}$
Formula weight	5345.26
Temperature/K	150.0(2)
Crystal system	monoclinic
Space group	$P2_1/c$
a/Å	26.5593(5)
b/Å	21.3806(4)
c/Å	37.6554(6)
α/°	90
β/°	94.4533(17)
γ/°	90
Volume/Å^3	21318.1(7)
Z	4
ρ_{calc}g/cm^3	1.665
μ/mm^{-1}	17.667
F(000)	9876.0
Crystal size/mm^3	0.24 × 0.11 × 0.08
Radiation	CuKα (λ = 1.54184)
2Θ range for data collection/°	4.708 to 155.946
Index ranges	$-32 \le h \le 32$, $-13 \le k \le 26$, $-47 \le l \le 45$
Reflections collected	159121
Independent reflections	41152 [R_{int} = 0.0656, R_{sigma} = 0.0490]
Data/restraints/parameters	41152/2724/1701
Goodness-of-fit on F^2	1.076
Final R indexes [I>=2σ (I)]	R_1 = 0.0756, wR_2 = 0.1965
Final R indexes [all data]	R_1 = 0.0997, wR_2 = 0.2148
Largest diff. peak/hole / e Å^{-3}	3.46/-3.38

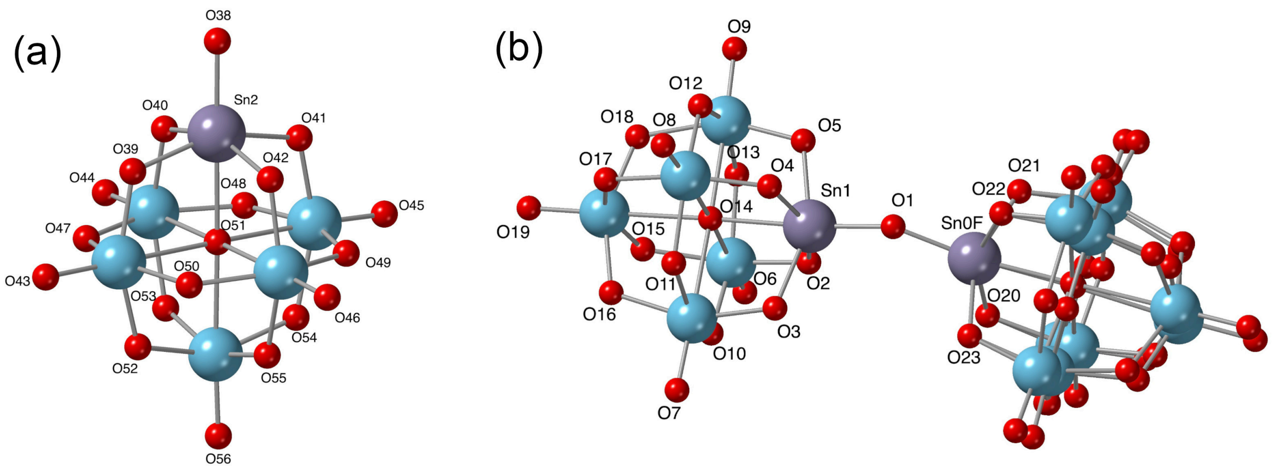

Figure S21 Structures of the anions **6** (a) and disordered **10** (b) present in the co-crystalline $(TBA)_9[(\mu\text{-}O)(SnW_5O_{18})_2].[(HO)SnW_5O_{18}].3MeCN$.

Table S10. Selected bond lengths in the anion $[(HO)SnW_5O_{18}]^{3-}$ **6** from the structure of $(TBA)_9[(\mu\text{-}O)(SnW_5O_{18})_2].[(HO)SnW_5O_{18}].3MeCN$.

	Bond distance /Å
Sn2 – O38	1.938(16)
Sn2 – O39	2.027(12)
Sn2 – O40	1.998(11)
Sn2 – O41	2.035(13)
Sn2 – O42	1.989(11)
Sn2 – O51	2.276(9)

Table S11. Selected bond lengths and angle in the anion $[(\mu\text{-}O)(SnW_5O_{18})_2]^{6-}$ **10** from the structure of $(TBA)_9[(\mu\text{-}O)(SnW_5O_{18})_2].[(HO)SnW_5O_{18}].3MeCN$.

	Bond distance /Å		Bond distance /Å
Sn1 – O1	1.922(12)	Sn0F – O1	1.898(12)
Sn1 – O2	2.039(10)	Sn0F – O20	1.979(11)
Sn1 – O3	2.041(10	Sn0F – O21	2.027(10)
Sn1 – O4	2.057(10)	Sn0F – O22	2.006(11)
Sn1 – O5	2.038(10)	Sn0F – O23	2.011(11)
Sn1 – O14	2.242(8)		
	**Bond angle / °**		
Sn1OSn0F	151.4(7)		

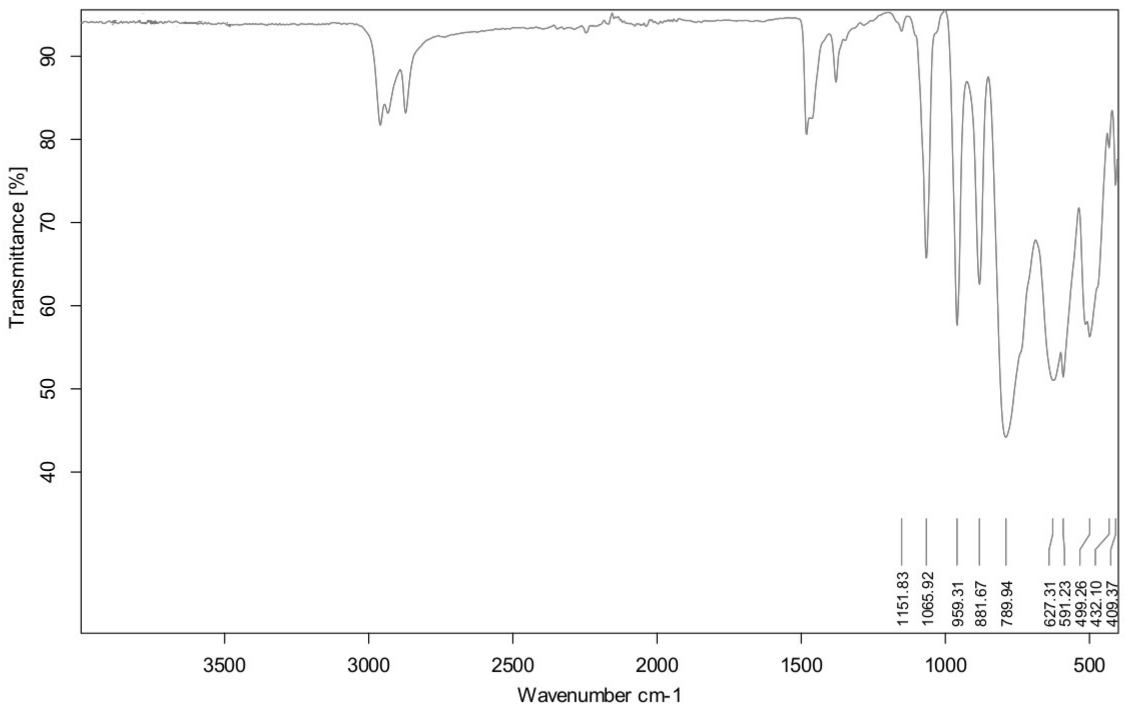

Figure S22 FTIR spectrum of $(TBA)_8[(\mu\text{-}O)(TiPW_{11}O_{39})_2]$ (TBA)$_8$**11**

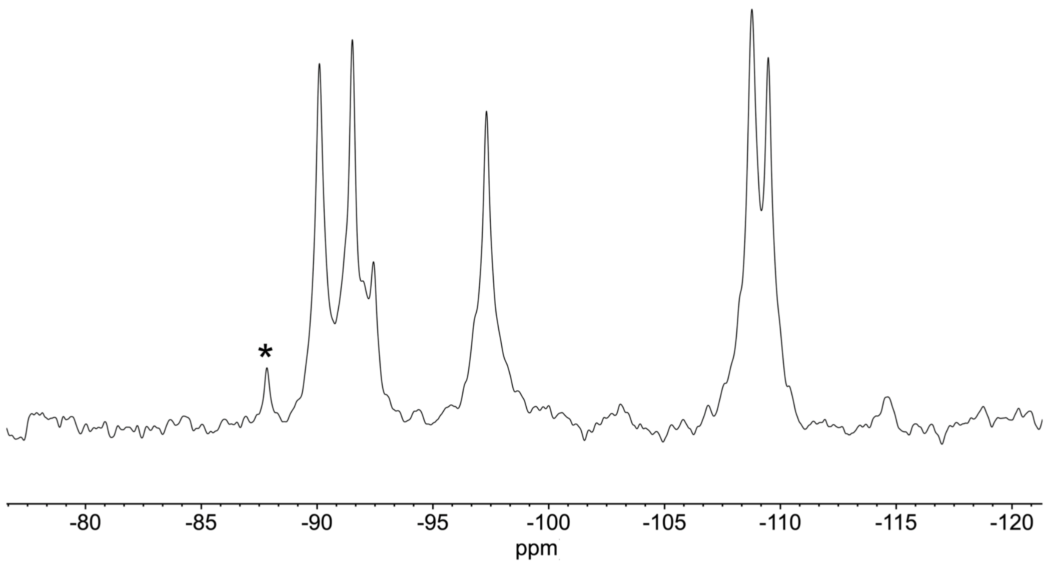

Figure S23 ^{183}W NMR spectrum of $(TBA)_8[(\mu\text{-}O)(TiPW_{11}O_{39})_2]$ (TBA)$_8$**11** in MeCN. The peak marked with an asterisk is assigned to $[PW_{12}O_{40}]^{3-}$.

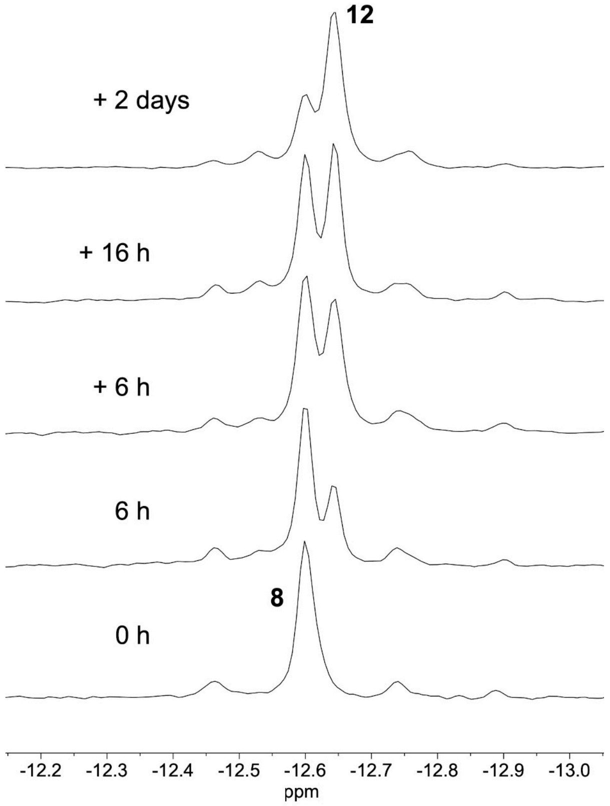

Figure S24 ^{31}P NMR study of $[(HO)SnPW_{11}O_{39}]^{4-}$ **8** thermal condensation to $[(\mu\text{-}O)(SnPW_{11}O_{39})_2]^{8-}$ **12** by successive periods of heating at 120 °C for the times indicated with removal of water after each stage.

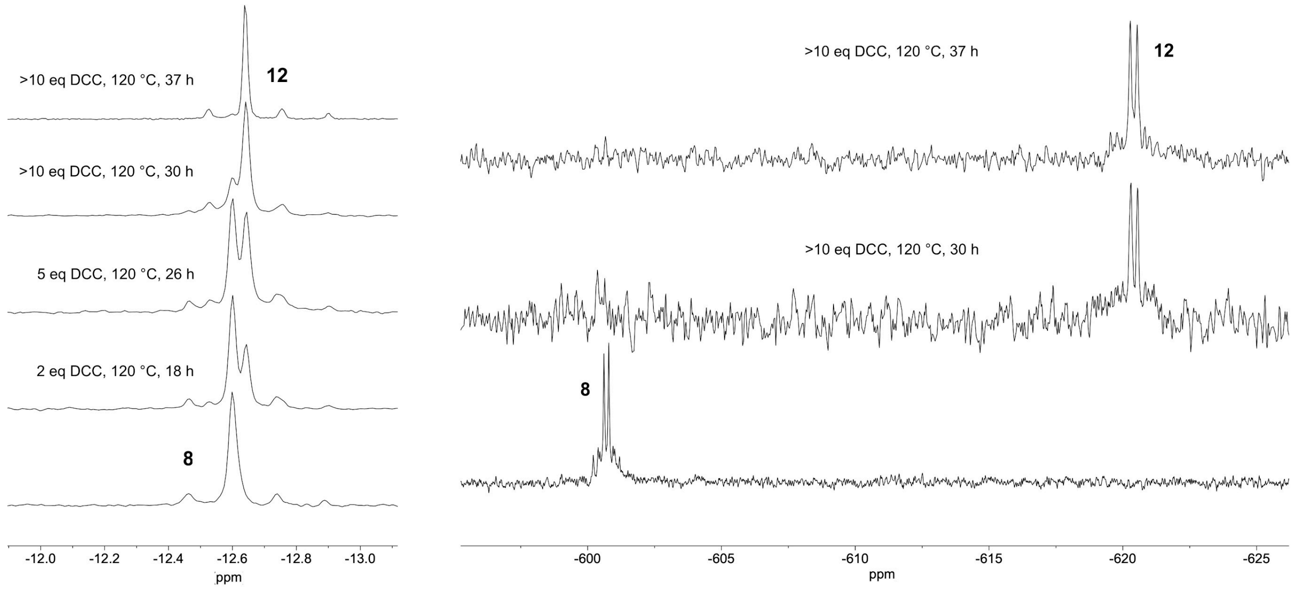

Figure S25 ^{31}P (left) and ^{119}Sn (right) NMR studies of $(TBA)_4[(HO)SnPW_{11}O_{39}]$ $(TBA)_4$**8** condensation to $(TBA)_8[(\mu\text{-}O)(SnPW_{11}O_{39})_2]$ $(TBA)_4$**12** with DCC as dehydrating agent.

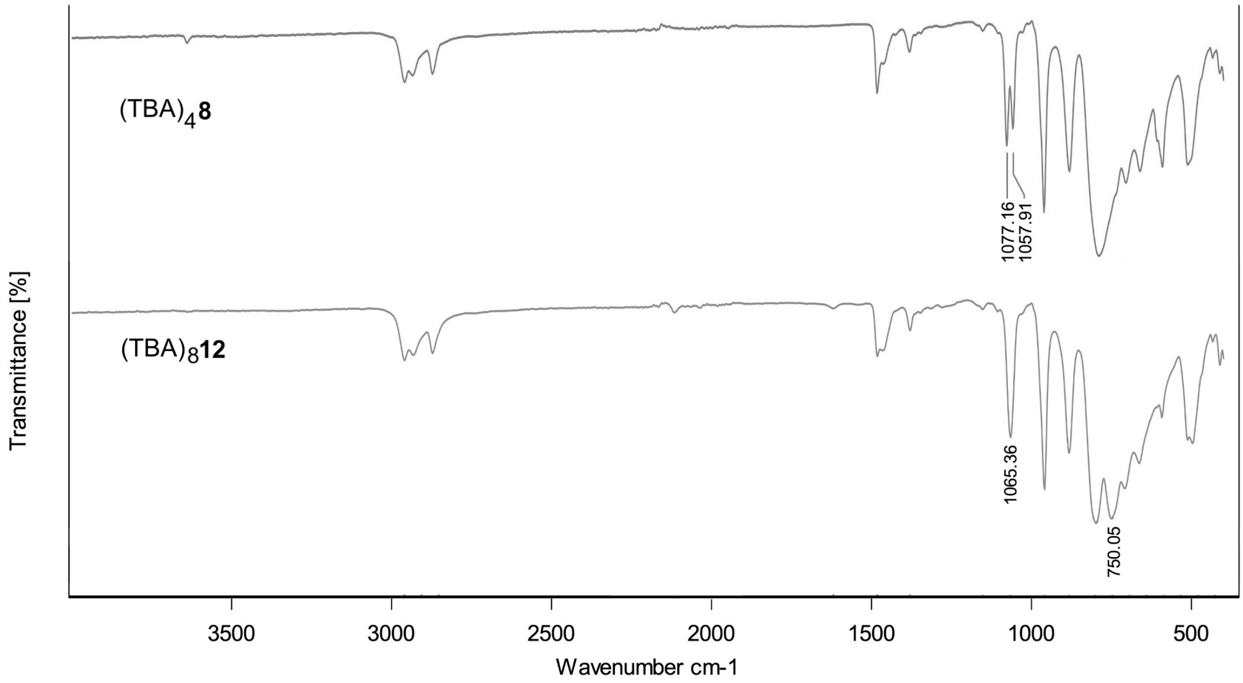

Figure S26 FTIR spectrum of $(TBA)_8[(\mu\text{-}O)(SnPW_{11}O_{39})_2]$ $(TBA)_8$**12** compared with that of $(TBA)_4[(HO)SnPW_{11}O_{39}]$ $(TBA)_4$**8**.

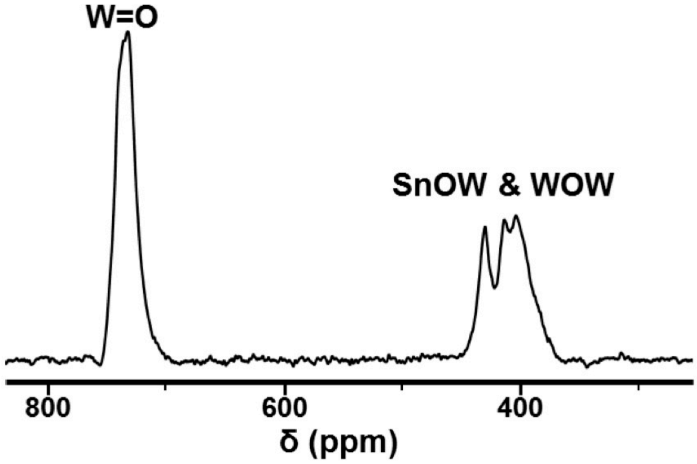

Figure S27 ^{17}O NMR spectrum of $(TBA)_8[(\mu\text{-}O)(SnPW_{11}O_{39})_2]$ $(TBA)_8$**12**

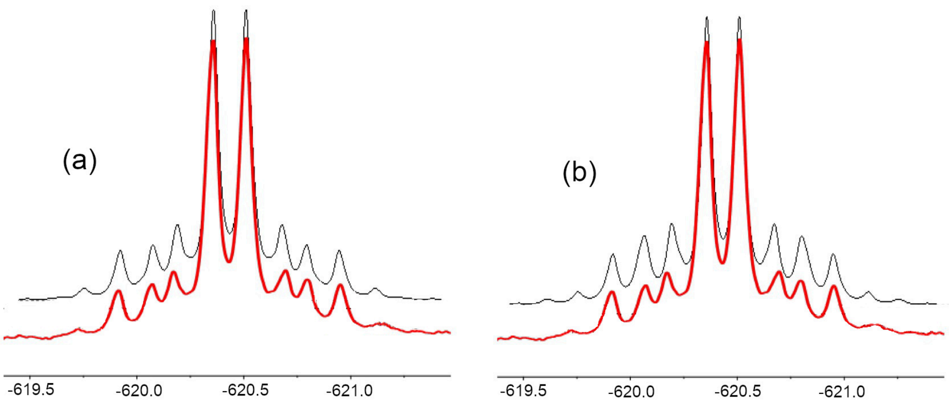

Figure S28 Observed (lower) and simulated (upper) ^{119}Sn NMR spectra of $(TBA)_8[(\mu\text{-}O)(SnPW_{11}O_{39})_2]$ $(TBA)_8$**12** using $^{2}J(^{119}Sn^{117}Sn)$ values of 60 Hz (a) or 110 Hz (b) for simulation.

Table S12. Kinetic parameters from analysis of NMR data. *K* denotes the equilibrium constant, k_f the forward second order rate constant and k_b the backward rate constant.

Reaction	*K*	$k_f/M^{-1}s^{-1}$	$k_b/M^{-1}s^{-1}$	ΔG° @ 298K / kJ mol^{-1}
**MeOH exchgange**[a]				
{MeOSnW$_5$} + 7.3 MeOH	1	$2.9\cdot10^{-1}$	$2.9\cdot10^{-1}$	0
{MeOSnPW$_{11}$} + 25 MeOH	1	$1.2\cdot10^{-1}$	$1.2\cdot10^{-1}$	0
**CD_3OD exchange**				
{MeOTiW$_5$} + 3.9 CD_3OD	$3.3\cdot10^{-1}$	$2.0\cdot10^{-2}$	$6.1\cdot10^{-2}$	2.78
{MeOTiPW$_{11}$} + 9 CD_3OD	$4.2\cdot10^{-1}$	$4.1\cdot10^{-4}$	$9.8\cdot10^{-4}$	2.14
{MeOSnPW$_{11}$} + 9.5 CD_3OD	$4\cdot10^{-1}$	$3\cdot10^{-2}$	$8\cdot10^{-2}$	2.13
**Hydrolysis**				
{MeOTiW$_5$} + 2.2 H_2O (CD_3CN)	$6.0\cdot10^{-2}$	$5.0\cdot10^{-3}$	$8.3\cdot10^{-2}$	6.97
{MeOTiW$_5$} + 2.2 H_2O (DMSO)	$1\cdot10^{-1}$	$2\cdot10^{-2}$	$1\cdot10^{-1}$	4.72
{MeOTiW$_5$} + 44 H_2O (CD_3CN)	$8.4\cdot10^{-2}$	$6.0\cdot10^{-4}$	$7.2\cdot10^{-3}$	6.15
{MeOTiW$_5$} + 44 H_2O (DMSO)	$8.3\cdot10^{-2}$	$4.2\cdot10^{-4}$	$5.0\cdot10^{-3}$	6.16
{MeOTiPW$_{11}$} + 7.5 H_2O (CD_3CN)	$2.0\cdot10^{-2}$	$5.8\cdot10^{-5}$	$2.8\cdot10^{-3}$	9.64
{MeOTiPW$_{11}$} + 9.2 H_2O (DMSO)	1.0	$1.2\cdot10^{-3}$	$1.2\cdot10^{-3}$	0.00
{MeOTiPW$_{11}$} + 50 H_2O (CD_3CN)	$2.6\cdot10^{-1}$	$2.4\cdot10^{-5}$	$9.3\cdot10^{-5}$	3.32
{MeOSnPW$_{11}$} + 4 H_2O (CD_3CN)	$8\cdot10^{-1}$	$8\cdot10^{-2}$	$1\cdot10^{-1}$	$7.06\cdot10^{-1}$
**Condensation**				
2 {HOTiPW$_{11}$} $\rightleftharpoons$ ((μ-O)(TiPW$_{11}$)$_2$}	4.6	$6.5\cdot10^{-4}$	$1.41\cdot10^{-4}$	–3.78

[a] Values obtained from analysis of 2D EXSY ^{1}H NMR

DFT results

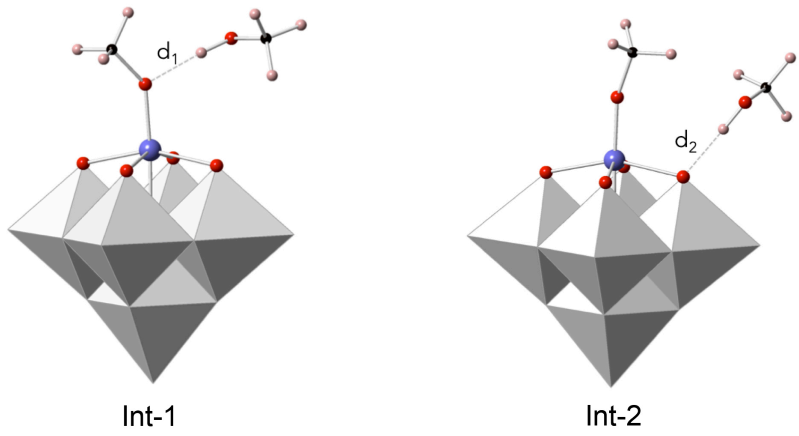

Figure S29 Polyhedral representation of **Int-1** and **Int-2** in MOMe/MeOH exchange for anions **1** and **2**. Grey polyhedra W; blue M (Ti or Sn); red O; black C; pink H.

Table S13. Relative energies with respect to reactants (kcal·mol^{-1}) and distances (Å) for **Int-1** and **Int-2** in MOMe/MeOH exchange for anions **1** and **2**.

Anion	E_{int-1}	d_1(O-H)	E_{int-2}	d_2(O-H)	$\Delta E_{int2-int1}$
$[(MeO)TiW_5O_{18}]^{3-}$ **1**	–6.14	1.87	–8.41	1.80	–2.27
$[(MeO)SnW_5O_{18}]^{3-}$ **2**	−7.05	1.78	−6.54	1.79	0.50

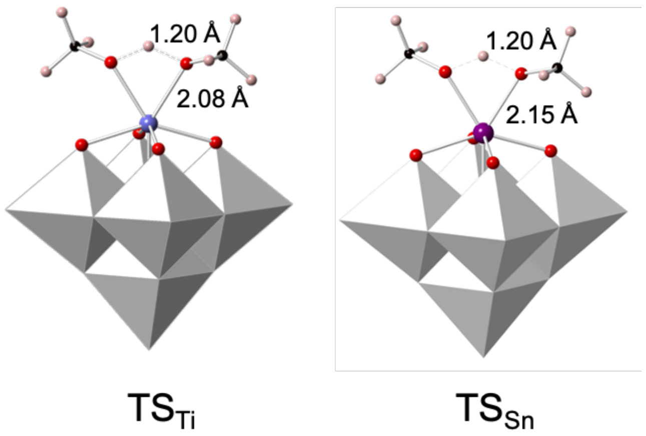

Figure S30 Computed structures of the most favorable transition states for MeOH exchange with {TiW_5} methoxido anion **1** and {SnW_5} methoxido anion **2**.

Table S14. Relative energies with respect to reactants (kcal.mol^{-1}) for transition states and products hydrolysis of methoxido Lindqvist anions $[(MeO)TiW_5O_{18}]^{3-}$ **1**, $[(MeO)SnW_5O_{18}]^{3-}$ **2** and $[(MeO)NbW_5O_{18}]^{2-}$. Relative Gibbs free energies (kcal.mol^{-1}) are in parentheses.

**Reactant**	$E_{rel.hyd}$ **TS1**	$E_{rel.hyd}$ **Product**
$[(MeO)TiW_5O_{18}]^{3-}$ **1**	15.8 (26.4)	1.1 (0.3)
$[(MeO)SnW_5O_{18}]^{3-}$ **2**	9.8 (20.2)	-3.1 (-4.0)
$[(MeO)NbW_5O_{18}]^{2-}$	8.7 (19.8)	2.9 (2.1)

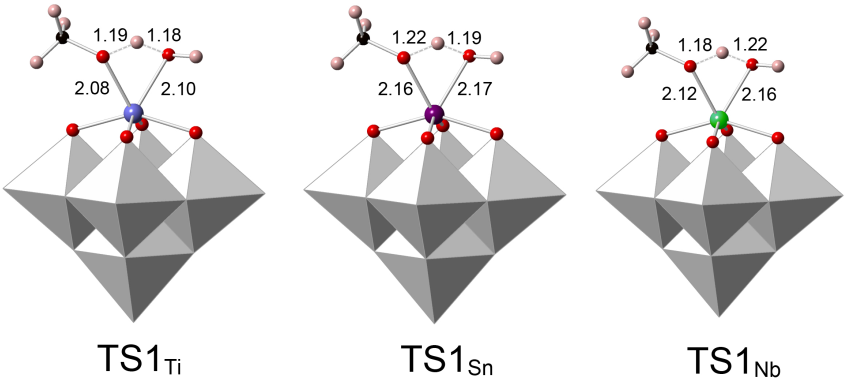

Figure S31 Optimized transition state structures for hydrolysis of **1** ($TS1_{Ti}$), **2** ($TS1_{Sn}$) and $[(MeO)NbW_5O_{18}]^{2-}$ ($TS1_{Nb}$) . Grey polyhedra W; red O; blue Ti; purple Sn; green Nb; black C; pink H. Distances in Å.

Table S15 Relative energies with respect to reactants (kcal.mol^{-1}) for transition states and products for condensation of hydroxido anions **5**, **6** and $[(HO)NbW_5O_{18}]^{2-}$. Relative Gibbs free energies (kcal.mol^{-1}) are in parentheses.

<table>
<tr><th>Reactant</th><th>$E_{rel.}$ $TS2_1$</th><th>$E_{rel.}$ $TS2_2$</th><th>$E_{rel.cond}$ Product</th></tr>
<tr><td>$[(HO)TiW_5O_{18}]^{3-}$ 5</td><td colspan="2">21.1 (38.8)</td><td>3.6 (7.6)</td></tr>
<tr><td>$[(HO)SnW_5O_{18}]^{3-}$ 6</td><td>12.7 (29.6)</td><td>20.8 (40.6)</td><td>9.0 (12.1)</td></tr>
<tr><td>$[(HO)NbW_5O_{18}]^{2-}$</td><td colspan="2">13.3 (31.2)</td><td>2.5 (3.9)</td></tr>
</table>

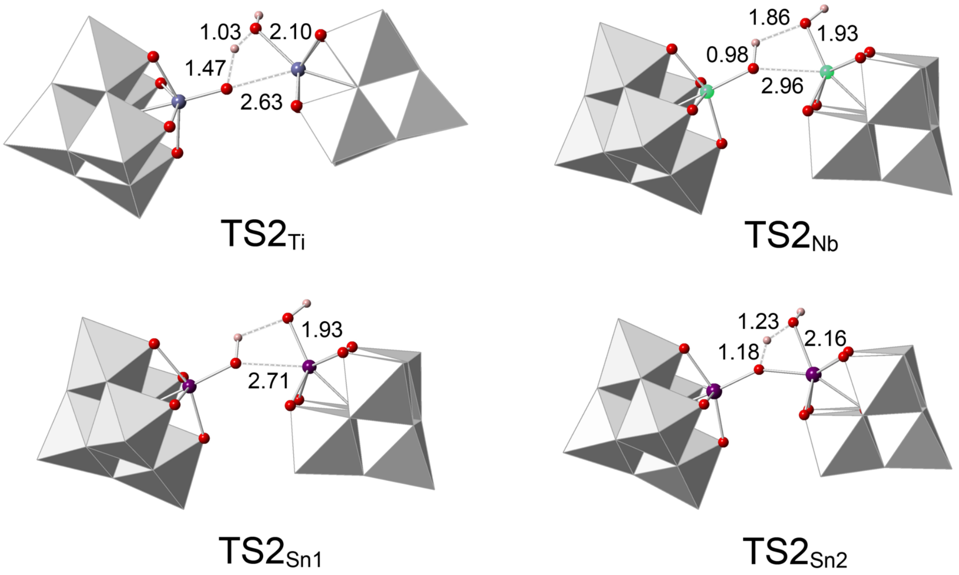

Figure S32 Optimized transition state structures for the formation of $[(\mu\text{-O})(TiW_5O_{18})_2]^{6-}$ **9** ($TS2_{Ti}$), $[(\mu\text{-O})(NbW_5)_2]^{4-}$ ($TS2_{Nb}$) and $[(\mu\text{-O})(SnW_5O_{18})_2]^{6-}$ **10** ($TS2_{Sn1}$ and $TS2_{Sn2}$). Grey polyhedra: W; red: O; blue: Ti; green: Nb; purple: Sn; pink: H. Distances in Å.

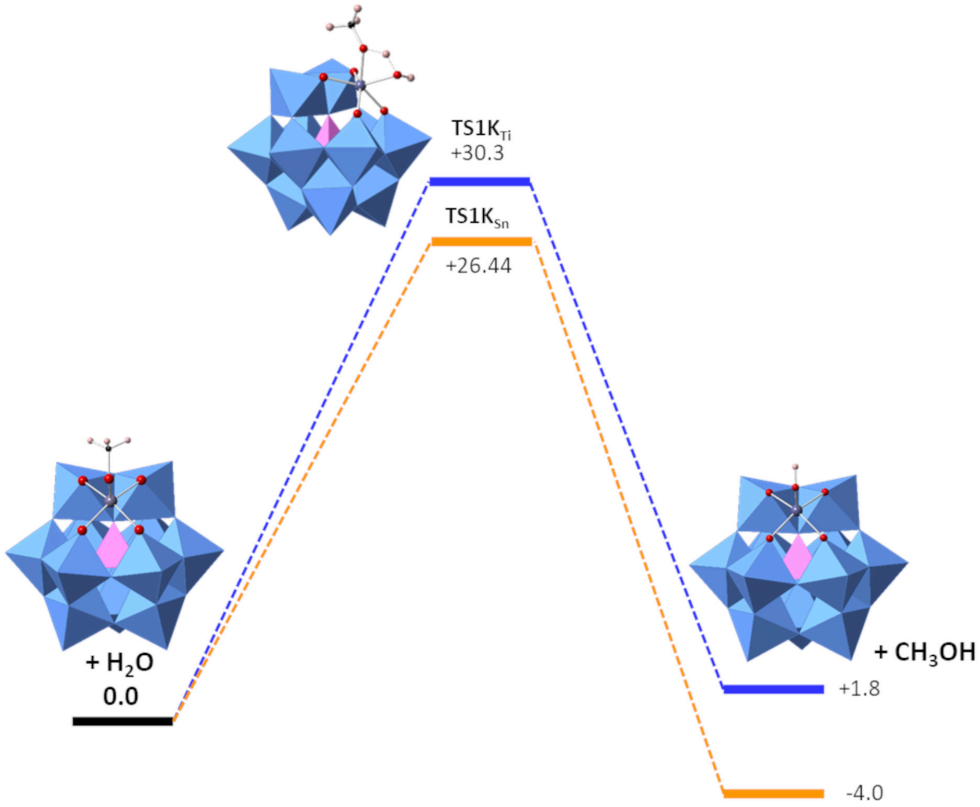

Figure S33 Energy profiles for hydrolysis of methoxido Keggin anions **5** (blue) and **6** (red).

Table S16 Relative energies with respect to reactants (kcal.mol^{-1}) of the transition states and products for hydrolysis of methoxido Keggin anions 3 and 4. Relative Gibbs free energies (kcal.mol^{-1}) are in parenthesis.

Reactant	$E_{rel.hyd}$ TS1	$E_{rel.hyd}$ Product
$[(MeO)TiPW_{11}O_{39}]^{4-}$ **3**	19.0 (30.3)	2.1 (1.8)
$[(MeO)SnPW_{11}O_{39}]^{4-}$ **4**	8.8 (26.4)	-2.7 (-4.0)

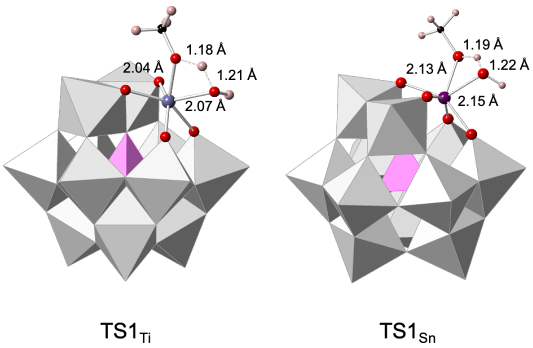

Figure S34 Optimized transition state structures in the formation of $[(HO)TiPW_{11}O_{39}]^{4-}$ **7** ($TS1_{Ti}$) and $[(HO)SnPW_{11}O_{39}]^{4-}$ **8** ($TS1_{Sn}$). Color code: Grey polyhedra-W, pink polyhedra-P, red-O, purple-Ti or Sn, black-C and pink-H..

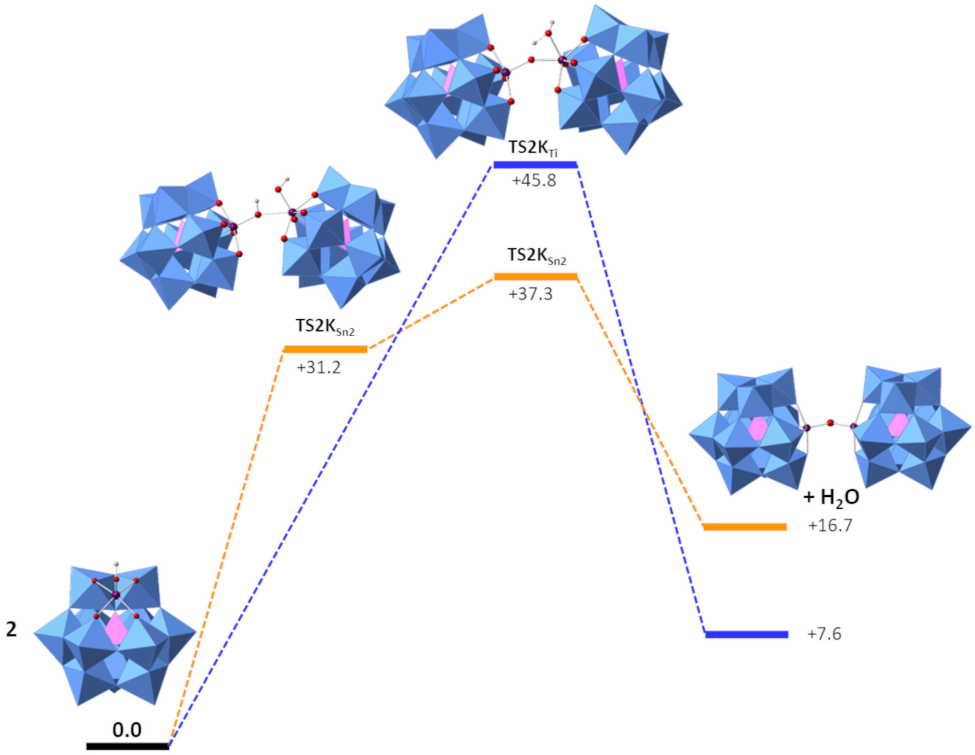

Figure S35 Energy profiles for condensation of hydroxides $[(HO)TiPW_{11}O_{39}]^{4-}$ **7** (blue) and $[(HO)SnPW_{11}O_{39}]^{4-}$ **8** (red). Relative Gibbs free energies (kcal·mol^{-1}) are in parentheses.

Table S17 Relative energies with respect to reactants ($kcal.mol^{-1}$) of transition states and products for condensation of hydroxido {MPW_{11}} anions **7** and **8**. Relative Gibbs free energies ($kcal.mol^{-1}$) are in parenthesis.

Reactant	$E_{rel.}$ $TS2_1$	$E_{rel.}$ $TS2_2$	$E_{rel.cond.}$ Product
$[(HO)TiPW_{11}O_{39}]^{4-}$ **7**	22.9 (45.8)		3.6 (7.6)
$[(HO)SnPW_{11}O_{39}]^{4-}$ **8**	15.4 (31.2)	23.8 (37.3)	12.9 (16.7)

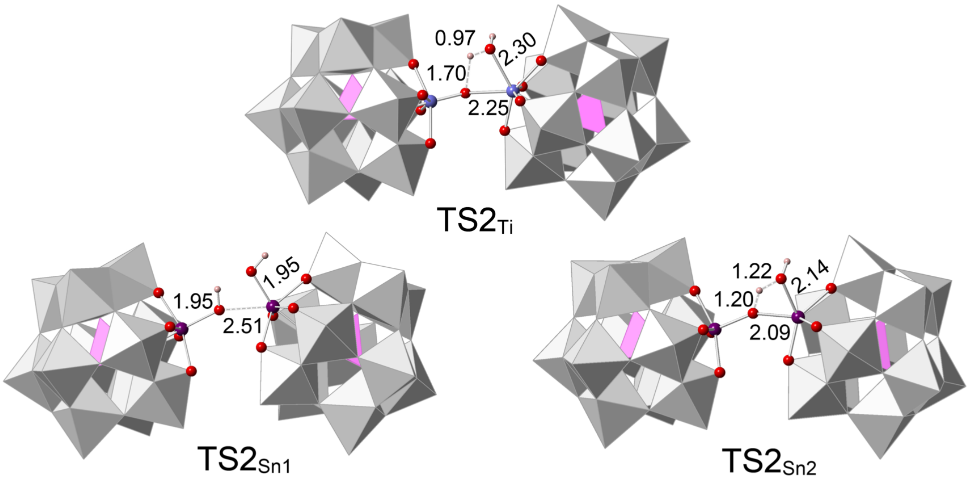

Figure S36 Optimized transition state structures for the formation of $[(\mu\text{-}O)(TiPW_{11}O_{39})_2]^{8-}$ **11** ($TS2_{Ti}$) and $[(\mu\text{-}O)(SnPW_{11}O_{39})_2]^{8-}$ **12** ($TS2_{1Sn}$ and $TS2_{2Sn}$). Color code: Grey polyhedra-W pink polyhedra-P, red-O, purple-Ti or Sn and pink-H.
